# Investigation of one‐stage meta‐analysis methods for joint longitudinal and time‐to‐event data through simulation and real data application

**DOI:** 10.1002/sim.7961

**Published:** 2018-09-12

**Authors:** Maria Sudell, Ruwanthi Kolamunnage‐Dona, François Gueyffier, Catrin Tudur Smith

**Affiliations:** ^1^ Department of Biostatistics University of Liverpool Liverpool UK; ^2^ Centre Hospitalier Universitaire de Lyon Lyon France

**Keywords:** joint model, longitudinal, meta‐analysis, simulation, time‐to‐event

## Abstract

**Background:** Joint modeling of longitudinal and time‐to‐event data is often advantageous over separate longitudinal or time‐to‐event analyses as it can account for study dropout, error in longitudinally measured covariates, and correlation between longitudinal and time‐to‐event outcomes. The current literature on joint modeling focuses mainly on the analysis of single studies with a lack of methods available for the meta‐analysis of joint data from multiple studies.

**Methods:** We investigate a variety of one‐stage methods for the meta‐analysis of joint longitudinal and time‐to‐event outcome data. These methods are applied to the INDANA dataset to investigate longitudinally measured systolic blood pressure, with each of time to death, time to myocardial infarction, and time to stroke. Results are compared to separate longitudinal or time‐to‐event meta‐analyses. A simulation study is conducted to contrast separate versus joint analyses over a range of scenarios.

**Results:** The performance of the examined one‐stage joint meta‐analytic models varied. Models that accounted for between study heterogeneity performed better than models that ignored it. Of the examined methods to account for between study heterogeneity, under the examined association structure, fixed effect approaches appeared preferable, whereas methods involving a baseline hazard stratified by study were least time intensive.

**Conclusions:** One‐stage joint meta‐analytic models that accounted for between study heterogeneity using a mix of fixed effects or a stratified baseline hazard were reliable; however, models examined that included study level random effects in the association structure were less reliable.

## 1 INTRODUCTION

Univariate shared random effect joint models for longitudinal and time‐to‐event data simultaneously model a single longitudinal and a single time‐to‐event outcome.^1^ The model consists of a longitudinal sub‐model and a time‐to‐event sub‐model linked through an association structure, which quantifies the relationship between the two outcomes. Many options are presented in the literature for each sub‐model (such as linear mixed effects models or splines for the longitudinal sub‐model, and proportional hazards or accelerated failure time models for the time‐to‐event sub‐model). A range of association structures exist,^2^ including sharing random effects between the sub‐models,^3^ sharing the current longitudinal trajectory (both the fixed and random effects), or sharing the first derivative of the longitudinal trajectory.^4^ The research presented here focuses on joint models that concern a single continuous longitudinal and a single possibly censored time‐to‐event outcome, linked using an association structure consisting of shared zero mean random effects with a common association parameter for random effects acting at the same level.^3^

Joint models for longitudinal and time‐to‐event data are often employed to account for study dropout and measurement error in time varying covariates, while producing less biased estimates of study parameters.^3^, ^5^ An example of their application compared to separate longitudinal models is presented by Powney et al,^6^ who discuss the MAGNETIC trial,^7^ which reported a longitudinal case with missing data where a complete case analysis found no significant difference between treatment groups, whereas the use of joint models to account for missing data resulted in a statistically significant difference. A recent review of current reporting of single study joint analyses by Sudell et al^8^ identified that the number of published joint analyses has been increasing over recent years, suggesting a growing resource of joint datasets. Examples of single study joint models applied in the literature include those of Jacoby et al,^9^ Kolamunnage‐Dona et al,^10^ Lloyd‐Williams et al,^11^ and Kovanda et al.^12^

Glass^13^ defined meta‐analysis (MA) as the statistical analysis or pooling of results from several studies. Meta‐analyses can result in analyses with increased precision and power, while permitting new research questions to be answered. An individual participant or patient data meta‐analysis (IPD‐MA) utilizes the original data collected in each study, whereas an aggregate data meta‐analysis (AD‐MA) utilizes study level results, including those available in published reports. IPD‐MA can be one‐stage or two‐stage. A two‐stage meta‐analysis fits models to the data from each study included in the meta‐analysis and, then, uses standard MA techniques^14^, ^15^ to pool the study specific parameter estimates. A one‐stage meta‐analysis stores the data from all studies included in the meta‐analysis in a single meta‐dataset, to which a single model is fitted (which should account for the clustering of data within studies).

The literature for meta‐analyses is extensive,^14^, ^15^ but research into the meta‐analysis of joint longitudinal and time‐to‐event data is limited to a small number of references.^8^, ^16^ However, it is reasonable that if joint modeling is preferred over separate longitudinal or time‐to‐event models in certain single study cases (eg, to account for informative dropout in longitudinal study designs^17^ or when a time‐to‐event outcome is influenced by longitudinal outcomes^18^), the use of joint models rather than separate methods may also be preferred in a meta‐analytic setting.

Currently, methodological research has mainly focused on joint models applied to single study datasets (for overviews, see the works of Tsiatis and Davidian^5^ and Yu et al^19^), although a limited number of references exist that deal with multi‐center joint data,^20^ and multi‐level joint models.^21^ However, these references did not specifically investigate the meta‐analytic case. Multi‐center and meta‐analytic datasets are similar, in that they have a structure where individuals are nested within studies or centers. However, the number of higher level units differs between cases; meta‐analyses often contain fewer studies, each containing a larger number of individuals, whereas multi‐center datasets often contain a larger number of centers, each containing a comparatively smaller number of individuals. As such, the spread of data across the different levels is different for a meta‐analytic dataset compared with a multi‐center dataset, leading to potentially different approaches being required. This paper extends this methodology by investigating multi‐level joint models specifically for use in meta‐analytic datasets.

Recently, Sudell et al^16^ investigated methods for the two‐stage MA of joint data. In this article, we investigate one‐stage models to analyze individual participant multi‐study joint longitudinal and time‐to‐event data (termed joint IPD). The results of the one‐stage meta‐analytic joint models are compared with one‐stage separate longitudinal or time‐to‐event meta‐analytic models. The article begins with a discussion of the methods employed in the investigation. The presented methods are then applied to an example dataset. A simulation study is then conducted to test the methods under a range of scenarios. The article concludes with a discussion of joint modeling methodology in one‐stage MA.

## 2 METHODS FOR ONE‐STAGE JOINT IPD‐MA

As mentioned, this research assumes the availability of joint longitudinal and time‐to‐event IPD. This IPD is considered to have three nested levels, namely, longitudinal measurements at level 1, nested within individuals at level 2, nested within studies at level 3. The joint models considered in this research assume a linear mixed effects model for the longitudinal outcome, and a Cox Proportional Hazards (PH) model with an unspecified baseline hazard for the time‐to‐event outcome. The two sub‐models are linked through shared zero mean random effects, with common association parameter (represented using ***α*** terms) for the random effects acting at the same level. Unlike joint models for single study data, the proposed models must account for the clustering of individuals within studies and model potential heterogeneity between these studies.

The one‐stage joint model follows the structure

(1) $$\begin{array}{cc} Y_{\mathrm{kij}}= & \;X_{1}\beta_{1}+Z_{\mathrm{ki}}^{\left(2\right)}b_{\mathrm{ki}}^{\left(2\right)}+Z_{k}^{\left(3\right)}b_{k}^{\left(3\right)}+\epsilon_{\mathrm{kij}}\\ \lambda_{\mathrm{ki}}\left(t\right)= & \;\lambda_{0}\left(t\right)\exp\;\left(X_{2}\beta_{2}+W_{2\mathrm{ki}}\left(t\right)\right)\\ W_{2\mathrm{ki}}\left(t\right)= & \;\alpha^{\left(2\right)}\left(Z_{\mathrm{ki}}^{\left(2\right)}b_{\mathrm{ki}}^{\left(2\right)}\right)+\alpha^{\left(3\right)}\left(Z_{k}^{\left(3\right)}b_{k}^{\left(3\right)}\right). \end{array}$$

Studies are identified by *k* = 1…*K*, where *K* is the total number of studies in the meta‐dataset. Individuals within each study are represented by *i* = 1…*n*_*k*_, where *n*_*k*_ denotes the total number of individuals in study *k*. The longitudinal measurement points are identified using *j* = 1…*m*_*ki*_, where *m*_*ki*_ represents the total number of longitudinal measurements recorded for individual *i* in study *k*.

The longitudinal measurement recorded for individual *i* in study *k* at time‐point *j* is represented by *Y*_*kij*_, with the longitudinal error term *ϵ*_*kij*_. Fixed effects are represented using ***β*** terms, with the first element of the subscript identifying the sub‐model they belong to (such that ***β***_**1**_ = *β*_11_, *β*_12_, *β*_13_, … are the longitudinal sub‐model fixed effects and ***β***_**2**_ = *β*_21_, *β*_22_, *β*_23_, … are the time‐to‐event sub‐model fixed effects). Random effects are represented by ***b***, with individual level (level 2) random effects represented by $b_{\mathrm{ki}}^{\left(2\right)}$ and study level (level 3) random effects by $b_{k}^{\left(3\right)}$. Design matrices are represented by ***X*** for the fixed effects and ***Z*** for the random effects. ***X***_**1**_ represents the longitudinal sub‐model fixed effects design matrix, and ***X***_**2**_ represents the time‐to‐event sub‐model fixed effects design matrix. Additionally, $Z_{\mathrm{ki}}^{\left(2\right)}$ represents the design matrix for the individual level (level 2) random effects, and $Z_{k}^{\left(3\right)}$ represents the design matrix for the study level (level 3) random effects.

The individual level random effects follow distribution $b_{\mathrm{ki}}^{\left(2\right)}∼N\left(0,D\right)$, whereas the study level random effects follow distribution $b_{k}^{\left(3\right)}∼N\left(0,A\right)$, and the error terms each follow distribution $\varepsilon_{\mathrm{kij}}∼N\left(0,\sigma_{e}^{2}\right)$. The individual level and the study level random effects are considered independent of each other and of the error terms. The random effects are intended to represent how covariate effects differ for units at the respective levels (individuals or studies) from those estimated for the overall population by the fixed effects, for example, how the individuals contained within a particular study differ from those in the overall population. As such, the ***Z*** matrices are assumed to be subsets of the ***X***_**1**_ matrix.

In the time‐to‐event sub‐model, *λ*_0_(*t*) represents the unspecified baseline hazard. The sub‐models are linked through shared zero mean random effects, with common association parameters *α*^(2)^ for the individual level random effects and *α*^(3)^ for the study level random effects. Note that if a particular component of the joint model is not required (eg, the study level random effects), terms involving this component (eg, $Z_{k}^{\left(3\right)}b_{k}^{\left(3\right)}$) do not appear in the model.

A range of model groups are investigated, which represent a variety of methods to account for between study heterogeneity. The specifications of the model groups are stated in Table 1. These models involve only longitudinal time (*t*_*kij*_), a binary treatment assignment variable (*treat*_*ki*_), and study membership (*study*_*ki*_) as covariates. However, the models examined can be easily extended if other covariates are of interest to the MA. Note that instances of longitudinal time *t*_*kij*_ in the association structure term *W*_2*ki*_(*t*) (which is present in the time‐to‐event sub‐model) are replaced by the individual's survival time *T*_*Ski*_.

**Table 1:** Specification of one‐stage model groups examined

*Model Group*	*Model Component*	*Equation*
**0**	**Longitudinal sub**‐**model**	*Y*_*kij*_ = β_10_ + β_11_*t*_*kij*_ + β_12_*treat*_*ki*_ $+b_{0\mathrm{ki}}^{\left(2\right)}+b_{1\mathrm{ki}}^{\left(2\right)}t_{\mathrm{kij}}+\varepsilon_{\mathrm{kij}}$
	**Time**‐**to**‐**event sub**‐**model**	*λ*_*ki*_(*t*) = *λ*_0_(*t*) exp (*β*_21_*treat*_*ki*_ + *W*_2*ki*_(*t*))
	**Association structure**	$W_{2\mathrm{ki}}\left(t\right)=\alpha^{\left(2\right)}\left(b_{0\mathrm{ki}}^{\left(2\right)}+b_{1\mathrm{ki}}^{\left(2\right)}T_{\mathrm{Ski}}\right)$
**1**	**Longitudinal sub**‐**model**	Y_*kij*_ = β_10_ + β_11_*t*_*kij*_ + β_12_*treat*_*ki*_ + β_13_*study*_*ki*_ + β_14_*treat*_*ki*_ ∗ *study*_*ki*_ $+b_{0\mathrm{ki}}^{\left(2\right)}+b_{1\mathrm{ki}}^{\left(2\right)}t_{\mathrm{kij}}+\varepsilon_{\mathrm{kij}}$
	**Time‐to‐event sub‐model**	*λ*_*ki*_(*t*) = *λ*_0_(*t*) exp (*β*_21_*treat*_*ki*_ + *β*_22_*study*_*ki*_ + *β*_23_*treat*_*ki*_ ∗ *study*_*ki*_ + *W*_2*ki*_(*t*))
	**Association structure**	$W_{2\mathrm{ki}}\left(t\right)=\alpha^{\left(2\right)}\left(b_{0\mathrm{ki}}^{\left(2\right)}+b_{1\mathrm{ki}}^{\left(2\right)}T_{\mathrm{Ski}}\right)$
**2**	**Longitudinal sub‐model**	*Y*_*kij*_ = β_10_ + β_11_*t*_*kij*_ + β_12_*treat*_*ki*_ + β_13_*study*_*ki*_ $+b_{0\mathrm{ki}}^{\left(2\right)}+b_{1\mathrm{ki}}^{\left(2\right)}t_{\mathrm{kij}}$ $+b_{1k}^{\left(3\right)}\text{trea}t_{\mathrm{ki}}+\varepsilon_{\mathrm{kij}}$
	**Time‐to‐event sub‐model**	*λ*_*ki*_(*t*) = *λ*_0_(*t*) exp (*β*_21_*treat*_*ki*_ + *β*_22_*study*_*ki*_ + *W*_2*ki*_(*t*))
	**Association structure**	$W_{2\mathrm{ki}}\left(t\right)=\alpha^{\left(2\right)}\left(b_{0\mathrm{ki}}^{\left(2\right)}+b_{1\mathrm{ki}}^{\left(2\right)}T_{\mathrm{Ski}}\right)+\alpha^{\left(3\right)}\left(b_{1k}^{\left(3\right)}\text{trea}t_{\mathrm{ki}}\right)$
**3**	**Longitudinal sub‐model**	*Y*_*kij*_ = β_10_ + β_11_*t*_*kij*_ + β_12_*treat*_*ki*_ $+b_{0\mathrm{ki}}^{\left(2\right)}+b_{1\mathrm{ki}}^{\left(2\right)}t_{\mathrm{kij}}$ $+b_{0k}^{\left(3\right)}+b_{1k}^{\left(3\right)}\text{trea}t_{\mathrm{ki}}+\varepsilon_{\mathrm{kij}}$
	**Time‐to‐event sub‐model**	*λ*_*ki*_(*t*) = *λ*_0_(*t*) exp (*β*_21_*treat*_*ki*_ + *W*_2*ki*_(*t*))
	**Association structure**	$W_{2\mathrm{ki}}\left(t\right)=\alpha^{\left(2\right)}\left(b_{0\mathrm{ki}}^{\left(2\right)}+b_{1\mathrm{ki}}^{\left(2\right)}T_{\mathrm{Ski}}\right)+\alpha^{\left(3\right)}\left(b_{0k}^{\left(3\right)}+b_{1k}^{\left(3\right)}\text{trea}t_{\mathrm{ki}}\right)$
**4**	**Longitudinal sub‐model**	*Y*_*kij*_ = β_10_ + β_11_*t*_*kij*_ + β_12_*treat*_*ki*_ + β_13_*study*_*ki*_ + β_14_*treat*_*ki*_ ∗ *study*_*ki*_ $+b_{0\mathrm{ki}}^{\left(2\right)}+b_{1\mathrm{ki}}^{\left(2\right)}t_{\mathrm{kij}}+\varepsilon_{\mathrm{kij}}$
	**Time‐to‐event sub‐model**	*λ*_*ki*_(*t*) = *λ*_0*k*_(*t*) exp (*β*_21_*treat*_*ki*_ + *W*_2*ki*_(*t*))
	**Association structure**	$W_{2\mathrm{ki}}\left(t\right)=\alpha^{\left(2\right)}\left(b_{0\mathrm{ki}}^{\left(2\right)}+b_{1\mathrm{ki}}^{\left(2\right)}T_{\mathrm{Ski}}\right)$
**5**	**Longitudinal sub‐model**	*Y*_*kij*_ = β_10_ + β_11_*t*_*kij*_ + β_12_*treat*_*ki*_ +β_13_*study*_*ki*_ $+b_{0\mathrm{ki}}^{\left(2\right)}+b_{1\mathrm{ki}}^{\left(2\right)}\mathrm{tim}e_{\mathrm{kij}}$ $+b_{1k}^{\left(3\right)}\text{trea}t_{\mathrm{ki}}+\varepsilon_{\mathrm{kij}}$
	**Time‐to‐event sub‐model**	*λ*_*ki*_(*t*) = *λ*_0*k*_(*t*) exp (*β*_21_*treat*_*ki*_ + *W*_2*ki*_(*t*))
	**Association structure**	$W_{2\mathrm{ki}}\left(t\right)=\alpha^{\left(2\right)}\left(b_{0\mathrm{ki}}^{\left(2\right)}+b_{1\mathrm{ki}}^{\left(2\right)}T_{\mathrm{Ski}}\right)+\alpha^{\left(3\right)}\left(b_{1k}^{\left(3\right)}\text{trea}t_{\mathrm{ki}}\right)$

Model group 0 in Table 1 is a naïve model which does not account for between study heterogeneity in any way. This model is presented here to highlight the consequence of ignoring the clustered nature of multi‐study joint data. Note that any instances of longitudinal time in the association structure are replaced with the individual's survival time (denoted *T*_*Ski*_, equal to the minimum of their event and censoring times).

Model group 1 accounts for between study heterogeneity using a fixed study membership variable, along with its interaction with treatment assignment, in both sub‐models. Study membership is expected to be a factor variable, and so a separate *β*_13_, *β*_14_, *β*_22_, and *β*_23_ parameter will be produced for each study *k* in the meta‐analysis (apart from the reference or baseline study), denoted *β*_13*k*_, *β*_14*k*_, *β*_22*k*_, and *β*_23*k*_. The study considered to be the reference study should be the representative of the population of interest. In model group 1, inclusion of the fixed study membership variable allows calculation of study specific fixed longitudinal trajectory intercepts (with *β*_10_ representing the fixed intercept for the reference study, and *β*_10_ + *β*_14*k*_ for non‐reference study *k*). Likewise, study specific longitudinal treatment effects can be calculated (with *β*_13_ representing the fixed longitudinal treatment effect for the reference study, and *β*_13_ + *β*_15*k*_ for non‐reference study *k*). In the time‐to‐event sub‐model, the *β*_22*k*_ parameter represents the difference in risk of an event between study *k*, and the reference study. The deviation in risk of an event due to treatment group is equal to *β*_21_ for the reference study, and by *β*_21_ + *β*_23k_ for non‐reference study *k*.

Model group 2 accounts for between study heterogeneity using a fixed study membership variable in both sub‐models, and a study level zero‐mean random treatment effect ( $b_{1k}^{\left(3\right)}$). Study specific longitudinal trajectory intercepts and log‐hazard ratio risks of an event for each study can be calculated from the fixed effects as for model group 1. The interpretation of the study specific random treatment effect $b_{1k}^{\left(3\right)}$is more complex than for separate longitudinal or time‐to‐event one‐stage MA‐models due to its presence in both sub‐models. In the longitudinal sub‐model, the $b_{1k}^{\left(3\right)}$ term adjusts the overall population treatment effect coefficient *β*_12_ to give the observed treatment effect in study *k* of $\beta_{12}+b_{1k}^{\left(3\right)}$. Through the association structure, $b_{1k}^{\left(3\right)}$ is present in the time‐to‐event sub‐model. As such, the population treatment effect coefficient *β*_21_ is altered to give a study specific estimate of the deviation in the risk of an event due to treatment group ( $\beta_{21}+\alpha^{\left(3\right)}b_{1k}^{\left(3\right)}$).

Model group 3 accounts for between study heterogeneity solely using study level random effects, as it involves a study level random intercept ( $b_{0k}^{\left(3\right)}$) and random treatment effect ( $b_{1k}^{\left(3\right)})$. Again, the interpretation of these random effects is more complex than for separate one‐stage longitudinal or time‐to‐event MA‐models due to their presence in both sub‐models through the association structure. The study level random intercept $b_{0k}^{\left(3\right)}$ causes the longitudinal intercept for study *k* to equal *β*_10_+ $b_{0k}^{\left(3\right)}$, but also $\alpha^{\left(3\right)}b_{0k}^{\left(3\right)}$ represents the deviation in the risk of an event in the *k*th study from the population average taken across all studies in the meta‐analysis. The interpretation of the random treatment effect ( $b_{1k}^{\left(3\right)})$ is the same as for model group 2.

Model group 4 has a longitudinal sub‐model with the same specification (and so interpretation) as model group 1. However, the baseline hazard in the time‐to‐event sub‐model is stratified by study (*λ*_0*k*_(*t*)), and the time‐to‐event sub‐model contains only a fixed treatment assignment term. As such, between study heterogeneity in the time‐to‐event model is captured by the study specific baseline hazards.

Model group 5 accounts for between study heterogeneity in a variety of ways. A fixed study membership term is included in the longitudinal sub‐model, a study level random treatment effect ( $b_{1k}^{\left(3\right)})$ is present in both sub‐models through the association structure, and the baseline hazard of the time‐to‐event sub‐model is stratified by study. Each component of the model has interpretations as already discussed.

In addition to the one‐stage joint MA‐models, we also fit separate longitudinal and time‐to‐event one‐stage MA‐models for the comparison with the joint estimates. These separate models have the same specification as the corresponding joint model sub‐models, except for the *W*_2*ki*_(*t*) term that is removed from the time‐to‐event one‐stage MA‐models.

## 3 MODEL FITTING

The models described in Section 2 were fitted using the Expectation Maximization (EM) algorithm,^22^ whose use in single study joint modeling analyses has been described by Wulfsohn and Tsiatis^1^ and Rizopoulos.^4^ Starting values for the algorithm were extracted from initial separate longitudinal and time‐to‐event model fits (of the same specification as the corresponding sub‐models of the joint model, excluding the association structure). In the Expectation or E‐step, estimates of functions of random effects were calculated using pseudo‐adaptive Gaussian quadrature procedures,^23^ where conditional modes of the random effects calculated in the initial separate longitudinal model fit were used to calculate appropriate locations for the abscissa to be used throughout the model fitting process. In the Maximization or M‐step, these estimated functions of the random effects were used to calculate maximum likelihood estimates of model parameters. The derived maximum likelihood estimators have been made available as Supplemental Material.

## 4 SOFTWARE

We developed a flexible R^24^ code to fit one‐stage multi‐study joint models described in this article, which will be available as joineRmeta package; the R codes can currently be downloaded at https://github.com/mesudell/joineRmeta/. This software is an extension of the single study joint modeling package joineR^25^ to the multi‐study case. Example code and simulated data are available in the supplemental information, demonstrating methods discussed in this article.

## 5 APPLICATION

### 5.1 Example data

To investigate the behavior of the proposed methods in a real world scenario, the methods were applied to a subset of the INDANA dataset.^26^ This is a multi‐study dataset compiled to investigate the effect of patient characteristics on the efficacy of pharmacological treatment for high blood pressure. The subset analyzed here (henceforward referred to as the INDANA dataset) contains any study identified by the INDANA collaboration^26^ that supplied both longitudinal and time‐to‐event data and contains 6 studies (EWPHE,^27^ COOP,^28^ STOP,^29^ SHEP,^30^ MRC1,^31^ and MRC2^32^). The INDANA dataset concerns hypertensive patients assigned to one of two treatment groups, that is, any treatment for hypertension versus placebo, no treatment, or usual care. Longitudinally measured Systolic and Diastolic Blood Pressure were available, referred to as SBP and DBP. Three time‐to‐event outcomes were measured, namely, time to death, time to myocardial infarction (MI), and time to stroke.

The data contained 9 possible longitudinal time‐points at baseline, 6 months, 1 year, and annually thereafter to a maximum of 7 years. The SHEP study recorded individuals at only 6 measurement times, while STOP and MRC1 presented 7 measurement times, with the remaining studies presenting data at each of the 9 possible measurement times. Only longitudinal data recorded prior to an individual's survival time contributed to the analyses. Tables of the number of measurements provided by each study at each time point are available in the supplemental information (supplemental tables S1‐S3).

Analyses of SBP and each time‐to‐event outcome are presented in Tables 2 to 4. For EWPHE, an intention to treat analysis was only possible for fatal endpoints, and so the study only contributes to the analysis of SBP and time to death. As such, the final dataset examined contained a maximum of 6 studies totalling at most 29825 individuals. The exact number of individuals involved in each analysis is stated in the captions of Tables 2 to 4.

**Table 2:** One‐stage joint and separate model results for analysis of SBP and time to death by model group (dataset contains 29825 individuals, 2082 events, and 162574 longitudinal measurements)

*SBP and Time to Death*								
*Model*	*Longitudinal Treatment Effect Parameter(s)*			*Time‐to‐Event Treatment Effect Parameter(s)*			*Association Parameters*	
*Group*		Separate Model	Joint Sub‐Model		Separate Model	Joint Sub‐Model		Joint Sub‐Model
		Results	Results		Results	Results		Results
***0***	*β*_12_	**−9.52 (−9.90, −9.13)**	−**9.52 (−9.92, −9.19)**	*β*_21_	−0.09 (−0.17, 0.00)	−0.02 (−0.13, 0.07)	α^(2)^	**0.032 (0.029, 0.035)**
***1***	β_12*COOP*_	**−10.03 (−11.74, −8.33)**	**−10.04 (−12.39, −7.91)**	β_21*COOP*_	−0.07 (−0.18, 0.04)	0.02 (−0.37, 0.41)		
	β_12*EWPHE*_	**−13.15 (−16.56, −9.74)**	**−13.15 (−15.24, −11.10)**	β_21*EWPHE*_	−0.13 (−0.35, 0.09)	−0.03 (−0.31, 0.25)		
	β_12*MRC*1_	**−7.78 (−9.57, −5.99)**	**−7.78 (−8.17, −7.42)**	β_21*MRC*1_	−0.06 (−0.48, 0.37)	0.00 (−0.16, 0.15)		
	β_12*MRC*2_	**−10.72 (−11.10, −10.34)**	**−10.72 (−11.33, −10.07)**	β_21*MRC*2_	−0.07 (−0.51, 0.37)	−0.01 (−0.16, 0.16)	α^(2)^	**0.013 (0.009, 0.019)**
	β_12*SHEP*_	**−8.30 (−9.06, −7.55)**	**−8.31 (−8.88, −7.75)**	β_21*SHEP*_	−0.16 (−0.56, 0.23)	−0.11 (−0.31, 0.09)		
	β_12*STOP*_	**−14.16 (−14.91, −13.40)**	**−14.16 (−15.40, −12.93)**	β_21*STOP*_	**−0.54 (−0.95, −0.13)**	**−0.49 (−0.95, −0.14)**		
***2***	*β*_12_	**−10.62 (−12.68, −8.57)**	**−10.63 (−11.18, −9.97)**	*β*_21_	−0.08 (−0.17, 0.00)	−0.05 (−0.15, 0.04)	α^(2)^	**0.013 (0.008, 0.018)**
							α^(3)^	0.000 (−0.043, 0.052)
***3***	*β*_12_	**−10.67 (−12.67, −8.67)**	**−2.70 (−3.09, −2.42)**	*β*_21_	−0.09 (−0.17, 0.00)	−0.05 (−0.14, 0.03)	α^(2)^	**0.011 (0.007, 0.016)**
							α^(3)^	**0.052 (0.049, 0.055)**
***4***	β_12*COOP*_	**−10.03 (−11.74, −8.33)**	**−10.04 (−12.31, −8.07)**					
	β_12*EWPHE*_	**−13.15 (−16.56, −9.74)**	**−13.15 (−15.32, −11.19)**					
	β_12*MRC*1_	**−7.78 (−9.57, −5.99)**	**−7.78 (−8.23, −7.43)**					
	β_12*MRC*2_	**−10.72 (−11.10, −10.34)**	**−10.72 (−11.36, −10.11)**	*β*_21_	−0.08 (−0.17, 0.00)	−0.06 (−0.13, 0.03)	α^(2)^	**0.013 (0.008, 0.017)**
	β_12*SHEP*_	**−8.30 (−9.06, −7.55)**	**−8.30 (−8.94, −7.59)**					
	β_12*STOP*_	**−14.16 (−14.91, −13.40)**	**−14.15 (−15.25, −12.90)**					
***5***	*β*_12_	**−10.62 (−12.68, −8.57)**	**−10.63 (−11.17, −10.06)**	*β*_21_	−0.08 (−0.17, 0.00)	−0.06 (−0.14, 0.03)	α^(2)^	**0.013 (0.006, 0.017)**
							α^(3)^	0.000 (−0.039, 0.048)

**Table 3:** One‐stage joint and separate model results for analysis of SBP and time to MI by model group (dataset contains 28977 individuals, 1124 events, and 157923 longitudinal measurements)

*SBP and Time to MI*								
*Model*	*Longitudinal Treatment Effect Parameter(s)*			*Time‐to‐Event Treatment Effect Parameter(s)*			*Association Parameters*	
*Group*		Separate Model	Joint Sub‐Model		Separate Model	Joint Sub‐Model		Joint Sub‐Model
		Results	Results		Results	Results		Results
***0***	*β*_12_	**−9.45 (−9.84, −9.07)**	**−9.46 (−9.82, −8.98)**	*β*_21_	**−0.16 (−0.28–0.04)**	**−0.13 (−0.24, −0.02)**	α^(2)^	**0.027 (0.023, 0.031)**
***1***	β_12*COOP*_	**−10.18 (−11.88, −8.48)**	**−10.18 (−12.65, −8.08)**	β_21*COOP*_	−0.13 (−0.26, 0.00)	0.11 (−0.43, 0.53)		
	β_12*MRC*1_	**−7.80 (−11.20, −4.41)**	**−7.80 (−8.22, −7.35)**	β_21*MRC*1_	−0.22 (−0.47, 0.03)	−0.03 (−0.21, 0.14)		
	β_12*MRC*2_	**−10.78 (−11.16, −10.40)**	**−10.79 (−11.44, −10.13)**	β_21*MRC*2_	−0.37 (−0.91, 0.16)	−0.18 (−0.44, 0.06)	α^(2)^	**0.020 (0.013, 0.025)**
	β_12*SHEP*_	**−8.39 (−9.15, −7.64)**	**−8.40 (−8.96, −7.72)**	β_21*SHEP*_	−0.48 (−1.01, 0.06)	**−0.29 (−0.57, −0.07)**
	β_12*STOP*_	**−14.28 (−15.03, −13.52)**	**−14.28 (−15.99, −13.02)**	β_21*STOP*_	−0.39 (−0.94, 0.16)	−0.21 (−0.78, 0.23)		
***2***	*β*_12_	**−10.25 (−12.48, −8.01)**	**−10.25 (−10.84, −9.65)**	*β*_21_	**−0.16 (−0.27, −0.04)**	**−0.12 (−0.25, −0.01)**	α^(2)^	**0.019 (0.013, 0.026)**
							α^(3)^	−0.036 (−0.105, 0.027)
***3***	*β*_12_	**−10.35 (−12.55, −8.16)**	**−2.54 (−2.88, −2.16)**	*β*_21_	**−0.16 (−0.28, −0.04)**	**−0.13 (−0.25, −0.01)**	α^(2)^	**0.019 (0.013, 0.025)**
							α^(3)^	**0.034 (0.028, 0.038)**
***4***	β_12*COOP*_	**−10.18 (−11.88, −8.48)**	**−10.20 (−12.25, −7.94)**					
	β_12*MRC*1_	**−7.80 (−11.20, −4.41)**	**−7.81 (−8.23, −7.33)**					
	β_12*MRC*2_	**−10.78 (−11.16, −10.40)**	**−10.78 (−11.46, −10.22)**	*β*_21_	**−0.16 (−0.27, −0.04)**	**−0.12 (−0.26, −0.01)**	α^(2)^	**0.019 (0.013, 0.025)**
	β_12*SHEP*_	**−8.39 (−9.15, −7.64)**	**−8.39 (−9.00, −7.79)**					
	β_12*STOP*_	**−14.28 (−15.03, −13.52)**	**−14.28 (−15.67, −13.08)**					
***5***	*β*_12_	**−10.25 (−12.48, −8.01)**	**−10.25 (−10.76, −9.65)**	*β*_21_	**−0.16 (−0.27, −0.04)**	**−0.12 (−0.24, −0.01)**	α^(2)^	**0.019 (0.013, 0.025)**
							α^(3)^	**−**0.036 (−0.098, 0.034)

**Table 4:** One‐stage joint and separate model results for analysis of SBP and time to stroke by model group (dataset contains 28985 individuals, 808 events, and 157834 longitudinal measurements)

*SBP and Time to Stroke*								
*Model*	*Longitudinal Treatment Effect Parameter(s)*			*Time‐to‐Event Treatment Effect Parameter(s)*			*Association Parameters*	
*Group*		Separate Model	Joint Sub‐Model		Separate Model	Joint Sub‐Model		Joint Sub‐Model
		Results	Results		Results	Results		Results
***0***	*β*_12_	**−9.43 (−9.82, −9.05)**	**−9.44 (−9.87, −9.08)**	*β*_21_	**−0.46 (−0.60, −0.32)**	**−0.39 (−0.53, −0.27)**	α^(2)^	**0.044 (0.040, 0.048)**
***1***	β_12*COOP*_	**−9.98 (−11.68, −8.28)**	**−9.98 (−11.84, −7.76)**	β_21*COOP*_	**−0.53 (−0.73, −0.33)**	**−0.46 (−1.08, −0.01)**		
	β_12*MRC*1_	**−7.79 (−11.20, −4.39)**	**−7.79 (−8.18, −7.39)**	β_21*MRC*1_	**−0.56 (−0.96, −0.16)**	**−0.55 (−0.86, −0.28)**		
	β_12*MRC*2_	**−10.74 (−11.12, −10.36)**	**−10.74 (−11.50, −10.10)**	β_21*MRC*2_	−0.23 (−0.96, 0.49)	−0.22 (−0.49, 0.06)	α^(2)^	**0.034 (0.027, 0.041)**
	β_12*SHEP*_	**−8.39 (−9.14, −7.63)**	**−8.40 (−9.00, −7.83)**	β_21*SHEP*_	−0.41 (−1.04, 0.23)	**−0.40 (−0.61, −0.17)**		
	β_12*STOP*_	**−14.24 (−15.00, −13.49)**	**−14.25 (−15.71, −12.88)**	β_21*STOP*_	−0.59 (−1.22, 0.03)	**−0.59 (−1.06, −0.16)**		
***2***	*β*_12_	**−10.19 (−12.41, −7.96)**	**−10.19 (−10.75, −9.60)**	*β*_21_	**−0.46 (−0.60, −0.32)**	**−0.40 (−0.57, −0.27)**	α^(2)^	**0.034 (0.026, 0.042)**
							α^(3)^	−0.077 (−0.189, 0.003)
***3***	*β*_12_	**−10.29 (−12.48, −8.11)**	**−2.52 (−2.93, −2.15)**	*β*_21_	**−0.46 (−0.60, −0.32)**	**−0.40 (−0.55, −0.26)**	α^(2)^	**0.030 (0.023, 0.038)**
							α^(3)^	**0.056 (0.051, 0.060)**
***4***	β_12*COOP*_	**−9.98 (−11.68, −8.28)**	**−9.97 (−12.30, −7.48)**					
	β_12*MRC*1_	**−7.79 (−11.20, −4.39)**	**−7.79 (−8.18, −7.36)**					
	β_12*MRC*2_	**−10.74 (−11.12, −10.36)**	**−10.75 (−11.41, −10.04)**	*β*_21_	**−0.46 (−0.60, −0.32)**	**−0.40 (−0.52, −0.28)**	α^(2)^	**0.034 (0.026, 0.042)**
	β_12*SHEP*_	**−8.39 (−9.14, −7.63)**	**−8.40 (−9.06, −7.69)**					
	β_12*STOP*_	**−14.24 (−15.00, −13.49)**	**−14.24 (−15.65, −12.92)**					
***5***	*β*_12_	**−10.19 (−12.41, −7.96)**	**−10.19 (−10.70, −9.66)**	*β*_21_	**−0.46 (−0.60, −0.32)**	**−0.40 (−0.55, −0.29)**	α^(2)^	**0.034 (0.027, 0.041)**
							α^(3)^	−0.076 (−0.171, 0.005)

The aim of this investigation was to illustrate the proposed one‐stage joint meta‐analytic models, rather than to investigate potential treatment modifiers. As such, while the INDANA dataset contained a range of patient covariates that could influence the outcomes, models in this investigation included only treatment assignment, study membership, and the longitudinal time covariate.

The models of specification shown in Table 1 were fitted to the data for each combination of outcomes (SBP and each of time to death, time to MI, and time to stroke, with longitudinal outcome *Y*_*kij*_ = *SBP*_*kij*_). However, plots of the longitudinal trajectories for each study paneled by event type (Supplemental Figures S1‐S3) indicated a changepoint early in the trajectories. A range of terms were tested to account for non‐linearity due to the changepoint including $\;t_{\mathrm{kij}}^{2}$, exp(−*t*_*kij*_) and exp(−*a* ∗ *t*_*kij*_). Comparison of the log‐likelihoods and AIC values of the models determined that inclusion of the term exp(−3 ∗ *t*_*kij*_) gave the best fit. Consequently, in addition to the terms stated in Table 1, each longitudinal sub‐model also contained a exp(−3 ∗ *t*_*kij*_) term (for clarity, full model specifications for real data analyses are available in Supplemental Table S4).

In the models examined, a statistically significant negative treatment assignment coefficient in the time‐to‐event model would indicate that assignment to any treatment for hypertension versus placebo, no treatment, or usual care significantly reduced the risk of the event in question. Model groups 0, 2, 3, 4, and 5 each produce a single global time‐to‐event treatment effect estimate (*β*_21_), whereas model group 1 produces study specific treatment effect estimates (calculated by *β*_21_ for the reference study, and by *β*_21_ + *β*_23*k*_ for non‐reference study *k*).

A statistically significant negative treatment assignment coefficient in the longitudinal sub‐model would indicate that assignment to any treatment for hypertension significantly decreased SBP. Model groups 0, 2, 3, and 5 each produce a single global longitudinal treatment effect estimate (*β*_12_), whereas model groups 1 and 4 produce study specific estimates (calculated by *β*_12_ for the reference study, and *β*_12_ + *β*_14*k*_ for non‐reference study *k*).

A statistically significant positive study level association parameter (*α*^(3)^) indicates that individuals in studies with longitudinal outcome values above the corresponding overall population mean are at higher risk of experiencing the event at a given time point. A statistically significant positive individual level association parameter (*α*^(2)^) indicates that individuals with longitudinal values above that predicted by the terms in the longitudinal sub‐model (apart from the individual level random effects) are at higher risk of experiencing the event at a given time point. Association parameters were only estimated for joint analyses.

### 5.2 Results from the INDANA dataset meta‐analyses

Tables 2–4 present the results of application of model groups 0–5 (as stated in Supplemental Table S4) to the INDANA dataset. Graphical representations of these results are shown in Supplemental Figures S4–S12.

Across all pairwise combinations of outcomes investigated, the estimated treatment effect from the separate longitudinal one‐stage IPD‐MA and the joint one‐stage IPD‐MA longitudinal sub‐model was significant and negative, indicating that assignment to treatment for hypertension significantly reduced SBP compared to placebo, no treatment, or usual care. The estimated treatment effect from the separate and joint analyses agreed well across model groups examined, apart from model group 3 (which solely accounted for between study heterogeneity using study level random effects). Here, the separate results were similar to those produced by the other model groups; however, the results from the joint analysis, while still significant, were much smaller in magnitude than the joint results from the other modeling groups. In the separate group 3 model, the study level random effects accounted for between study heterogeneity in the longitudinal trajectory. However, in the joint model, they also accounted for between study heterogeneity in the time‐to‐event sub‐model through their presence in the association structure. It was important to determine if sharing study level random effects in this way between sub‐models caused bias in covariate estimates, examined through simulations in Section 5.

Throughout the analyses, the estimated time‐to‐event treatment coefficient from the joint one‐stage IPD‐MA models was smaller in magnitude than those from the separate one‐stage IPD‐MA model. However, the direction of the results agreed between the separate and the joint analyses. For SBP and time to death, the separate and joint analyses agreed in the significance of results, with a significant reduction in risk of death due to assignment to any treatment for hypertension estimated only for the STOP trial for model group 1. For SBP and time to MI, model groups 0, 2, 3, 4, and 5 for both the separate and joint analyses estimated significant negative global treatment effect estimates, indicating a significant reduction in risk of MI due to assignment to treatment for hypertension. However, for model group 1, only the study specific estimate for the SHEP trial from the joint analysis was significant. For SBP and time to stroke, model groups 0, 2, 3, 4, and 5 for both the separate and joint analyses estimated significant negative global treatment effect estimates, indicating a significant reduction in risk of stroke due to assignment to treatment for hypertension. These treatment assignment coefficients were larger in magnitude than the results for time to death or time to MI. For model group 1, the separate time‐to‐event model identified study specific significant treatment effects for COOP and MRC1; however, the joint analysis additionally identified significant effects for SHEP and STOP.

Individual level random effects were included in all model groups examined, causing the individual level association parameter *α*^(2)^ to be present in all model groups. For each set of outcomes examined, all model groups estimated significant positive values for *α*^(2)^, indicating that individuals with SBP values above the corresponding population average are at higher risk of an event. We should note that model group 0 consistently estimated *α*^(2)^ values of larger magnitude than the other model groups (which were consistent in the magnitude of *α*^(2)^ estimated). This highlights the importance of accounting for between study heterogeneity in joint analyses of multi‐study data.

Study level random effects were only employed in model groups 2, 3, and 5, meaning that the study level association parameter *α*^(3)^ was only estimated in these model groups. There was a noticeable discrepancy between results from model group 3 and model groups 2 or 5. Model group 3 contained both a study level random intercept and treatment effect, whereas model groups 2 and 5 contained only a study level random treatment effect. Model group 3 estimated a significant positive study level association parameter across all three sets of analyses (with interpretation that studies with SBP values above the population average were at higher risk of an event). However, as noted earlier, for the joint analysis, estimated parameters from model group 3 were inconsistent with the results produced by the other model groups. Model groups 2 and 5 estimated insignificant *α*^(3)^ values across the three sets of analyses, which were different in magnitude to model group 3, and had wide confidence intervals. These results motivated a simulation study to investigate when the use of shared study level random effects may be recommended.

## 6 SIMULATION INVESTIGATIONS

In practice, meta‐analyses involve data with very different characteristics to those displayed in our real data example. For example, associations between the longitudinal and time‐to‐event outcomes may be different in significance and/or magnitude. The number of studies included in the meta‐analysis might differ. There might be a different level of variability or heterogeneity between studies involved in the meta‐analysis. To assess the behavior of the models stated in Table 1 under a range of these different conditions, a range of simulation investigations were conducted. These simulations can be split into three main sets: Simulation Set 1 investigates the models under different levels of association, Simulation Set 2 investigates differing numbers of studies included in the meta‐analysis, and Simulation Set 3 investigates differing levels of between study heterogeneity. During the simulation investigations, data were firstly simulated using the models and methods discussed in Section 6.1. The models stated in Table 1 were then fitted to each simulated dataset, the results of which are presented in Section 6.2.

### 6.1 Data simulation

Data for each set of simulations were simulated under the same model structure, but with different model parameter values, which we will now describe. For each set of simulations, for each scenario, 1000 datasets were simulated.

For each dataset within each set of simulations, multi‐study joint data were generated containing a single continuous normally distributed longitudinal outcome and a single censored time‐to‐event outcome. The number of included studies varies between simulation sets; however, each simulated study contained 500 individuals randomized equally to two treatment groups. A maximum of 10 longitudinal measurements at times 0, 0.25, 0.5, 1, 1.5, 2, 2.5, 3, 3.5, and 4 were permitted, with measurements recorded only up to the individual's survival time (*T*_*Ski*_). Data for all studies were simulated simultaneously, with any between study heterogeneity generated through specification of the distribution of study level random effects. The longitudinal data were simulated under Equation (2).

(2) $$Y_{\mathrm{kij}}=\beta_{10}+\beta_{11}t_{\mathrm{kij}}+\beta_{12}\text{trea}t_{\mathrm{ki}}+b_{0\mathrm{ki}}^{\left(2\right)}+b_{1\mathrm{ki}}^{\left(2\right)}t_{\mathrm{kij}}+b_{0k}^{\left(3\right)}+b_{1k}^{\left(3\right)}\text{trea}t_{\mathrm{ki}}+\varepsilon_{\mathrm{kij}}$$

In Equation (2), the longitudinal outcome *Y*_*kij*_ follows a linear mixed effects model containing fixed intercept, time and treatment assignment terms (with coefficients *β*_10_, *β*_11_, and *β*_12_), individual level random intercept and slope terms ( $b_{0\mathrm{ki}}^{\left(2\right)}$ and $b_{1\mathrm{ki}}^{\left(2\right)}$), study level random intercept and treatment effect terms ( $b_{0k}^{\left(3\right)}$ and $b_{1k}^{\left(3\right)}$), and an error term *ϵ*_*kij*_. The random effects follow multivariate normal distributions, with individual level random effects distributed $\;b_{\mathrm{ki}}^{\left(2\right)}\;∼\;N\left(0,D\right)$, and study level random effects distributed $\;b_{k}^{\left(3\right)}\;∼\;N\left(0,A\right)$. The random effects are independent of each other and of the error terms, which are considered to be independently and identically distributed $\varepsilon_{\mathrm{kij}}\;∼\;N(0,\sigma_{e}^{2}$).

The simulation of time‐to‐event data under a proportional hazards model with time varying covariates is described by Bender et al^33^ and Austin.^34^ In these simulations, the time‐to‐event data were generated under Equation (3), where *λ*_0_(*t*) is an unspecified baseline hazard.

(3) $$\begin{array}{cc} \lambda_{\mathrm{ki}}\left(t\right)= & \;\lambda_{0}\left(t\right)\exp\left(\beta_{21}\text{trea}t_{\mathrm{ki}}+W_{2\mathrm{ki}}\left(t\right)\right)\\ W_{2\mathrm{ki}}\left(t\right)= & \;\alpha^{\left(2\right)}W_{1\mathrm{ki}}\left(t\right)=\alpha^{\left(2\right)}\left(b_{0\mathrm{ki}}^{\left(2\right)}+b_{1\mathrm{ki}}^{\left(2\right)}T_{\mathrm{Ski}}\right)+\alpha^{\left(3\right)}\left(b_{0k}^{\left(3\right)}+b_{1k}^{\left(3\right)}\text{trea}t_{\mathrm{ki}}\right) \end{array}$$

As a time varying covariate is present in the time‐to‐event sub‐model (the individual level random time term $b_{1\mathrm{ki}}^{\left(2\right)}$, present through the association structure), event times are simulated under a Gompertz distribution, as it has a baseline hazard that can vary over time. Consequently, individual event times *T*_*Eki*_ are generated under Equation (4) (where $Z_{\mathrm{ki}}^{\left(3\right)}b_{k}^{\left(3\right)}=b_{0k}^{\left(3\right)}+b_{1k}^{\left(3\right)}\text{trea}t_{\mathrm{ki}}$).

(4) $$T_{\mathrm{Eki}}=\frac{1}{\alpha^{\left(2\right)}b_{1\mathrm{ki}}^{\left(2\right)}+\theta_{1}}\log\left[1+\frac{\left(\alpha^{\left(2\right)}b_{1\mathrm{ki}}^{\left(2\right)}+\theta_{1}\right)\left(-\log\left(U_{\mathrm{ki}}\right)\right)}{\exp\;\left(\theta_{0}+\beta_{21}\text{trea}t_{\mathrm{ki}}+\alpha^{\left(2\right)}b_{0k}^{\left(2\right)}+\alpha^{\left(3\right)}\left(Z_{\mathrm{ki}}^{\left(3\right)}b_{k}^{\left(3\right)}\right)\right)}\right]$$

In Equation (4), *U*_*ki*_ is an individual specific realization from a Uniform *U*(0, 1) distribution. The parameters *θ*_0_ (the exponential of which is the scale parameter of a Gompertz distribution) and *θ*_1_ (the shape parameter of a Gompertz distribution) are used along with the coefficients in the model to control the distribution of the event times.

The event times *T*_*Eki*_ were specified to be Gompertz distributed with mean *μ*_0_ = 3 and standard deviation *σ*_0_ = 0.5. Using the extreme value distribution (as recommended by Bender et al,^33^ with *γ* ≈ 0.5772 representing Euler's constant), this lead to the parameters controlling the event times distributions to be set to

$$\begin{array}{cc} \theta_{1}= & \;\frac{\pi }{\sqrt{6}\sigma_{0}}=\frac{\pi }{\left(0.5\right)\sqrt{6}}\approx 2.5651\\ \theta_{0}= & \;\log\left(\theta_{1}\exp\left(-\gamma -\mu_{0}\theta_{1}\right)\right)=\log\left(\theta_{1}\exp\left(-\gamma -3\theta_{1}\right)\right)\approx -7.330517. \end{array}$$

A Gompertz distribution has increasing hazard for a positive shape parameter, constant hazard for a shape parameter equal to 0 (equivalent to an exponential distribution), and a decreasing hazard for negative shape parameters. Under the above model, the probability density function of the event times takes form

(5) $$f_{0}\left(t\right)=\kappa \exp\left(\theta_{1}t\right)\exp\left(\frac{\kappa }{\theta_{1}}\left(1-\exp\left(\theta_{1}t\right)\right)\right),\text{where}\;\kappa =\exp\theta_{0}.$$

If the shape parameter is negative, if time is allowed to tend toward infinity, there is a non‐zero probability of living forever. As such, in the function used to simulate event times (available in the aforementioned joineRmeta package), when the Gompertz distribution is employed, event times are simulated under a two step process. First, for each individual *i* within study *k*, the following two conditions are checked (using the realization from the *U*(0, 1) distribution, *U*_*ki*_):

$$\begin{array}{cc} \text{Condition}\;1: & \;\left(\theta_{1}+\alpha^{\left(2\right)}b_{1\mathrm{ki}}^{\left(2\right)}\right)<0\\ \text{Condition}\;2: & \;U_{\mathrm{ki}}<\exp\;\left(\frac{\exp\;\left(\theta_{0}+\alpha^{\left(2\right)}b_{0\mathrm{ki}}^{\left(2\right)}\right)}{\theta_{1}+\alpha^{\left(2\right)}b_{1\mathrm{ki}}^{\left(2\right)}}\right). \end{array}$$

If the conditions are both true, the individual is automatically assigned an event time of infinity; otherwise, their event time is generated under Equation (4).

The censoring times were simulated under an exponential distribution with parameter *λ*_*cens*_. As such, individual censoring times *T*_*Cki*_ are generated using equation

(6) $$T_{\mathrm{Cki}}=\frac{-\log\left(U_{\mathrm{ki}}\right)}{\lambda_{\text{cens}}}.$$

The event rate of the simulated data was controlled through the censoring process. Due to the volume of planned simulations, only datasets with a “low” (∼25%) event rate were generated. A range of censoring parameters were tested to obtain datasets with mean event rate at 25%, leading to setting *λ*_*cens*_ =  exp (−0.426). The survival time for each individual was the minimum of their censoring and event times (*T*_*Ski*_ =  min (*T*_*Eki*_, *T*_*Cki*_)).

All data used in the simulation studies were simulated under the models shown in Equations (2) and (3), although certain parameter values were altered between different sets of simulations. Parameter values in the simulation sets were chosen such that deviations of different methods from the true parameter values would be clearly discernible. A summary of the values used for the different sets of simulations is given in Table 5. All simulation groups utilized the same fixed effect and error term variance values (*β*_10_ = 1, *β*_11_ = 3, *β*_12_ = 2, *β*_21_ = 3, $\sigma_{e}^{2}=0.01$). Additionally, throughout different sets of simulations, the individual level random effects covariance matrix ***D*** remained constant (defined in Table 5). However, the remaining aspects of the datasets (association parameters, number of included studies, level of between study heterogeneity) varied between simulation sets. These aspects are stated in Table 5 and are briefly discussed in the following sections. Throughout, both separate longitudinal or time‐to‐event one stage MA and joint one stage MA were conducted, to compare the two approaches,

**Table 5:** Parameters used when simulating data for simulation investigations

	*Simulation Set 1: Varying*	*Simulation Set 2: Varying Number*	*Simulation Set 3: Varying*
	*Association Parameters*	*of Included Studies*	*Level of Study Heterogeneity*
Number of included studies	5	5, 10, 15	5
Number of individuals within	500	500	500
each study			
Measurement times	0, 0.25, 0.5, 1, 1.5, 2, 2.5, 3, 3.5, 4	0, 0.25, 0.5, 1, 1.5, 2, 2.5, 3, 3.5, 4	0, 0.25, 0.5, 1, 1.5, 2, 2.5, 3, 3.5, 4
Longitudinal fixed effect	β_10_ = 1, β_11_ = 3, β_12_ = 2	β_10_ = 1, β_11_ = 3, β_12_ = 2	β_10_ = 1, β_11_ = 3, β_12_ = 2
parameters (*β*_10_, *β*_11_, *β*_12_)			
Time‐to‐event fixed effect	β_21_ = 3	β_21_ = 3	β_21_ = 3
parameters (*β*_21_)			
Individual level association	α^(2)^ = (0, 0.5, 1)	α^(2)^ = 0.5	α^(2)^ = 0.5
parameter (*α*^(2)^)			
Individual level random effects	$D=\left(\begin{array}{cc} 1 & 0.5\\ 0.5 & 1.5 \end{array}\right)$	$D=\left(\begin{array}{cc} 1 & 0.5\\ 0.5 & 1.5 \end{array}\right)$	$D=\left(\begin{array}{cc} 1 & 0.5\\ 0.5 & 1.5 \end{array}\right)$
covariance matrix (*D*)			
Study level association	α^(3)^ = (0, 0.5, 1)	α^(3)^ = 0.5	α^(3)^ = 0.5
parameter (*α*^(3)^)			
Study level random effects covariance matrix (*A*)			$A_{1}=\left(\begin{array}{cc} 0 & 0\\ 0 & 0 \end{array}\right)$
	$A=\left(\begin{array}{cc} 1 & 0.5\\ 0.5 & 1.5 \end{array}\right)$	$A=\left(\begin{array}{cc} 1 & 0.5\\ 0.5 & 1.5 \end{array}\right)$	$A_{2}=\left(\begin{array}{cc} 1 & 0.5\\ 0.5 & 1.5 \end{array}\right)$
			$A_{3}=\left(\begin{array}{cc} 2 & 1\\ 1 & 3 \end{array}\right)$
Error term variance ( $\sigma_{e}^{2}$)	0.01	0.01	0.01
Parameters controlling event time distribution (*θ*_0_, *θ*_1_)	$\theta_{1}=\frac{\pi }{\left(0.5\right)\sqrt{6}}\;$	$\theta_{1}=\frac{\pi }{\left(0.5\right)\sqrt{6}}\;$	$\theta_{1}=\frac{\pi }{\left(0.5\right)\sqrt{6}}\;$
	*θ*_0_ = log (*θ*_1_ exp (−*γ* − 3*θ*_1_))	*θ*_0_ = log (*θ*_1_ exp (−*γ* − 3*θ*_1_))	*θ*_0_ = log (*θ*_1_ exp (−*γ* − 3*θ*_1_))
Parameter controlling survival	exp(−0.426)	exp(−0.426)	exp(−0.426)
time distribution (*φ*)			

#### 6.1.1 Simulation set 1: Varying levels of association

In practice, the magnitude of the association between the longitudinal and time‐to‐event outcomes at the individual and the study level of the data could impact the performance of the model groups defined in Section 2. Consequently, we performed a simulation investigation to assess the effect of varying magnitudes of association at different levels.

The individual level association parameter *α*^(2)^ and the study level association parameter *α*^(3)^ were permitted to take values 0, 0.5, and 1, giving a total of 9 unique scenarios. The number of included studies in each dataset equalled 5, whereas the study level random effects covariance matrix ***A*** (Table 5) remained constant across scenarios.

#### 6.1.2 Simulation set 2: Varying numbers of studies included in the meta‐analysis

The models introduced in Section 2 that include study level random effects may not reliably estimate the distribution of the study level random effects unless the number of studies included in the meta‐analysis is large. In addition, models including fixed interaction terms between study membership and treatment group may become unwieldy or difficult to estimate as the number of included studies increases. To investigate this, simulations were conducted comparing one‐stage analyses of joint data for datasets containing 5, 10, or 15 studies.

During this set of simulations, the association parameters were held constant across scenarios (with *α*^(2)^ = *α*^(3)^ = 0.5). Additionally, the study level random effects covariance matrix ***A*** (Table 5) remained constant across scenarios.

#### 6.1.3 Simulation set 3: Varying levels of between study heterogeneity

Finally, the level of between study heterogeneity could affect the behavior of the different one‐stage models described in Section 2. As such, the third set of simulations alters the study level random effects covariance matrix ***A*** across different scenarios, to increase or reduce between study heterogeneity. Values taken for ***A***, labeled ***A***_**1**_, ***A***_**2**_, and ***A***_**3**_ are specified in Table 5, representing cases for no between study heterogeneity and, then, two increasing levels of between study heterogeneity.

During this simulation set, across all scenarios, 5 studies were simulated for each dataset, with association parameters held constant across scenarios at *α*^(2)^ = *α*^(3)^ = 0.5.

#### 6.1.4 Models fitted to simulated data

Model groups 0 through 5 (as defined in Table 1) were fitted to each of the datasets simulated for each scenario within each set of simulations. As the data were simulated under a joint model of structure from Model Group 3, the results of fitting examples of Model Group 3 to the data could be expected to provide less biased results than the other model groups.

#### 6.1.5 Reporting of simulation results

For model groups that estimated study specific parameters (the longitudinal treatment effect in model groups 1 and 4, and the time‐to‐event treatment effect in model group 1), overall pooled effects have been reported by combining study specific estimates using methods equivalent to conducting a random effects MA of study level results.^14^, ^35^ Results are reported as the mean estimate produced across studies (SE between simulation estimates) [coverage], where SE is the standard error (the standard deviation) of the produced estimates. As defined by Burton et al,^36^ and using a significance level of *γ* = 0.05, coverage is calculated as the proportion of times the 100(1 − *γ*)% confidence intervals for parameter estimate ${\hat{\beta }}_{v}$, defined by ${\hat{\beta }}_{v}\pm Z_{1-\gamma /2}\mathrm{SE}\left({\hat{\beta }}_{v}\right)$, includes the “true” value of parameter *β* that the data were simulated under (where *Z*_1 − *γ*/2_ ≈ 1.96 for significance level 0.05, and *v* takes values 1 to the total number of simulations performed, here 1000). Where parameters are not estimated for a model group (eg, *α*^(3)^ for model groups not including study level random effects), an NA is printed. The total number of successful model fits are also reported. As the joint models were fitted using the EM algorithm,^22^ separate longitudinal and time‐to‐event models were automatically fitted to determine suitable starting values for the algorithm. Consequently, the number of failed fits was equal for the separate and joint model analyses.

**Table 6:** Simulation group 1 (varying levels of association) results for model groups 0–2. Results reported as mean parameter estimate (SE between simulation estimates) [coverage]

	Association		Number of	Longitudinal Treatment Effect		Time‐to‐Event Treatment Effect		Association Parameters	
	Parameters		Successful Model Fits	(**β**_12_ = 2)		(**β**_21_ = 3)			
	**α**^(2)^	**α**^(3)^		Separate Model	Joint Model	Separate Model	Joint Model	**Joint Model** ***α***^(2)^	**Joint Model** ***α***^(3)^
***Group 0***	0	0	1000	2.02 (0.53) [18.6]	2.02 (0.53) [17.9]	3.02 (0.15) [95.4]	3.02 (0.15) [94.7]	0.00 (0.01) [93.4]	NA
	0	0.5	1000	2.00 (0.56) [17.6]	2.00 (0.56) [16.6]	2.50 (0.35) [24.5]	2.57 (0.32) [31.1]	0.06 (0.04) [18.6]	NA
	0	1	1000	2.00 (0.55) [17.0]	2.00 (0.55) [15.9]	1.93 (0.48) [5.6]	2.04 (0.46) [7.5]	0.10 (0.06) [12.2]	NA
	0.5	0	1000	2.00 (0.56) [17.2]	2.00 (0.56) [16.6]	1.63 (0.11) [0.0]	2.46 (0.26) [14.8]	0.36 (0.07) [5.5]	NA
	0.5	0.5	1000	2.01 (0.55) [18.5]	2.01 (0.55) [17.0]	1.57 (0.18) [0.0]	2.74 (0.29) [43.5]	0.46 (0.04) [49.7]	NA
	0.5	1	1000	1.97 (0.55) [18.9]	1.97 (0.55) [18.0]	1.45 (0.25) [0.0]	2.35 (0.46) [15.5]	0.48 (0.06) [45.9]	NA
	1	0	1000	1.99 (0.55) [18.7]	1.99 (0.55) [17.5]	1.11 (0.09) [0.0]	1.84 (0.38) [2.0]	0.57 (0.15) [0.5]	NA
	1	0.5	1000	2.04 (0.55) [19.7]	2.04 (0.55) [18.5]	1.12 (0.14) [0.0]	2.29 (0.42) [13.2]	0.76 (0.11) [4.7]	NA
	1	1	1000	1.97 (0.55) [18.0]	1.97 (0.55) [16.9]	1.13 (0.21) [0.0]	2.34 (0.55) [18.3]	0.84 (0.12) [22.1]	NA
***Group 1***	0	0	1000	2.02 (0.53) [87.1]	2.02 (0.53) [89.3]	3.04 (0.20) [75.0]	3.04 (0.15) [85.5]	0.00 (0.01) [93.8]	NA
	0	0.5	1000	2.00 (0.55) [87.1]	2.00 (0.55) [88.2]	3.02 (0.54) [57.3]	3.05 (0.27) [84.3]	0.00 (0.01) [93.0]	NA
	0	1	942	2.01 (0.55) [87.9]	2.01 (0.55) [87.9]	2.98 (0.98) [55.1]	3.05 (0.49) [82.9]	0.00 (0.01) [95.2]	NA
	0.5	0	1000	2.00 (0.56) [86.4]	2.00 (0.56) [87.5]	1.64 (0.17) [0.0]	3.03 (0.14) [86.3]	0.51 (0.02) [92.6]	NA
	0.5	0.5	1000	2.01 (0.55) [86.2]	2.01 (0.55) [87.5]	1.69 (0.47) [6.0]	3.04 (0.26) [85.0]	0.51 (0.02) [92.8]	NA
	0.5	1	977	1.97 (0.55) [86.7]	1.97 (0.55) [87.3]	1.79 (0.86) [27.1]	3.02 (0.46) [87.1]	0.51 (0.02) [88.3]	NA
	1	0	1000	1.99 (0.55) [88.8]	1.99 (0.55) [89.3]	1.12 (0.15) [0.0]	3.04 (0.14) [86.7]	1.02 (0.03) [87.8]	NA
	1	0.5	1000	2.04 (0.54) [87.4]	2.04 (0.54) [87.9]	1.12 (0.33) [0.1]	3.06 (0.28) [82.3]	1.02 (0.03) [83.2]	NA
	1	1	998	1.97 (0.54) [87.2]	1.97 (0.54) [87.7]	1.16 (0.59) [3.3]	3.09 (0.52) [82.9]	1.03 (0.04) [75.6]	NA
***Group 2***	0	0	1000	2.02 (0.53) [89.4]	2.02 (0.53) [10.1]	3.03 (0.15) [95.4]	3.04 (0.15) [93.7]	0.00 (0.01) [94.1]	−0.002 (0.24) [98.7]
	0	0.5	1000	2.00 (0.55) [88.3]	2.00 (0.55) [10.1]	3.11 (0.28) [65.1]	3.11 (0.28) [64.7]	0.00 (0.01) [94.2]	0.039 (0.23) [38.1]
	0	1	1000	2.01 (0.55) [87.8]	2.01 (0.55) [9.7]	3.26 (0.51) [32.4]	3.26 (0.51) [33.5]	0.00 (0.02) [94.0]	0.067 (0.26) [8.5]
	0.5	0	999	2.00 (0.56) [87.3]	2.00 (0.56) [10.0]	1.64 (0.11) [0.0]	3.03 (0.14) [93.9]	0.50 (0.02) [94.9]	0.003 (0.24) [99.1]
	0.5	0.5	1000	2.01 (0.55) [87.3]	2.01 (0.55) [11.0]	1.76 (0.21) [0.0]	3.10 (0.27) [62.5]	0.51 (0.02) [93.7]	0.045 (0.55) [32.3]
	0.5	1	1000	1.98 (0.55) [87.9]	1.98 (0.55) [12.0]	2.01 (0.42) [3.4]	3.22 (0.47) [39.3]	0.51 (0.03) [86.7]	0.072 (0.27) [7.8]
	1	0	1000	1.99 (0.55) [89.3]	1.99 (0.55) [11.3]	1.11 (0.09) [0.0]	3.05 (0.14) [92.7]	1.02 (0.03) [90.6]	−0.012 (0.32) [99.2]
	1	0.5	1000	2.04 (0.54) [87.8]	2.04 (0.54) [12.0]	1.18 (0.15) [0.0]	3.10 (0.28) [62.5]	1.01 (0.04) [91.6]	0.026 (0.30) [28.6]
	1	1	999	1.97 (0.54) [87.6]	1.97 (0.54) [10.7]	1.35 (0.30) [0.0]	3.24 (0.52) [35.7]	1.01 (0.04) [88.2]	0.049 (0.24) [5.4]

**Table 7:** Simulation group 1 (varying levels of association) results for model groups 3–5. Results reported as mean parameter estimate (SE between simulation estimates) [coverage]

	Association		Number of	Longitudinal Treatment Effect		Time‐to‐Event Treatment Effect		Association Parameters	
	Parameters		Successful Model Fits	(**β**_12_ = 2)		(**β**_21_ = 3)			
	**α**^(2)^	**α**^(3)^		Separate Model	Joint Model	Separate Model	Joint Model	**Joint Model** ***α***^(2)^	**Joint Model** ***α***^(3)^
***Group 3***	0	0	1000	2.02 (0.53) [89.5]	2.02 (0.53) [11.0]	3.02 (0.15) [95.4]	3.03 (0.15) [94.1]	0.00 (0.01) [94.1]	−0.002 (0.06) [96.4]
	0	0.5	1000	2.00 (0.55) [88.3]	2.00 (0.56) [11.2]	2.50 (0.35) [24.5]	2.90 (0.28) [67.6]	0.00 (0.01) [94.5]	0.429 (0.17) [44.8]
	0	1	1000	2.01 (0.55) [87.9]	2.01 (0.55) [10.3]	1.93 (0.48) [5.6]	2.67 (0.49) [37.2]	0.00 (0.02) [97.3]	0.753 (0.32) [24.7]
	0.5	0	1000	2.00 (0.56) [87.3]	2.00 (0.56) [10.7]	1.63 (0.11) [0.0]	3.02 (0.14) [94.8]	0.50 (0.02) [96.1]	−0.001 (0.06) [96.6]
	0.5	0.5	999	2.01 (0.55) [87.4]	2.01 (0.55) [11.4]	1.57 (0.18) [0.0]	2.88 (0.27) [62.9]	0.48 (0.03) [83.2]	0.427 (0.17) [42.8]
	0.5	1	1000	1.98 (0.55) [87.9]	1.98 (0.55) [12.7]	1.45 (0.25) [0.0]	2.64 (0.44) [35.6]	0.44 (0.04) [45.3]	0.744 (0.31) [23.2]
	1	0	1000	1.99 (0.55) [89.3]	1.99 (0.55) [11.6]	1.11 (0.09) [0.0]	3.04 (0.14) [92.7]	1.01 (0.03) [92.1]	−0.001 (0.05) [96.7]
	1	0.5	1000	2.04 (0.54) [87.8]	2.04 (0.54) [13.4]	1.12 (0.14) [0.0]	2.88 (0.29) [62.7]	0.97 (0.04) [79.8]	0.431 (0.17) [44.9]
	1	1	1000	1.97 (0.54) [87.5]	1.97 (0.54) [11.4]	1.13 (0.21) [0.0]	2.65 (0.49) [33.6]	0.88 (0.07) [31.1]	0.754 (0.32) [23.1]
***Group 4***	0	0	1000	2.01 (0.53) [87.1]	2.01 (0.53) [89.3]	3.02 (0.15) [95.8]	3.02 (0.15) [94.9]	0.00 (0.01) [94.2]	NA
	0	0.5	1000	2.00 (0.55) [87.1]	2.00 (0.55) [87.8]	3.03 (0.28) [72.5]	3.03 (0.28) [71.7]	0.00 (0.01) [94.3]	NA
	0	1	1000	2.01 (0.55) [87.9]	2.01 (0.55) [87.9]	3.08 (0.47) [44.8]	3.08 (0.47) [46.1]	0.00 (0.01) [96.2]	NA
	0.5	0	1000	2.00 (0.56) [86.4]	2.00 (0.56) [87.3]	1.66 (0.11) [0.0]	3.02 (0.14) [94.8]	0.50 (0.02) [96.1]	NA
	0.5	0.5	1000	2.01 (0.54) [86.5]	2.01 (0.54) [87.5]	1.73 (0.20) [0.0]	3.04 (0.26) [69.7]	0.50 (0.02) [95.2]	NA
	0.5	1	1000	1.98 (0.55) [86.9]	1.97 (0.55) [87.8]	1.87 (0.35) [1.1]	3.08 (0.43) [47.1]	0.50 (0.03) [91.7]	NA
	1	0	1000	1.99 (0.55) [88.8]	1.99 (0.55) [89.3]	1.13 (0.09) [0.0]	3.03 (0.14) [93.7]	1.01 (0.03) [93.0]	NA
	1	0.5	1000	2.04 (0.54) [87.4]	2.04 (0.54) [88.0]	1.17 (0.15) [0.0]	3.06 (0.28) [65.6]	1.01 (0.04) [93.8]	NA
	1	1	1000	1.98 (0.54) [87.5]	1.97 (0.54) [87.8]	1.29 (0.26) [0.0]	3.16 (0.49) [42.3]	1.00 (0.04) [88.8]	NA
***Group 5***	0	0	1000	2.00 (0.53) [89.8]	2.00 (0.53) [9.6]	3.02 (0.15) [95.8]	3.03 (0.15) [94.9]	0.00 (0.01) [94.6]	−0.010 (0.25) [99.0]
	0	0.5	1000	2.00 (0.55) [88.3]	2.00 (0.55) [9.5]	3.03 (0.28) [72.5]	3.04 (0.28) [73.2]	0.00 (0.01) [94.3]	0.045 (0.24) [45.8]
	0	1	1000	2.01 (0.55) [87.8]	2.01 (0.55) [9.5]	3.08 (0.47) [44.8]	3.08 (0.47) [46.0]	0.00 (0.01) [95.9]	0.067 (0.27) [12.2]
	0.5	0	999	2.00 (0.56) [87.3]	2.00 (0.56) [10.0]	1.66 (0.11) [0.0]	3.02 (0.14) [94.3]	0.50 (0.02) [95.5]	0.003 (0.26) [98.6]
	0.5	0.5	999	2.04 (0.58) [84.8]	2.04 (0.58) [10.6]	1.74 (0.20) [0.0]	3.04 (0.26) [70.2]	0.50 (0.02) [94.8]	0.030 (0.23) [41.3]
	0.5	1	1000	1.98 (0.55) [87.9]	1.98 (0.55) [11.6]	1.87 (0.35) [1.1]	3.09 (0.43) [48.3]	0.50 (0.03) [91.5]	0.071 (0.29) [10.3]
	1	0	1000	1.99 (0.55) [89.3]	1.99 (0.55) [11.1]	1.13 (0.09) [0.0]	3.04 (0.14) [93.6]	1.01 (0.04) [92.6]	−0.014 (0.39) [99.1]
	1	0.5	1000	2.04 (0.54) [87.8]	2.04 (0.54) [12.3]	1.17 (0.15) [0.0]	3.07 (0.28) [66.2]	1.01 (0.04) [93.0]	0.027 (0.36) [37.4]
	1	1	999	1.97 (0.54) [87.6]	1.97 (0.54) [10.4]	1.29 (0.26) [0.0]	3.16 (0.50) [41.6]	1.00 (0.04) [88.9]	0.047 (0.26) [8.2]

### 6.2 Results of simulation investigations

#### 6.2.1 Results of simulation set 1: Differing levels of association

The results of Simulation Set 1 are presented in Tables 6 and 7. Graphical representations of the mean estimates displayed in Tables 6 and 7 are provided in Supplementary Figures S13–S16, with representations of the point estimates from each simulation given in Supplemental Figures S17–S28. Across the scenarios investigated, most model groups showed a high proportion of successful model fits (99.9% or over). However, model group 1 experienced more failed fits when *α*^(3)^ ≠ 0 (94.2%, 97.7%, and 99.8% model fit success rate).

##### Longitudinal treatment effect (****β****_**12**_)

Throughout Simulation Set 1, the mean pooled longitudinal treatment effect estimate was similar in magnitude between the separate and joint one‐stage analyses. The coverage for model group 0 was poor for both the separate and joint analyses; however, the coverage for the remaining model groups for the separate longitudinal one‐stage MA‐model was consistently high. Conversely, the joint one‐stage MA‐model results displayed high coverage for models that did not include study level random effects, but low coverage across all levels of association for any model group that involved study level random effects. The reason for the comparable mean estimates, but differing coverage, between the separate and joint one‐stage MA‐models, is identifiable through examination of the results from each separate scenario (Supplemental Figures S17–S28). The confidence intervals for *β*_12_ for joint one‐stage models involving study level random effects were quite narrow, leading to poor coverage even though the point estimates are clustered about the “true” value of *β*_12_.

##### Time‐to‐event treatment effect (****β****_**21**_)

For all scenarios investigated in Simulation Set 1, the width of confidence intervals for estimates of *β*_21_ increased for both separate and joint analyses, as *α*^(3)^ increased in magnitude. The results from separate or joint analyses for model group 0 (which ignored between study heterogeneity) were poor when there was non‐zero association.

When individual level association was zero, the estimates produced by the separate analyses for *β*_21_ were close to their “true” value of 3; however, the separate analyses underestimated *β*_21_ when *α*^(2)^ was non‐zero. For the separate analyses, for *α*^(2)^ = 0, coverage for *β*_21_ estimates decreased as study level association increased; however, when *α*^(2)^ ≠ 0, coverage was close to 0.

For the joint analyses, for any model group that accounted for between study heterogeneity in some way (model groups 1–5), the mean estimates were close to the “true” value of *β*_21_ for all model groups; however, model groups 2, 3, and 5 displayed mean estimates diverging from the “true” value of *β*_21_ as the magnitude of the “true” *α*^(3)^ value increased. Coverage was good across all scenarios for model group 1. For the remaining model groups, coverage decreased as the magnitude of the “true” *α*^(3)^ value increased, although coverage was good for joint models from any of model groups 1 to 5 when *α*^(3)^ = 0.

##### Association parameters (****α****^**(2)**^, ****α****^**(3)**^)

The individual level association parameter *α*^(2)^ was poorly estimated by model group 0. However, the estimates of *α*^(2)^ were close to the “true” parameter value for model groups 1, 2, 4, and 5, with good coverage. However, for model group 3, which solely accounted for between study heterogeneity using study level random effects, where the “true” *α*^(2)^ was non‐zero, as the magnitude of *α*^(3)^ increased from zero, the mean parameter estimate decreased in magnitude, with corresponding decrease in coverage.

The estimation of the study level association parameter was poor in model groups 2 and 5, with large coverage values explained by wide confidence intervals (Supplemental Figures S22–S24). Mean estimates of *α*^(3)^ were closer to the “true” values in model group 3, although they were still underestimated. Coverage for all model groups that estimated *α*^(3)^ decreased as the value of the “true” *α*^(3)^ increased.

##### Summary

Under a one‐stage joint model containing a single longitudinal and single time‐to‐event outcome, with association structure sharing both individual and study level random effects (when present), with common association parameter at each level, separate time‐to‐event one‐stage MA‐models appeared to behave poorly when *α*^(2)^ ≠ 0; however, joint one‐stage MA‐models displayed issues when study level random effects were shared between sub‐models.

#### 6.2.2 Results of simulation set 2: Differing numbers of included studies

The results of Simulation Set 2 are presented in Table 8. Graphical representations of the mean estimates displayed in Table 8 are provided in Supplementary Figures S29–S32, with representations of the point estimates from each simulation given in Supplemental Figures S33–S36. The proportion of successful model fits was 99.9% or above for all model groups for all scenarios investigated.

**Table 8:** Simulation group 2 (varying numbers of included studies). Results reported as mean parameter estimate (SE between simulation estimates) [coverage]

	*Number of*	*Number of*	*Longitudinal Treatment Effect*		*Time‐to‐Event Treatment Effect*		*Association Parameters*	
	*Included*	*Successful*	(**β**_12_ = 2)		(**β**_21_ = 3)			
	*Studies*	*Model Fits*	Separate Model	Joint Model	Separate Model	Joint Model	Joint Model	Joint Model
							(**α**^(2)^ = 0.5)	(**α**^(3)^ = 0.5)
***Group 0***	5	1000	2.01 (0.55) [18.5]	2.01 (0.55) [17.0]	1.57 (0.18) [0.0]	2.74 (0.29) [43.5]	0.461 (0.04) [49.7]	NA
	10	1000	2.00 (0.38) [19.5]	2.00 (0.38) [18.8]	1.54 (0.12) [0.0]	2.69 (0.22) [28.4]	0.457 (0.03) [31.3]	NA
	15	1000	2.01 (0.31) [22.6]	2.01 (0.31) [20.1]	1.54 (0.10) [0.0]	2.66 (0.18) [14.6]	0.452 (0.03) [17.5]	NA
***Group 1***	5	1000	2.01 (0.55) [86.2]	2.01 (0.55) [87.5]	1.69 (0.47) [6.0]	3.04 (0.26) [85.0]	0.506 (0.02) [92.8]	NA
	10	1000	2.00 (0.38) [92.2]	2.00 (0.38) [92.6]	1.69 (0.50) [3.2]	3.04 (0.18) [89.6]	0.507 (0.01) [88.0]	NA
	15	1000	2.01 (0.31) [93.9]	2.01 (0.31) [93.5]	1.69 (0.47) [1.9]	3.04 (0.15) [91.7]	0.506 (0.01) [86.0]	NA
***Group 2***	5	1000	2.01 (0.55) [87.3]	2.01 (0.55) [11.0]	1.76 (0.21) [0.0]	3.10 (0.27) [62.5]	0.505 (0.02) [93.7]	0.045 (0.55) [32.3]
	10	1000	2.00 (0.38) [92.2]	2.00 (0.38) [9.8]	1.76 (0.15) [0.0]	3.11 (0.19) [59.1]	0.506 (0.02) [90.4]	0.035 (0.12) [5.3]
	15	1000	2.01 (0.31) [93.3]	2.01 (0.31) [12.2]	1.76 (0.12) [0.0]	3.10 (0.15) [56.4]	0.505 (0.01) [90.6]	0.029 (0.09) [0.5]
***Group 3***	5	999	2.01 (0.55) [87.4]	2.01 (0.55) [11.4]	1.57 (0.18) [0.0]	2.88 (0.27) [62.9]	0.481 (0.03) [83.2]	0.427 (0.17) [42.8]
	10	1000	2.00 (0.38) [92.2]	2.00 (0.38) [12.2]	1.54 (0.12) [0.0]	2.83 (0.19) [52.5]	0.474 (0.02) [60.9]	0.426 (0.10) [38.2]
	15	1000	2.01 (0.31) [93.3]	2.01 (0.31) [13.9]	1.54 (0.10) [0.0]	2.80 (0.16) [39.3]	0.471 (0.02) [39.5]	0.416 (0.08) [29.5]
***Group 4***	5	1000	2.01 (0.54) [86.5]	2.01 (0.54) [87.5]	1.73 (0.20) [0.0]	3.04 (0.26) [69.7]	0.501 (0.02) [95.2]	NA
	10	1000	2.00 (0.38) [92.2]	2.00 (0.38) [92.3]	1.73 (0.14) [0.0]	3.04 (0.18) [69.7]	0.502 (0.02) [94.0]	NA
	15	1000	2.01 (0.31) [93.9]	2.01 (0.31) [93.5]	1.73 (0.11) [0.0]	3.03 (0.15) [70.6]	0.501 (0.01) [94.8]	NA
***Group 5***	5	999	2.04 (0.58) [84.8]	2.04 (0.58) [10.6]	1.74 (0.20) [0.0]	3.04 (0.26) [70.2]	0.501 (0.02) [94.8]	0.030 (0.23) [41.3]
	10	1000	2.00 (0.38) [92.2]	2.00 (0.38) [10.7]	1.73 (0.14) [0.0]	3.04 (0.18) [69.2]	0.503 (0.02) [93.9]	0.038 (0.12) [9.2]
	15	1000	2.01 (0.31) [93.3]	2.01 (0.31) [12.3]	1.73 (0.11) [0.0]	3.03 (0.15) [69.5]	0.501 (0.01) [93.6]	0.032 (0.10) [1.4]

##### Longitudinal treatment effect (****β****_12_)

Across all scenarios investigated, for both the separate and the joint analyses, the mean estimate for the longitudinal treatment effect *β*_12_ was close to the “true” value of 2. Coverage was poor for both the separate and joint analyses for model group 0, which ignores between study heterogeneity. Coverage was consistently good for the separate analyses in the remaining model groups and good for joint models from model groups 1 and 4. However, coverage was poor from joint models for model groups involving study level random effects.

##### Time‐to‐event treatment effect (****β****_21_)

For the time‐to‐event treatment effect *β*_21_, we saw mean estimates from the joint analyses closer to the “true” value of 3 for the joint analyses than the separate analyses. Coverage for the separate analyses was below 6% for all scenarios investigated, whereas coverage for the joint models appeared best for model group 1 (above 85%), followed by model groups 4 and 5 (above 69%). Coverage was noticeably lower for model group 0, which ignored between study heterogeneity, and coverage decreased for model groups 2 and 3 as the number of included studies increased.

##### Association parameters (****α****^(2)^, ****α****^(3)^)

The mean estimate for the individual level association was close to the “true” value of 0.5 for model groups 1–5, with slightly worse estimates from model group 0. Coverage was good for model groups 1, 2, 4, and 5. However, coverage decreased with increasing the number of studies for model groups 0 and 3.

Study level association was poorly estimated in model groups 2 and 5, with estimates closer to the “true” value of 0.5 for model group 3. However, coverage was consistently poor and decreased with increasing the number of included studies.

##### Summary

Under a one‐stage joint model containing a single longitudinal and single time‐to‐event outcome, with association structure sharing both individual and study level random effects, with common association parameter at each level, there appeared to be little benefit of increasing the number of included studies. However, this result may not hold for other association structures, eg, just sharing individual level random effects between studies.

#### 6.2.3 Results of simulation set 3: Differing levels of between study heterogeneity

The results of Simulation Set 3 are presented in Table 9. Graphical representations of the mean estimates displayed in Table 9 are provided in Supplementary Figures S37–S40, with representations of the point estimates from each simulation given in Supplemental Figures S41–S44. There were issues with model fitting for a large proportion of simulations for model groups involving study level random effects when there was no between study heterogeneity (***A*** = ***A***_**1**_); otherwise, the proportion of successful fits was 99.8% or over.

**Table 9:** Simulation group 3 (varying levels of between study heterogeneity). Results reported as mean parameter estimate (SE between simulation estimates) [coverage]. Matrices **A**_**1**_, **A**_**2**_, and **A**_**3**_ represent increasing study heterogeneity (exact matrix definitions available in Table 5)

	*Study Level*	*Number of*	*Longitudinal Treatment Effect*		*Time‐to‐Event Treatment Effect*		*Association Parameters*	
	*Covariance*	*Successful*	(**β**_12_ = 2)		(**β**_21_ = 3)			
	*Matrix*	*Model Fits*	Separate Model	Joint Model	Separate Model	Joint Model	Joint Model	Joint Model
							(**α**^(2)^ = 0.5)	(**α**^(3)^ = 0.5)
***Group 0***	*A* = *A*_1_	1000	2.00 (0.04) [95.2]	2.00 (0.04) [94.8]	1.63 (0.11) [0.0]	3.01 (0.14) [93.7]	0.502 (0.02) [93.4]	NA
	*A* = *A*_2_	1000	2.01 (0.55) [18.5]	2.01 (0.55) [17.0]	1.57 (0.18) [0.0]	2.74 (0.29) [43.5]	0.461 (0.04) [49.7]	NA
	*A* = *A*_3_	1000	1.99 (0.79) [17.3]	1.99 (0.79) [16.1]	1.52 (0.22) [0.0]	2.57 (0.40) [28.0]	0.435 (0.06) [32.8]	NA
***Group 1***	*A* = *A*_1_	1000	2.00 (0.04) [97.2]	2.00 (0.04) [96.6]	1.65 (0.18) [0.0]	3.03 (0.14) [87.5]	0.506 (0.02) [91.7]	NA
	*A* = *A*_2_	1000	2.01 (0.55) [86.2]	2.01 (0.55) [87.5]	1.69 (0.47) [6.0]	3.04 (0.26) [85.0]	0.506 (0.02) [92.8]	NA
	*A* = *A*_3_	998	2.00 (0.79) [88.4]	1.99 (0.79) [88.4]	1.69 (0.63) [13.3]	3.05 (0.35) [85.9]	0.508 (0.02) [90.5]	NA
***Group 2***	*A* = *A*_1_	76	2.01 (0.04) [100.0]	2.00 (0.04) [97.4]	1.64 (0.12) [0.0]	3.05 (0.15) [93.4]	0.512 (0.02) [93.4]	−0.377 (8.86) [100.0]
	*A* = *A*_2_	1000	2.01 (0.55) [87.3]	2.01 (0.55) [11.0]	1.76 (0.21) [0.0]	3.10 (0.27) [62.5]	0.505 (0.02) [93.7]	0.045 (0.55) [32.3]
	*A* = *A*_3_	1000	2.00 (0.79) [88.5]	1.99 (0.79) [7.9]	1.85 (0.29) [0.0]	3.16 (0.35) [50.7]	0.508 (0.02) [90.7]	0.027 (0.16) [15.2]
***Group 3***	*A* = *A*_1_	201	2.00 (0.03) [97.0]	2.00 (0.03) [45.3]	1.63 (0.11) [0.0]	3.02 (0.14) [44.3]	0.505 (0.02) [44.3]	0.380 (2.64) [47.3]
	*A* = *A*_2_	999	2.01 (0.55) [87.4]	2.01 (0.55) [11.4]	1.57 (0.18) [0.0]	2.88 (0.27) [62.9]	0.481 (0.03) [83.2]	0.427 (0.17) [42.8]
	*A* = *A*_3_	1000	2.00 (0.79) [88.5]	2.00 (0.79) [8.6]	1.52 (0.22) [0.0]	2.78 (0.36) [47.3]	0.464 (0.03) [65.1]	0.403 (0.16) [31.8]
***Group 4***	*A* = *A*_1_	1000	2.00 (0.04) [97.2]	2.00 (0.04) [96.7]	1.67 (0.11) [0.0]	3.01 (0.14) [94.7]	0.502 (0.02) [95.3]	NA
	A = A_2_	1000	2.01 (0.54) [86.5]	2.01 (0.54) [87.5]	1.73 (0.20) [0.0]	3.04 (0.26) [69.7]	0.501 (0.02) [95.2]	NA
	*A* = *A*_3_	1000	2.00 (0.79) [88.4]	1.99 (0.79) [88.6]	1.78 (0.26) [0.0]	3.06 (0.33) [56.9]	0.502 (0.02) [93.6]	NA
***Group 5***	*A* = *A*_1_	53	2.00 (0.04) [100.0]	2.00 (0.04) [100.0]	1.66 (0.12) [0.0]	3.04 (0.17) [94.3]	0.509 (0.02) [96.2]	−1.343 (6.03) [100.0]
	*A* = *A*_2_	999	2.04 (0.58) [84.8]	2.04 (0.58) [10.6]	1.74 (0.20) [0.0]	3.04 (0.26) [70.2]	0.501 (0.02) [94.8]	0.030 (0.23) [41.3]
	*A* = *A*_3_	1000	2.00 (0.79) [88.5]	1.99 (0.79) [7.8]	1.78 (0.26) [0.0]	3.07 (0.33) [56.4]	0.503 (0.02) [93.5]	0.030 (0.17) [19.8]

##### Longitudinal treatment effect (****β****_12_)

Across scenarios investigated, the mean estimated longitudinal treatment effect produced by both the separate and joint one‐stage MA‐model were close to the “true” parameter values. Coverage of estimates produced by model group 0 was good from both the separate and the joint one‐stage MA‐models when no between study heterogeneity existed; however, coverage decreased as between study heterogeneity increased. For the remaining model groups, coverage was consistently good for the separate analyses, but joint analyses involving study level random effects displayed decreasing coverage as between study heterogeneity increased.

##### Time‐to‐event treatment effect (****β****_21_)

Throughout the scenarios investigated, the time‐to‐event treatment effect was consistently underestimated by the separate analyses compared to the joint (which displayed estimates closer to the “true” value of the parameters). Models involving study level random effects showed estimates diverging slightly from the “true” value as between study heterogeneity increased. Coverage was consistently good for model group 1; however, the remaining model groups displayed decreasing coverage as between study heterogeneity increased.

##### Association parameters (****α****^(2)^, ****α****^(3)^)

The mean estimate for individual level association *α*^(2)^ was good for model groups 1, 2, 4, and 5, with corresponding high coverage. However, model groups 0 and 3 showed mean estimates increasingly below the true value, with corresponding decreasing coverage as between study heterogeneity increased.

Mean estimates for study level association *α*^(3)^ were poor for model groups 2 and 5, and closer to the true value for model group 3. Coverage was good for model groups 2 and 5 for the case of no between study heterogeneity, and decreased as between study heterogeneity increased. However, examination of Supplemental Figure S44 indicates that wide confidence intervals explained the higher coverage at no between study heterogeneity, with the width of confidence intervals decreasing as between study heterogeneity increases. Coverage was relatively constant but not good for model group 3 across examined levels of between study heterogeneity.

##### Summary

Under a one‐stage joint model containing a single longitudinal and single time‐to‐event outcome, with association structure sharing both individual and study level random effects, with common association parameter at each level, model group 1 appeared to be the most consistently reliable modeling option. However, as noted earlier, this result may not hold for other joint model specifications.

## 7 DISCUSSION

In this research, we have presented and investigated a variety of models for use when analyzing multi‐study joint longitudinal and time‐to‐event data. Analyses of single study joint datasets are increasing.^8^ Ensuring availability of appropriate methods for the meta‐analysis of such data is vital, in order to maximize use of available data and better inform healthcare decisions.

We have examined a range of the possible modeling options; however, other combinations of the approaches discussed here to account for between study heterogeneity are also possible. Each of the model groups examined presents a range of advantages and disadvantages. Models that use fixed effects to account for between study heterogeneity estimate *K* − 1 parameters for each term involving study membership (one for each study apart from the reference study). As such, results may not be generalizable to external studies, and the number of parameters estimated quickly increases as the number of studies included in the meta‐analysis increases. However, such methods do allow calculation of effect sizes within each study (although this is not a primary aim of meta‐analyses).

Conversely, use of study level random effects accounts for between study heterogeneity, but study specific effect estimates are not generally automatically provided (unless the estimates of the random effects can be extracted from models fitted). However, this should not be an issue, as meta‐analyses aim to pool data rather than provide study specific estimates. The number of parameters to be fitted due to study level random effects does not increase as the number of included studies increases. However, the distribution of the random effects may be poorly estimated unless a large number of studies are included in the meta‐analysis.

Additionally, model groups with a common baseline hazard across studies assume proportional hazards across all studies included in the meta‐analysis. However, model groups that stratify the baseline hazard by study assume proportional hazards within but not across studies. This may be a more reasonable assumption, especially if the demographics of the studies differ.

The simulation investigation displayed poor performance for models that ignored any between study heterogeneity present in the data. Consequently, it is clear that accounting for any between study heterogeneity present in multi‐study joint data is vital. The most consistently well‐performing model group was model group 1, which accounted for between study heterogeneity using fixed study membership and interaction between study membership and treatment assignment in both sub‐models. The remaining model groups for the joint analyses showed issues under various scenarios. As the coverage was good for separate models for any model group that accounted for between study heterogeneity, the poor coverage in the joint analyses for model groups 2, 3, and 5 may be due to the dual use of the study level random effects to account for between study heterogeneity and account for study level behavior in the link between the longitudinal and time‐to‐event outcomes. It may be that this dual use is not possible, unless an unrealistically large number of studies are included in the meta‐analysis.

While point estimates were similar in magnitude between the separate and joint analyses for the longitudinal treatment effect, we note bias in the estimates of the time‐to‐event treatment effect from separate analyses where a non‐zero association between the longitudinal and time‐to‐event outcomes is present. This behavior has previously been noted in single study cases by Guo and Carlin,^18^ and in two‐stage joint MA analyses by Sudell et al,^16^ our research confirms that this issue persists for one‐stage analyses. This behavior may be comparable to the established situation where omission of covariates from Cox models causes bias in estimated effect parameters.^37^, ^38^, ^39^ The *W*_2*ki*_(*t*) term is included in the joint time‐to‐event sub‐model but is not present in the separate time‐to‐event sub‐model. Where association is present (ie, where *α*^(2)^ ≠ 0 or *α*^(3)^ ≠ 0), the joint analyses model risk of an event associated with the longitudinal outcome through the *W*_2*ki*_(*t*) term. This term (which has an effect on risk of an event when association is present) is not included in the separate time‐to‐event model, giving a possible explanation for the observed biased treatment effect estimates. As noted in the work of Sudell et al,^16^ similar behavior was not observed between the separate and joint longitudinal analyses as the model specifications for the longitudinal trajectory are identical in both cases. As such, it is recommended that joint one‐stage MA‐models are used in place of separate time‐to‐event one‐stage MA‐models where significant association exists. This can be assessed prior to analyses through plotting of the longitudinal trajectories paneled by event type^16^; differences between the trajectories between those censored and experiencing an event can indicate presence of such an association.

The models investigated utilized an unspecified baseline hazard in the time‐to‐event sub‐model. Hseih et al^40^ noted that when unspecified baseline hazards are used in a joint model, standard errors should be obtained through bootstrapping procedures to avoid their underestimation. As such, the time commitment to perform bootstrapping procedures on large meta‐datasets was considerable. Performing bootstrapping procedures on a standard computing environment took several days for the real dataset. Consequently, bootstraps were performed in parallel using the University of Liverpool's HTCondor system (see the work of Litzkow et al,^41^ https://research.cs.wisc.edu/htcondor/, and http://condor.liv.ac.uk/), which was also used to run the simulations, with the results compiled using purpose written code rather than relying on single computer bootstrapping procedures. Researchers using large datasets without coding experience or access to such computer systems may experience issues conducting large scale joint data meta‐analyses.

In our clinical example, we assume common association parameters across treatment groups. However, in reality, the association between the longitudinal blood pressure and risk of an event could differ between those assigned to any treatment for hypertension versus those assigned to placebo, no treatment, or usual care.^42^ In single study cases, association structures involving interactions between baseline covariates and the association parameters have been presented^2^, ^4^; however, this association structure has not yet been investigated in meta‐analytic joint models.

The research presented here prompts a range of future areas of research. Investigation of one‐stage joint MA‐models with varying association structures, including sharing only individual level random effects or sharing the current value of the longitudinal trajectory, is vital. Additionally, it is vital to investigate alternative modeling options, such as alternative baseline hazard specifications, which could reduce model fitting times by removing the necessity of bootstrapping. In addition, in our simulation study, we assumed common longitudinal measurement schedules across the included studies, identical numbers of individuals recruited to each study, and common association parameter across studies. Further simulation investigations varying these aspects could provide additional useful information for future joint data meta‐analyses.

In conclusion, this research indicates the benefit of the one‐stage meta‐analysis of joint longitudinal and time‐to‐event data where significant association exists between the longitudinal and time‐to‐event outcomes. Given the current research, it is recommended that analyses do not rely on models that share study level random effects between sub‐models. Further research into one‐stage joint MA‐models is required.

## Supporting information

SIM_7961‐Supp‐0001‐Supplemental Material ‐ Results.docx

SIM_7961‐Supp‐0002‐Supplemental Material ‐ Maximum Likelihood Estimators.docx

## References

[1] Wulfsohn MS , Tsiatis AA . A joint model for survival and longitudinal data measured with error. Biometrics. 1997;53(1):330‐339. 9147598

[2] Gould AL , Boye ME , Crowther MJ , et al. Joint modeling of survival and longitudinal non‐survival data: current methods and issues. Report of the DIA Bayesian joint modeling working group. Statist Med. 2015;34(14):2181‐2195. 10.1002/sim.6141 PMC4677775 24634327

[3] Henderson R , Diggle P , Dobson A . Joint modelling of longitudinal measurements and event time data. Biostatistics. 2000;1(4):465‐480. 12933568 10.1093/biostatistics/1.4.465

[4] Rizopoulos D . Joint Models for Longitudinal and Time‐to‐Event Data With Applications in R. 1st ed. Boca Raton, FL: CRC Press; 2012.

[5] Tsiatis AA , Davidian M . Joint modeling of longitudinal and time‐to‐event data: an overview. Stat Sin. 2004;14(3):809‐834.

[6] Powney M , Williamson P , Kirkham J , Kolamunnage‐Dona R . A review of the handling of missing longitudinal outcome data in clinical trials. Trials. 2014;15:237. 24947664 10.1186/1745-6215-15-237 PMC4087243

[7] Powell CV , Kolamunnage‐Dona R , Lowe J , et al. MAGNEsium Trial in Children (MAGNETIC): a randomised, placebo‐controlled trial and economic evaluation of nebulised magnesium sulphate in acute severe asthma in children. Health Technol Assess. 2013;17(45):v‐vi. 10.3310/hta17450 PMC4781380 24144222

[8] Sudell M , Kolamunnage‐Dona R , Tudur Smith C . Joint models for longitudinal and time‐to‐event data: a review of reporting quality with a view to meta‐analysis. BMC Med Res Methodol. 2016;16(1):168. 27919221 10.1186/s12874-016-0272-6 PMC5139124

[9] Jacoby A , Sudell M , Tudur Smith CT , Crossley J , Marson AG , Baker GA . Quality‐of‐life outcomes of initiating treatment with standard and newer antiepileptic drugs in adults with new‐onset epilepsy: findings from the SANAD trial. Epilepsia. 2015;56(3):460‐472. 25630353 10.1111/epi.12913

[10] Kolamunnage‐Dona R , Powell C , Williamson PR . Modelling variable dropout in randomised controlled trials with longitudinal outcomes: application to the MAGNETIC study. Trials. 2016;17:1‐9. 27125779 10.1186/s13063-016-1342-0 PMC4849065

[11] Lloyd‐Williams M , Payne S , Reeve J , Dona RK . Thoughts of self‐harm and depression as prognostic factors in palliative care patients. J Affect Disord. 2014;166:324‐329. 25012448 10.1016/j.jad.2014.05.029

[12] Kovanda LL , Kolamunnage‐Dona R , Neely M , Maertens J , Lee M , Hope WW . Pharmacodynamics of Isavuconazole for invasive mould disease: role of Galactomannan for real‐time monitoring of therapeutic response. Clin Infect Dis. 2017;64(11):1557‐1563. 28472247 10.1093/cid/cix198 PMC5434340

[13] Glass GV . Primary, secondary, and meta‐analysis of research. Educ Res. 1976;5(10):3‐8.

[14] Whitehead A . Meta‐Analysis of Controlled Clinical Trials. Chichester, UK: John Wiley & Sons; 2002.

[15] Higgins J, Green S, eds. Cochrane Handbook for Systematic Reviews of Interventions Version 5.1.0. London, UK: The Cochrane Collaboration; 2011 www.cochrane-handbook.org. Updated March 2011.

[16] Sudell M , Tudur Smith C , Gueyffier F , Kolamunnage‐Dona R . Investigation of 2‐stage meta‐analysis methods for joint longitudinal and time‐to‐event data through simulation and real data application. Statist Med. 2017;37(8):1227‐1244. 10.1002/sim.7585 PMC5887954 29250814

[17] Powell C , Kolamunnage‐Dona R , Lowe J , et al. Magnesium sulphate in acute severe asthma in children (MAGNETIC): a randomised, placebo‐controlled trial. Lancet Respir Med. 2013;1(4):301‐308. 24429155 10.1016/S2213-2600(13)70037-7

[18] Guo X , Carlin BP . Separate and joint modeling of longitudinal and event time data using standard computer packages. Am Stat. 2004;58(1):16‐24.

[19] Yu MG , Law NJ , Taylor JMG , Sandler HM . Joint longitudinal‐survival‐cure models and their application to prostate cancer. Stat Sin. 2004;14(3):835‐862.

[20] Brombin C , Di Serio C , Rancoita PMV . Joint modeling of HIV data in multicenter observational studies: a comparison among different approaches. Stat Methods Med Res. 2016;25(6):2472‐2487. 24671658 10.1177/0962280214526192

[21] Liu L , Ma JZ , O'Quigley J . Joint analysis of multi‐level repeated measures data and survival: an application to the end stage renal disease (ESRD) data. Statist Med. 2008;27(27):5679‐5691. 10.1002/sim.3392 18693300

[22] Dempster AP , Laird NM , Rubin DB . Maximum likelihood from incomplete data via the EM algorithm. J R Stat Soc Ser B Methodol. 1977;39(1):1‐38.

[23] Rizopoulos D . Fast fitting of joint models for longitudinal and event time data using a pseudo‐adaptive Gaussian quadrature rule. Comput Stat Data Anal. 2012;56(3):491‐501.

[24] R Core Team . R: a language and environment for statistical computing [computer program]. Vienna, Austria: R Foundation for Statistical Computing; 2015.

[25] Philipson P , Sousa I , Diggle P , Williamson P , Kolamunnage‐Dona R , Henderson R , Hickey G . joineR: Joint Modelling of Repeated Measurements and Time-to-Event Data. R package version 1.2.4. 2018 https://github.com/graemeleehickey/joineR/

[26] Gueyffier F , Boutitie F , Boissel JP , et al. INDANA: a meta‐analysis on individual patient data in hypertension. Protocol and preliminary results. Therapie. 1995;50:353‐362. 7482389

[27] Amery A , Birkenhäger W , Brixko P , et al. Mortality and morbidity results from the European working party on high blood pressure in the elderly trial. Lancet. 1985;325(8442):1349‐1354. 10.1016/s0140-6736(85)91783-0 2861311

[28] Coope J , Warrender TS . Randomised trial of treatment of hypertension in elderly patients in primary care. BMJ. 1986;293:1145‐1151. 3094811 10.1136/bmj.293.6555.1145 PMC1341855

[29] Dalhöf B , Lindholm LH , Hansson L , Scherstén B , Ekbom T , Wester PO . Morbidity and mortality in the Swedish trial in old patients with hypertension (STOP‐hypertension). Lancet. 1991;338(8778):1281‐1285. 1682683 10.1016/0140-6736(91)92589-t

[30] SHEP Cooperative Research Group . Prevention of stroke by antihypertensive drug treatment in older persons with isolated systolic hypertension: final results of the Systolic Hypertension in the Elderly Program (SHEP). JAMA. 1991;265:3255‐3264. 2046107

[31] Medical Research Council Working Party . MRC trial of treatment of mild hypertension: principal results. BMJ. 1985;291:97‐104. 2861880 10.1136/bmj.291.6488.97 PMC1416260

[32] MRC Working Party . Medical research council trial of treatment of hypertension in older adults: principal results. BMJ. 1992;304:405‐412. 1445513 10.1136/bmj.304.6824.405 PMC1995577

[33] Bender R , Augustin T , Blettner M . Generating survival times to simulate cox proportional hazards models. Statist Med. 2005;24:1713‐1723. 10.1002/sim.2059 15724232

[34] Austin PC . Generating survival times to simulate Cox proportional hazards models with time‐varying covariates. Statist Med. 2012;31:3946‐3958. 10.1002/sim.5452 PMC3546387 22763916

[35] DerSimonian R , Laird N . Meta‐analysis in clinical trials. Control Clin Trials. 1986;7(3):177‐188. 3802833 10.1016/0197-2456(86)90046-2

[36] Burton A , Altman DG , Royston P , Holder RL . The design of simulation studies in medical statistics. Statist Med. 2006;25:4279‐4292. 10.1002/sim.2673 16947139

[37] Bretagnolle J , Huber‐Carol C . Effects of omitting covariates in Cox's model for survival data. Scand J Stat. 1988;15(2):125‐138.

[38] Abrahamowicz M , du Berger R , Krewski D , et al. Bias due to aggregation of individual covariates in the Cox regression model. Am J Epidemiol. 2004;160(7):696‐706. 15383414 10.1093/aje/kwh266

[39] Schmoor C , Schumacher M . Effects of covariate omission and categorization when analysing randomized trials with the Cox model. Statist Med. 1997;16:225‐237. 10.1002/(sici)1097-0258(19970215)16:3<225::aid-sim482>3.0.co;2-c 9004394

[40] Hsieh F , Tseng Y‐K , Wang J‐L . Joint modeling of survival and longitudinal data: likelihood approach revisited. Biometrics. 2006;62(4):1037‐1043. 17156277 10.1111/j.1541-0420.2006.00570.x

[41] Litzkow MJ , Livny M , Mutka MW . Condor ‐ a hunter of idle workstations. Paper presented at: 8th International Conference on Distributed Computing Systems; 1988; San Jose, CA.

[42] Ekbom T , Dahlöf B , Hansson L , et al. The stroke preventive effect in elderly hypertensives cannot fully be explained by the reduction in office blood pressure‐insights from the Swedish trial in old patients with hypertension (STOP‐hypertension). Blood Press. 1992;1(3):168‐172. 1345050 10.3109/08037059209077513

[43] Perry MH Jr , Smith MW , McDonald RH , et al. Morbidity and mortality in the Systolic Hypertension in the Elderly Program (SHEP) pilot study. Stroke. 1989;20:4‐13. 2911834 10.1161/01.str.20.1.4

