# Supplementary material for: Investigation of one‐stage meta‐analysis methods for joint longitudinal and time‐to‐event data through simulation and real data application
Source: Stat Med. 2018 Sep 12;38(2):247–68. doi: 10.1002/sim.7961 (PMC6492085; doi:10.1002/sim.7961)
Supplement: Supplementary file 1 — SIM_7961‐Supp‐0001‐Supplemental Material ‐ Results.docx [file SIM-38-247-s001.docx]

Supplementary Information

# Plots of Trajectories panelled by event type

## SBP and time to death


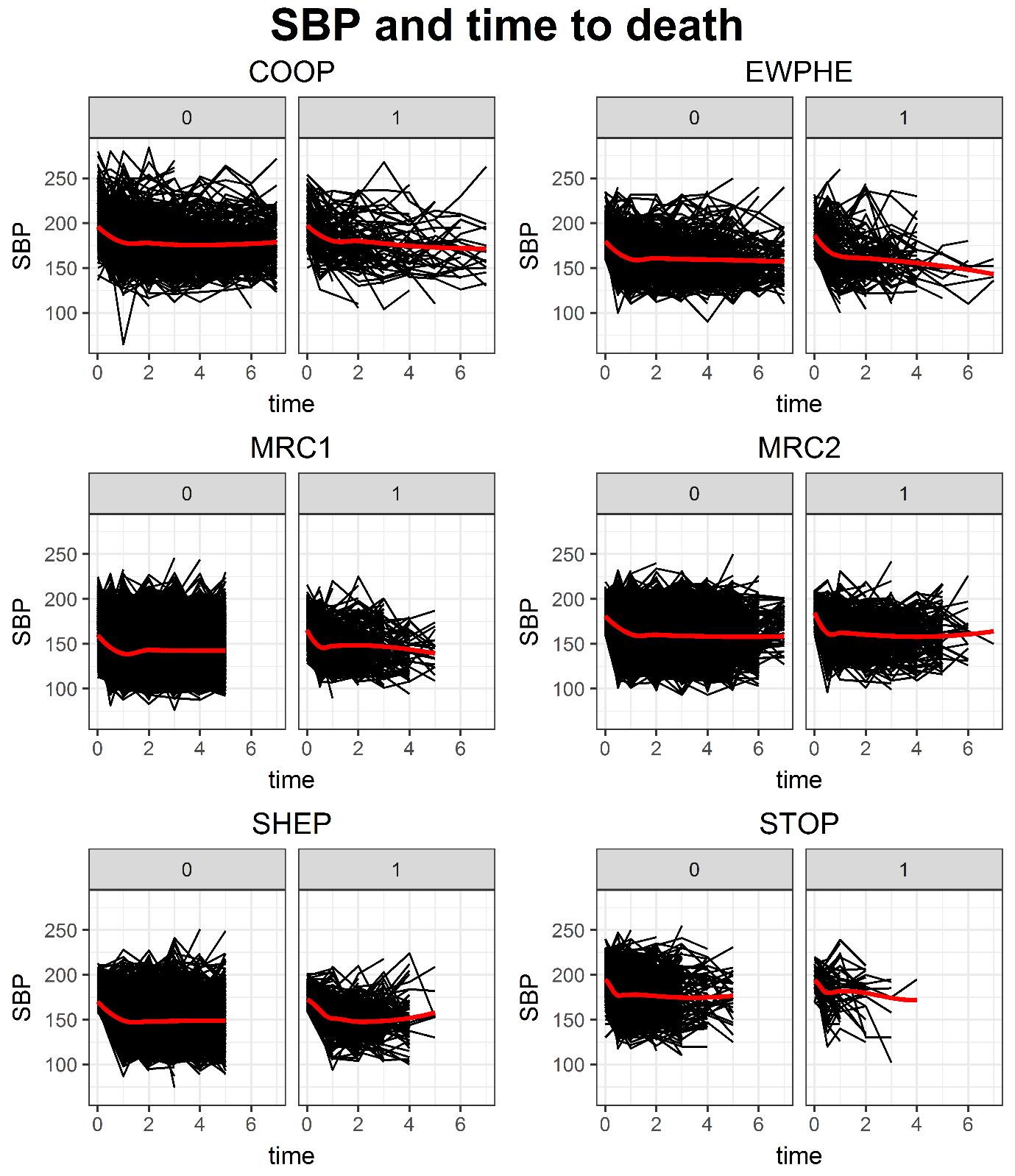


Supplemental Figure S1: Longitudinal trajectory plots with mean trajectory smoother (red line) for SBP and time to death data, with 0 indicating a censoring and 1 indicating an event was experienced.

## SBP and time to MI


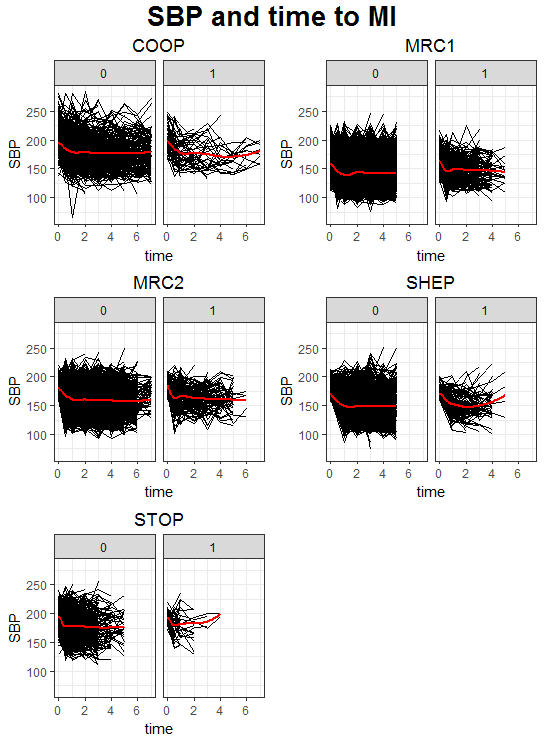


Supplemental Figure S2: Longitudinal trajectory plots with mean trajectory smoother (red line) for SBP and time to myocardial infarction (MI) data, with 0 indicating a censoring and 1 indicating an event was experienced.

## SBP and time to stroke


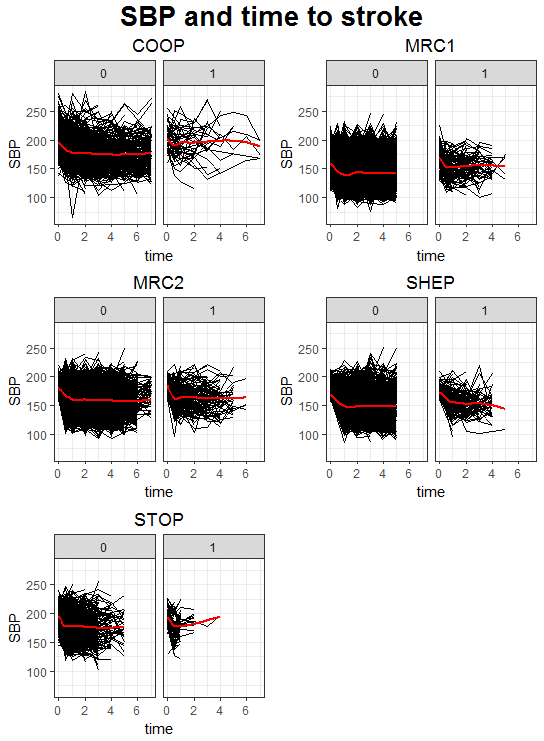


Supplemental Figure S3: Longitudinal trajectory plots with mean trajectory smoother (red line) for SBP and time to stroke data, with 0 indicating a censoring and 1 indicating an event was experienced.

# Table of number of longitudinal measurements at each time point

## SBP and time to death

| SBP and time to death | | Time (years) | | | | | | | | | Number of measurement times recorded in study |
| --- | --- | --- | --- | --- | --- | --- | --- | --- | --- | --- | --- |
|  |  | 0 | 0.5 | 1 | 2 | 3 | 4 | 5 | 6 | 7 |  |
| Study | COOP | 884 | 760 | 785 | 722 | 514 | 329 | 267 | 200 | 160 | 9 |
|  | EWPHE | 840 | 749 | 653 | 509 | 383 | 297 | 213 | 118 | 63 | 9 |
|  | MRC1 | 17354 | 16525 | 16343 | 15308 | 14611 | 12584 | 8353 | 0 | 0 | 7 |
|  | MRC2 | 4394 | 4182 | 4100 | 3765 | 3490 | 3223 | 2596 | 655 | 52 | 9 |
|  | SHEP | 4736 | 0 | 4243 | 4091 | 3938 | 2644 | 1164 | 0 | 0 | 6 |
|  | STOP | 1612 | 1520 | 1440 | 798 | 311 | 67 | 29 | 0 | 0 | 7 |

Supplemental Table S1: Number of longitudinal measurements available at each time point by study for the analysis of SBP and time to death

## SBP and time to MI

| SBP and time to MI | | Time (years) | | | | | | | | | Number of measurement times recorded in study |
| --- | --- | --- | --- | --- | --- | --- | --- | --- | --- | --- | --- |
|  |  | 0 | 0.5 | 1 | 2 | 3 | 4 | 5 | 6 | 7 |  |
| Study | COOP | 884 | 759 | 782 | 713 | 506 | 319 | 260 | 195 | 155 | 9 |
|  | MRC1 | 17354 | 16512 | 16309 | 15253 | 14520 | 12478 | 8273 | 0 | 0 | 7 |
|  | MRC2 | 4394 | 4176 | 4086 | 3739 | 3465 | 3183 | 2564 | 644 | 52 | 9 |
|  | SHEP | 4728 | 0 | 4220 | 4051 | 3877 | 2592 | 1141 | 0 | 0 | 6 |
|  | STOP | 1612 | 1518 | 1433 | 784 | 299 | 65 | 28 | 0 | 0 | 7 |

Supplemental Table S2: Number of longitudinal measurements available at each time point by study for the analysis of SBP and time to myocardial infarction (MI)

## SBP and time to stroke

| SBP and time to stroke | | Time (years) | | | | | | | | | Number of measurement times recorded in study |
| --- | --- | --- | --- | --- | --- | --- | --- | --- | --- | --- | --- |
|  |  | 0 | 0.5 | 1 | 2 | 3 | 4 | 5 | 6 | 7 |  |
| Study | COOP | 884 | 754 | 777 | 709 | 496 | 316 | 251 | 187 | 153 | 9 |
|  | MRC1 | 17354 | 16521 | 16325 | 15282 | 14572 | 12542 | 8318 | 0 | 0 | 7 |
|  | MRC2 | 4394 | 4172 | 4080 | 3737 | 3451 | 3177 | 2547 | 642 | 51 | 9 |
|  | SHEP | 4736 | 0 | 4206 | 4005 | 3834 | 2555 | 1105 | 0 | 0 | 6 |
|  | STOP | 1612 | 1515 | 1418 | 767 | 294 | 66 | 29 | 0 | 0 | 7 |

Supplemental Table S3: Number of longitudinal measurements available at each time point by study for the analysis of SBP and time to stroke

# Table of model specifications for analysis of INDANA dataset

| ***Model Group*** | ***Model component*** | ***Equation*** |
| --- | --- | --- |
| **0** | **Longitudinal Sub-Model** | $Y_{kij}=\beta_{10}+\beta_{11}t_{kij}+\beta_{12}treat_{ki}+\beta_{13}\exp\left( -3*t_{kij} \right)$ $+b_{0ki}^{(2)}+b_{1ki}^{(2)}t_{kij}+\varepsilon_{kij}$ |
|  | **Time-to-event Sub-Model** | $\lambda_{ki}\left( t \right)= \lambda_{0}\left( t \right)\exp(\beta_{21}treat_{ki}+W_{2ki}\left( t \right))$ |
|  | **Association Structure** | $W_{2ki}\left( t \right)=\alpha^{\left( 2 \right)}(b_{0ki}^{\left( 2 \right)}+b_{1ki}^{\left( 2 \right)}s_{ki})$ |
| **1** | **Longitudinal Sub-Model** | $Y_{kij}=\beta_{10}+\beta_{11}t_{kij}+\beta_{12}treat_{ki}$ $+\beta_{13}{study}_{ki}+\beta_{14}treat_{ki}*{study}_{ki}$ $+\beta_{15}\exp\left( -3*t_{kij} \right)$ $+b_{0ki}^{(2)}+b_{1ki}^{(2)}t_{kij}+\varepsilon_{kij}$ |
|  | **Time-to-event Sub-Model** | $\lambda_{ki}\left( t \right)= \lambda_{0}\left( t \right)\exp(\beta_{21}treat_{ki}+\beta_{22}{study}_{ki}+\beta_{23}treat_{ki}*{study}_{ki}+W_{2ki}\left( t \right))$ |
|  | **Association Structure** | $W_{2ki}\left( t \right)=\alpha^{\left( 2 \right)}(b_{0ki}^{\left( 2 \right)}+b_{1ki}^{\left( 2 \right)}s_{ki})$ |
| **2** | **Longitudinal Sub-Model** | $Y_{kij}=\beta_{10}+\beta_{11}t_{kij}+\beta_{12}treat_{ki}$ $+\beta_{13}{study}_{ki}+\beta_{14}\exp\left( -3*t_{kij} \right)$ $+b_{0ki}^{\left( 2 \right)}+b_{1ki}^{\left( 2 \right)}t_{kij}$ $+b_{1k}^{(3)}treat_{ki}+\varepsilon_{kij}$ |
|  | **Time-to-event Sub-Model** | $\lambda_{ki}\left( t \right)= \lambda_{0}\left( t \right)\exp(\beta_{21}treat_{ki}+\beta_{22}{study}_{ki}+W_{2ki}\left( t \right))$ |
|  | **Association Structure** | $W_{2ki}\left( t \right)=\alpha^{\left( 2 \right)}\left( b_{0ki}^{\left( 2 \right)}+b_{1ki}^{\left( 2 \right)}s_{ki} \right)+\alpha^{\left( 3 \right)}\left( b_{1k}^{\left( 3 \right)}treat_{ki} \right)$ |
| **3** | **Longitudinal Sub-Model** | $Y_{kij}=\beta_{10}+\beta_{11}t_{kij}+\beta_{12}treat_{ki}$ $+\beta_{13}\exp\left( -3*t_{kij} \right)$ $+b_{0ki}^{\left( 2 \right)}+b_{1ki}^{\left( 2 \right)}t_{kij}$ $+b_{0k}^{\left( 3 \right)}+b_{1k}^{(3)}treat_{ki}+\varepsilon_{kij}$ |
|  | **Time-to-event Sub-Model** | $\lambda_{ki}\left( t \right)= \lambda_{0}\left( t \right)\exp(\beta_{21}treat_{ki}+W_{2ki}\left( t \right))$ |
|  | **Association Structure** | $W_{2ki}\left( t \right)=\alpha^{\left( 2 \right)}\left( b_{0ki}^{\left( 2 \right)}+b_{1ki}^{\left( 2 \right)}s_{ki} \right)+\alpha^{\left( 3 \right)}\left( b_{0k}^{\left( 3 \right)}+b_{1k}^{\left( 3 \right)}treat_{ki} \right)$ |
| **4** | **Longitudinal Sub-Model** | $Y_{kij}=\beta_{10}+\beta_{11}t_{kij}+\beta_{12}treat_{ki}$ $+\beta_{13}{study}_{ki}+\beta_{14}treat_{ki}*{study}_{ki}$ $+\beta_{15}\exp\left( -3*t_{kij} \right)$ $+b_{0ki}^{(2)}+b_{1ki}^{(2)}t_{kij}+\varepsilon_{kij}$ |
|  | **Time-to-event Sub-Model** | $\lambda_{ki}\left( t \right)= \lambda_{0k}\left( t \right)\exp(\beta_{21}treat_{ki}+W_{2ki}\left( t \right))$ |
|  | **Association Structure** | $W_{2ki}\left( t \right)=\alpha^{\left( 2 \right)}(b_{0ki}^{\left( 2 \right)}+b_{1ki}^{\left( 2 \right)}s_{ki})$ |
| **5** | **Longitudinal Sub-Model** | $Y_{kij}=\beta_{10}+\beta_{11}t_{kij}+\beta_{12}treat_{ki}$ $+\beta_{13}{study}_{ki}+\beta_{14}\exp\left( -3*t_{kij} \right)$ $+b_{0ki}^{\left( 2 \right)}+b_{1ki}^{\left( 2 \right)}time_{kij}$ $+b_{1k}^{(3)}treat_{ki}+\varepsilon_{kij}$ |
|  | **Time-to-event Sub-Model** | $\lambda_{ki}\left( t \right)= \lambda_{0k}\left( t \right)\exp(\beta_{21}treat_{ki}+W_{2ki}\left( t \right))$ |
|  | **Association Structure** | $W_{2ki}\left( t \right)=\alpha^{\left( 2 \right)}\left( b_{0ki}^{\left( 2 \right)}+b_{1ki}^{\left( 2 \right)}s_{ki} \right)+\alpha^{\left( 3 \right)}\left( b_{1k}^{\left( 3 \right)}treat_{ki} \right)$ |

Supplemental Table S4: Specification of one stage model groups examined in the real data analysis of the INDANA dataset

# Graphical representations of the results of the INDANA analyses

## SBP and time to death
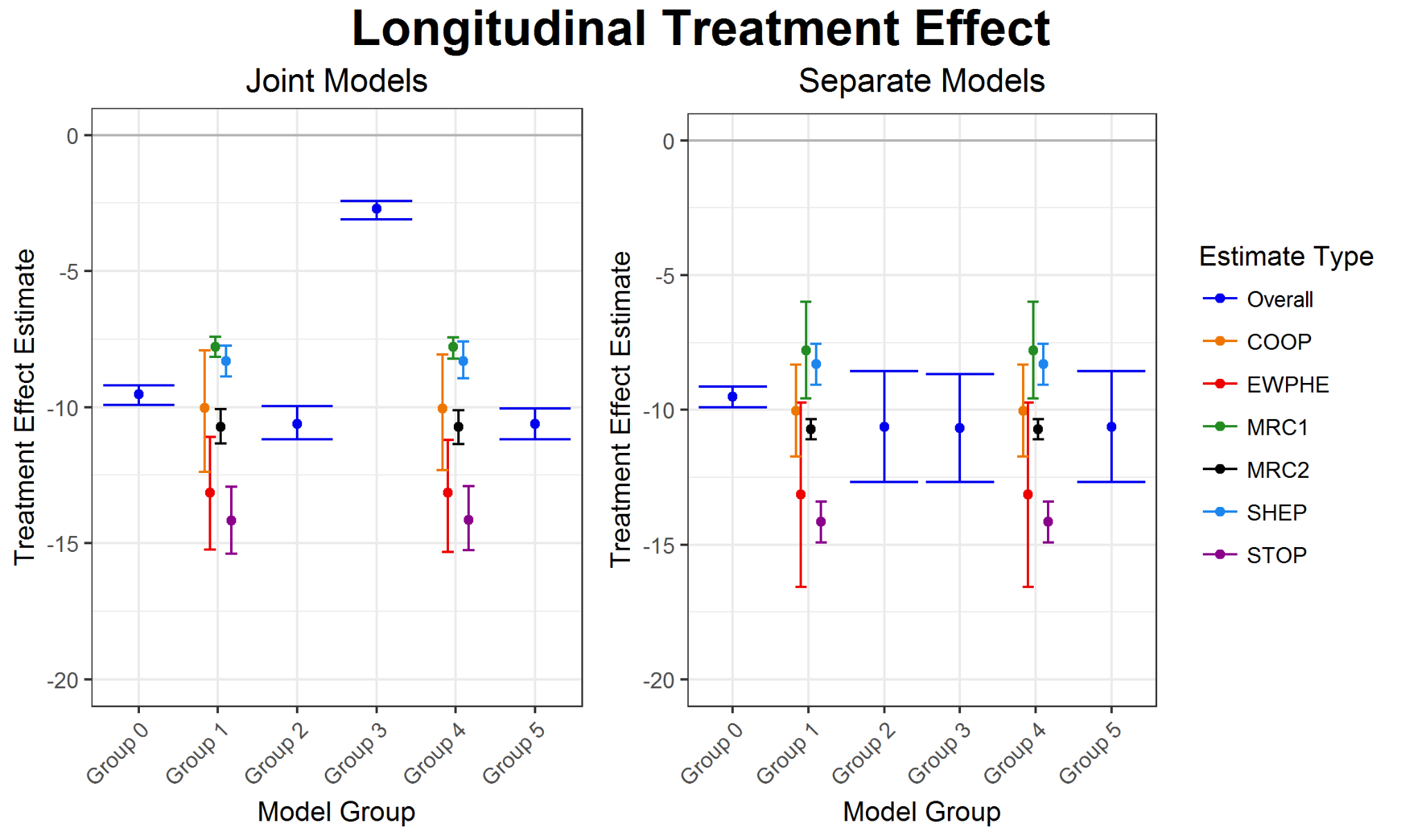


Supplemental Figure S4: Graphical representation of estimates of longitudinal treatment effect shown in Table 2 for one stage analysis of SBP and time to death


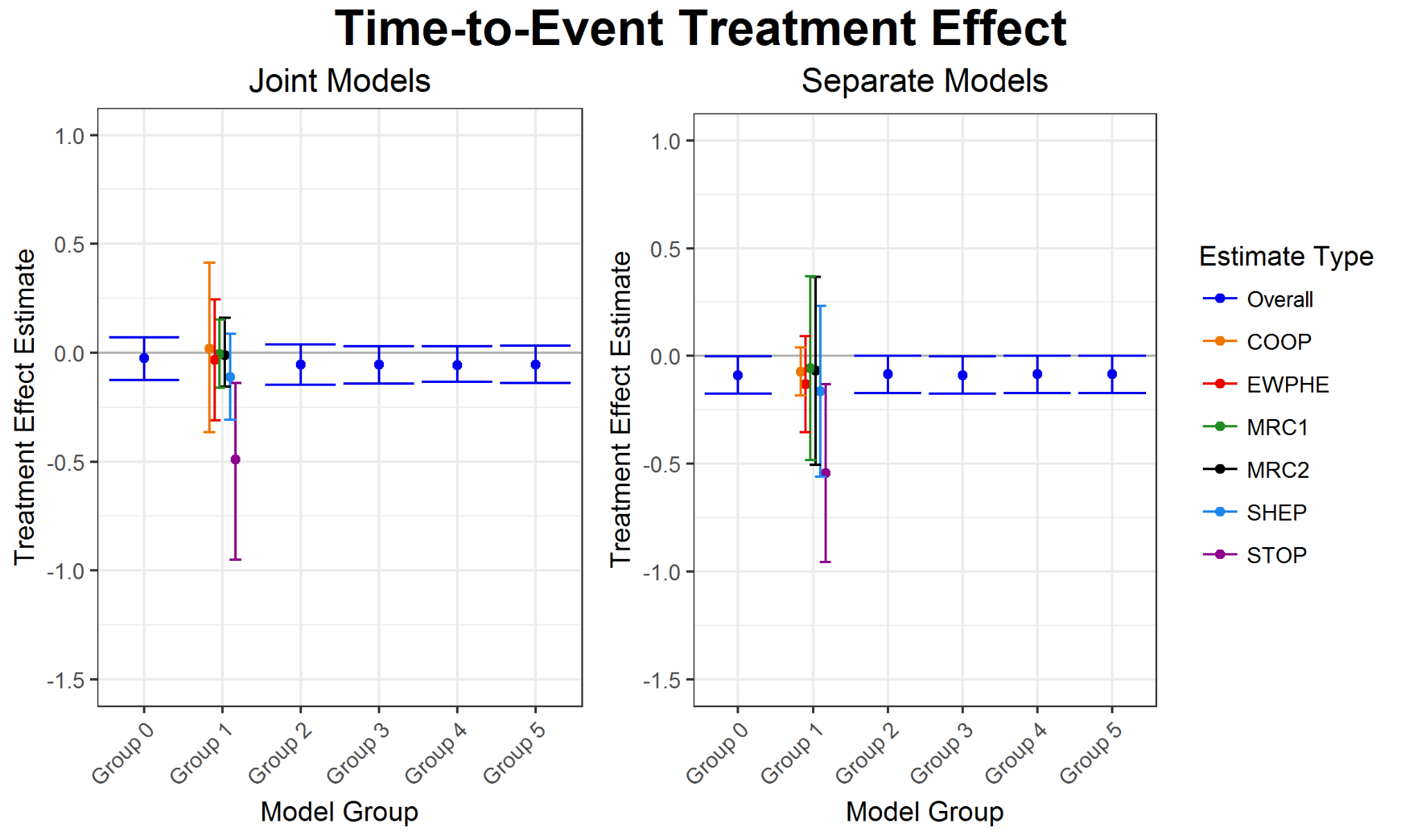


Supplemental Figure S5: Graphical representation of estimates of time-to-event treatment effect shown in Table 2 for one stage analysis of SBP and time to death


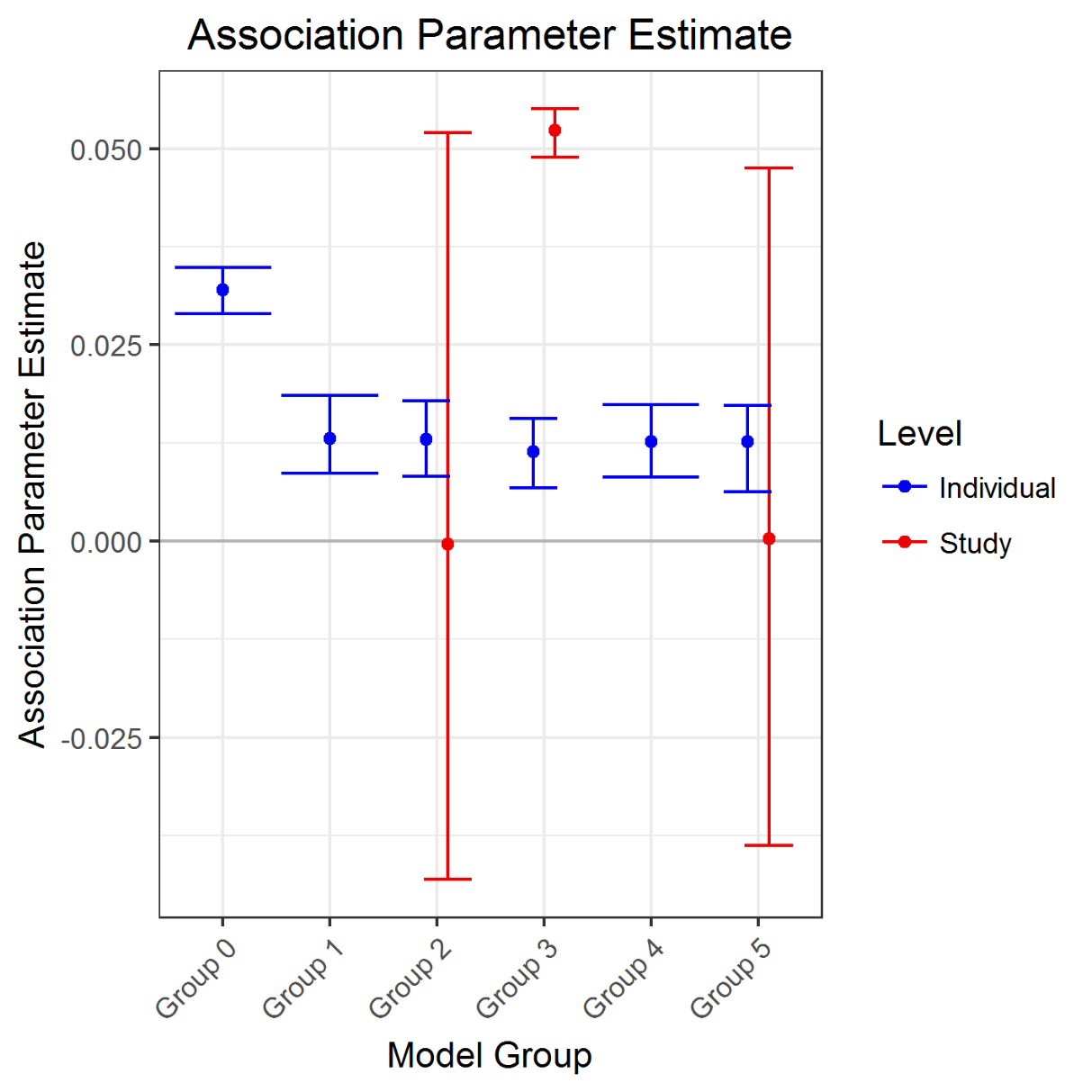


Supplemental Figure S6: Graphical representation of estimates of association parameters shown in Table 2 for one stage analysis of SBP and time to death

## SBP and time to MI


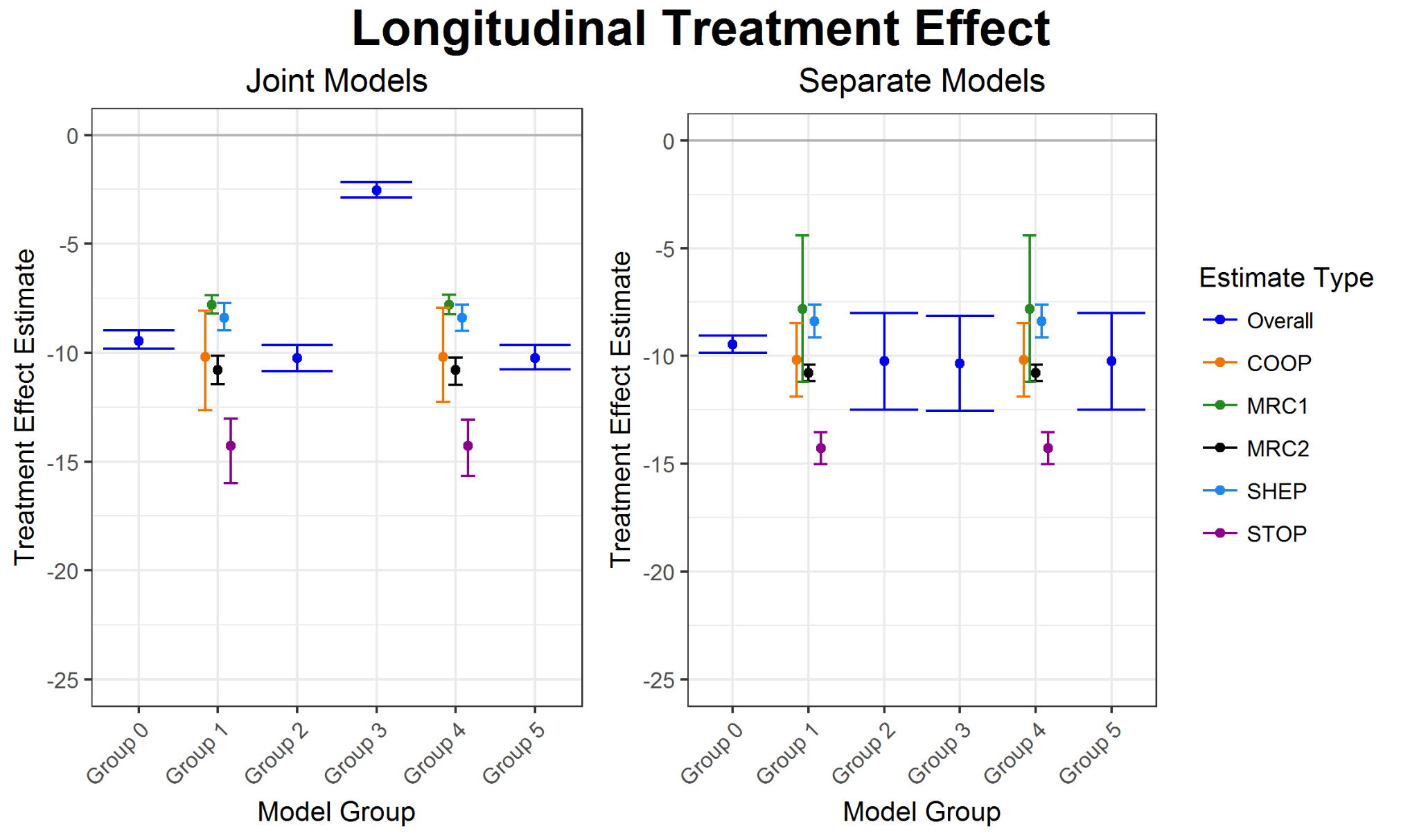


Supplemental Figure S7: Graphical representation of estimates of longitudinal treatment effect shown in Table 3 for one stage analysis of SBP and time to MI


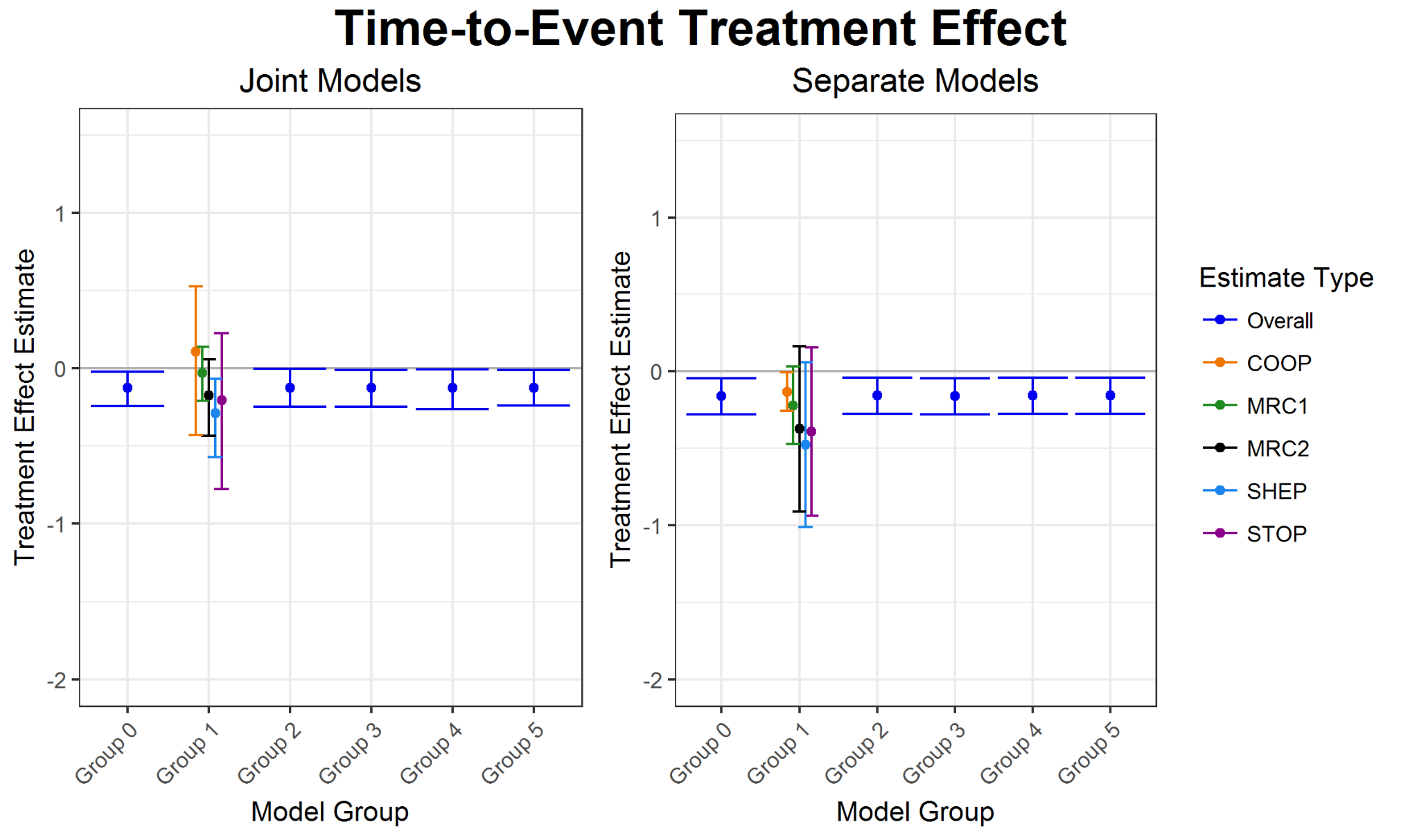


Supplemental Figure S8: Graphical representation of estimates of time-to-event treatment effect shown in Table 3 for one stage analysis of SBP and time to MI


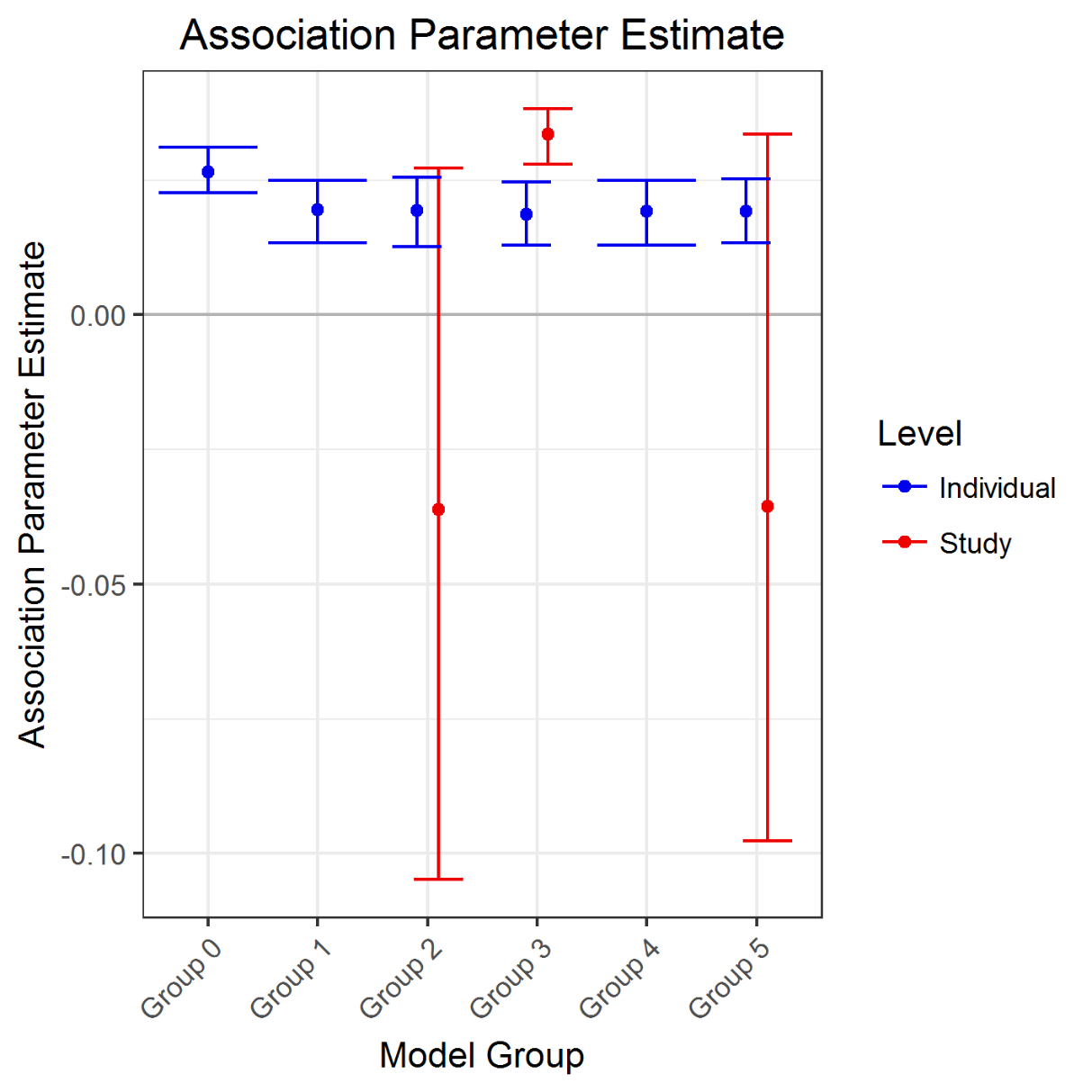


Supplemental Figure S9: Graphical representation of estimates of association parameters shown in Table 3 for one stage analysis of SBP and time to MI

## SBP and time to stroke
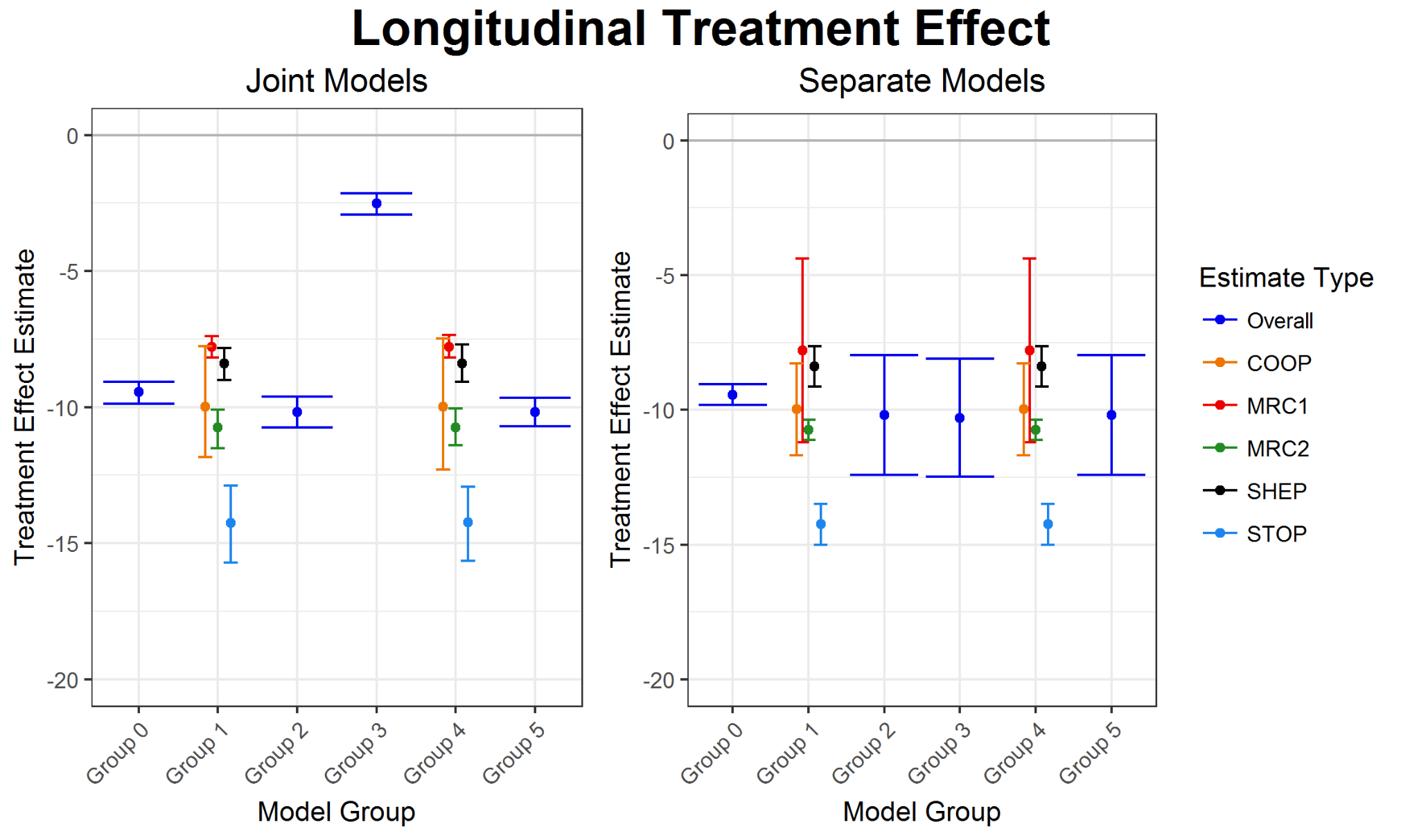


Supplemental Figure S10: Graphical representation of estimates of longitudinal treatment effect shown in Table 4 for one stage analysis of SBP and time to stroke


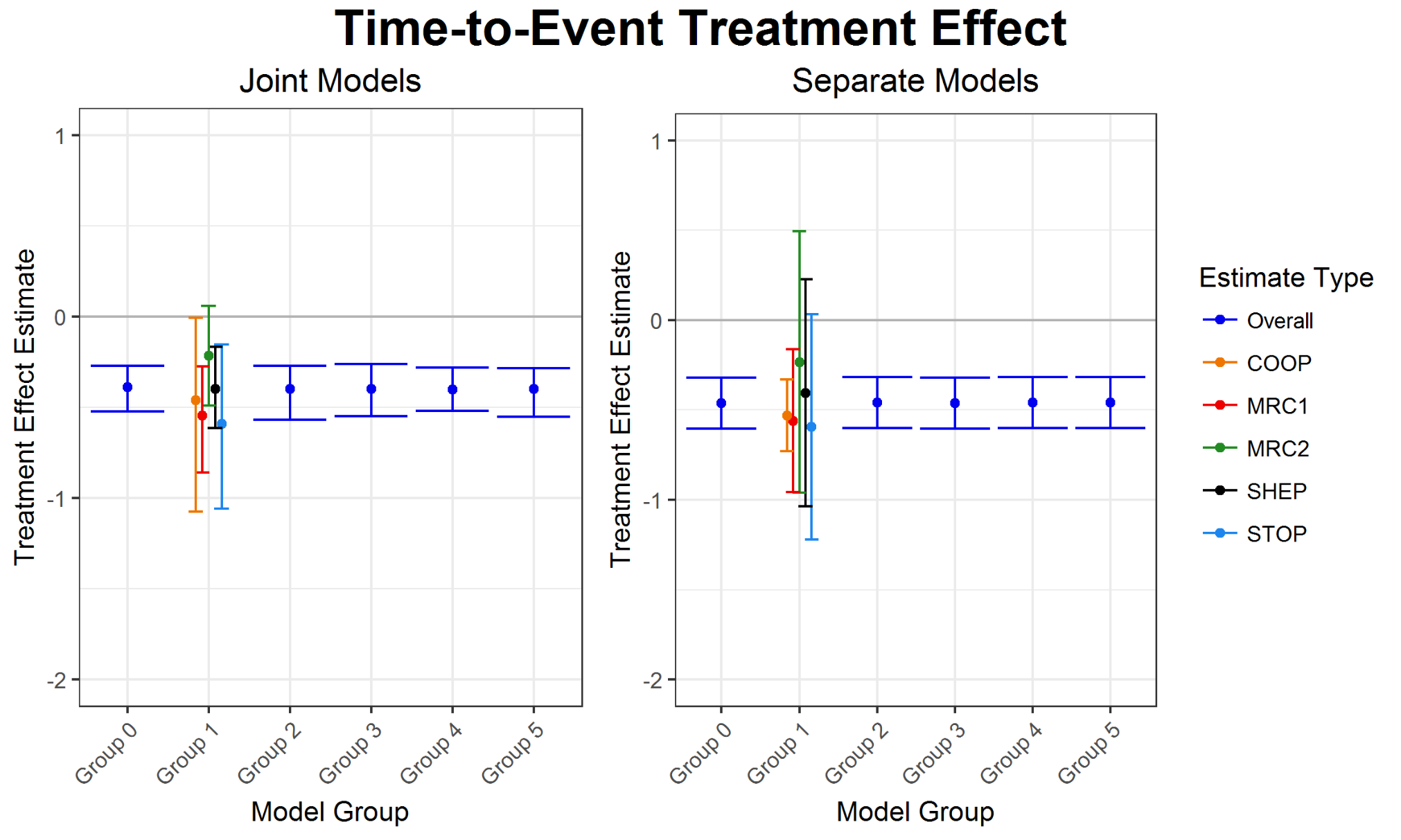


Supplemental Figure S11: Graphical representation of estimates of time-to-event treatment effect shown in Table 4 for one stage analysis of SBP and time to stroke


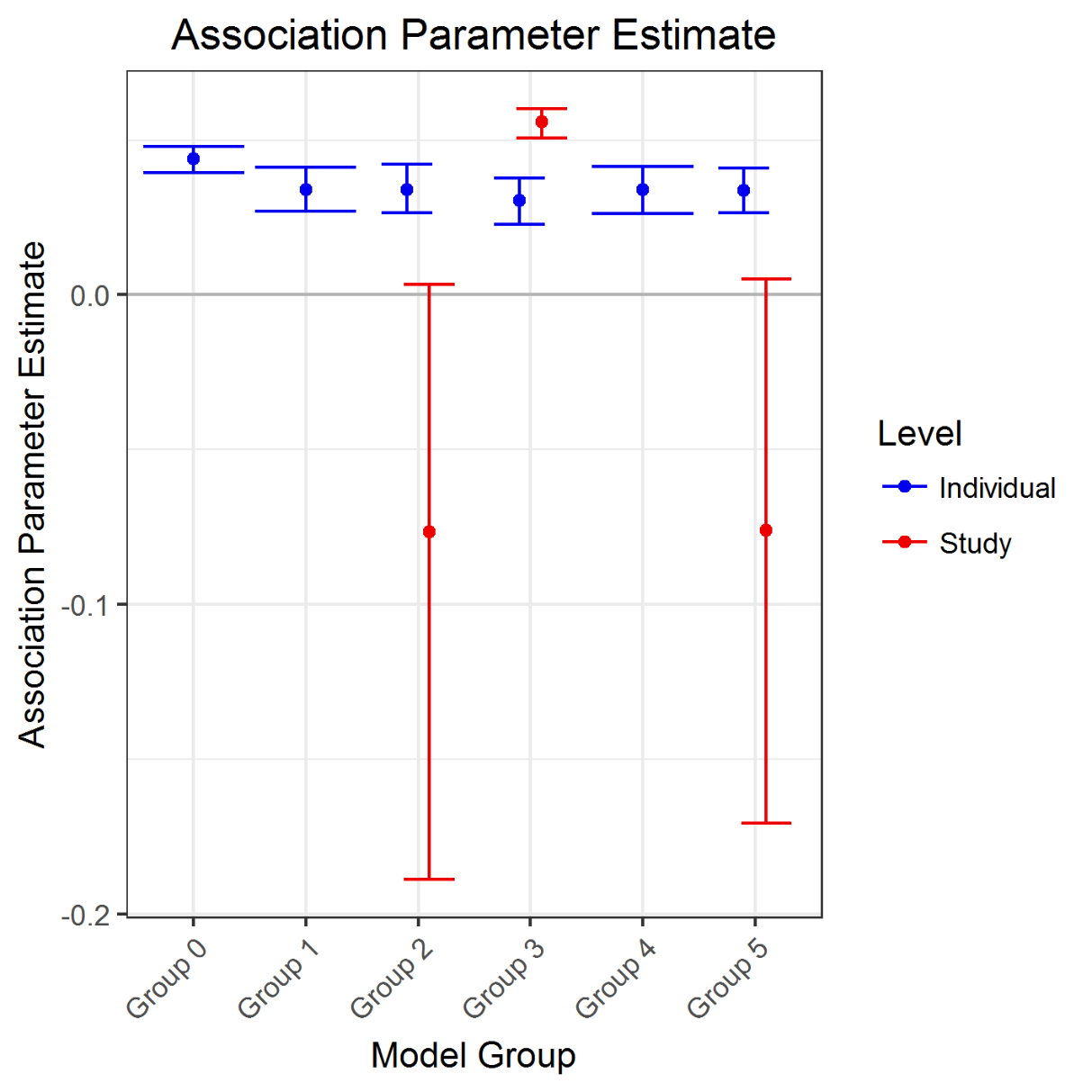


Supplemental Figure S12: Graphical representation of estimates of association parameters shown in Table 4 for one stage analysis of SBP and time to stroke

# Graphical representations of results from Simulation Set 1: Varying levels of Association

## Graphical representation of Tables 6-7 (mean estimates)


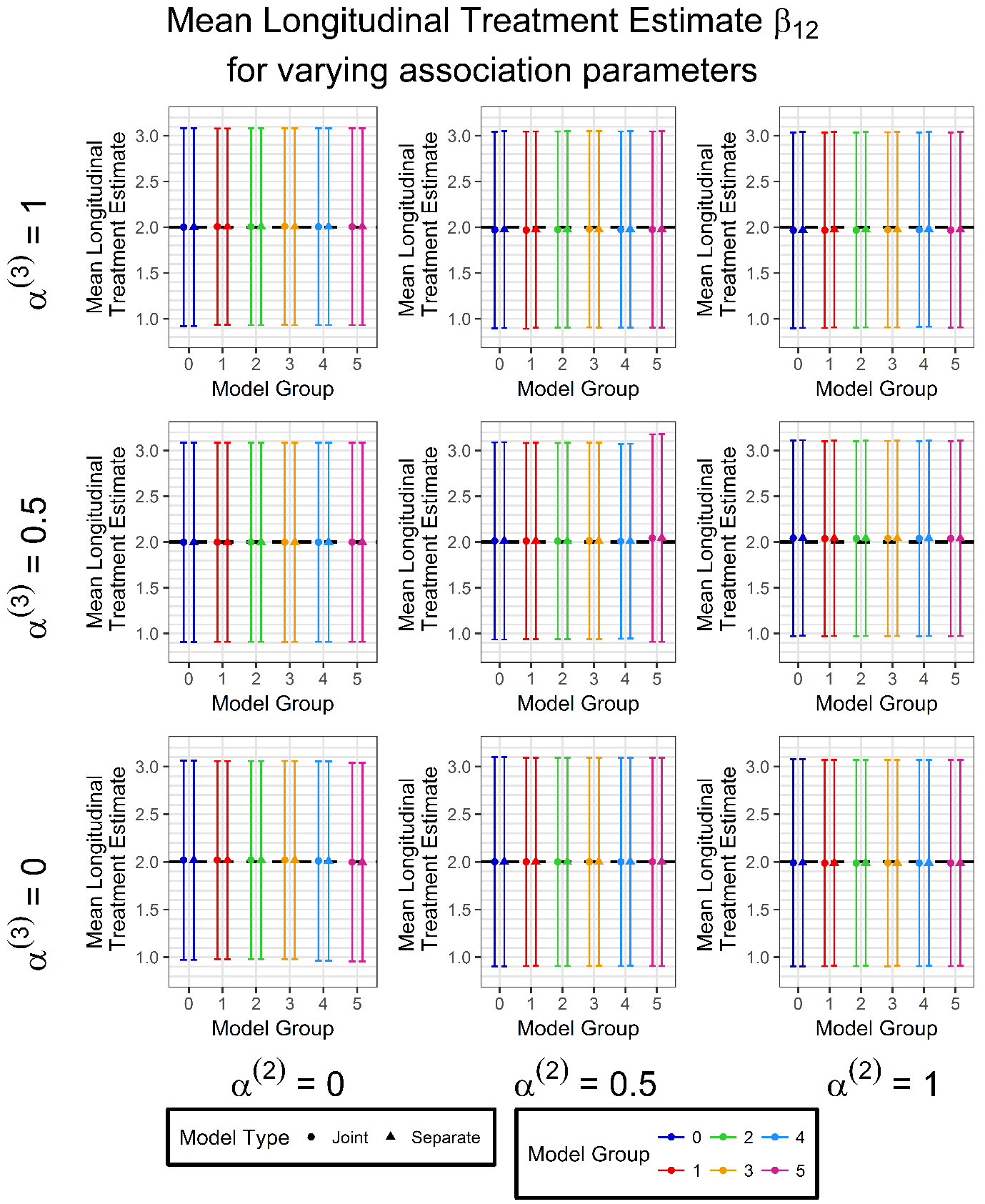


Supplemental Figure S13: Graphical representation of the mean longitudinal treatment effect ($\beta_{12}$) estimates from Table 6-7 for separate and joint models for simulation set 1, investigating varying association parameters. The dashed line identifies the “true” value of $\beta_{12}$ that the data was simulated under.


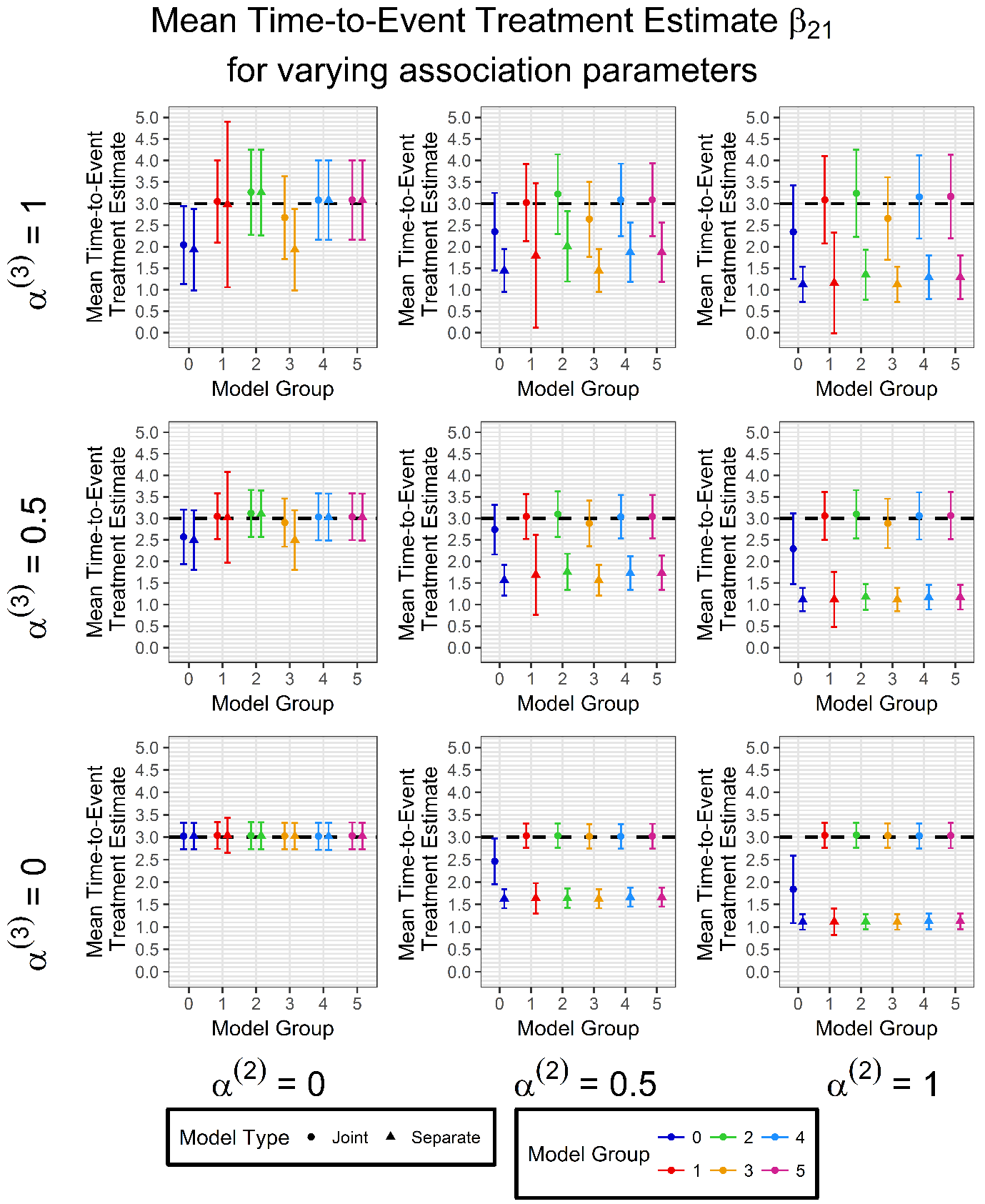


Supplemental Figure S14: Graphical representation of the mean time-to-event treatment effect ($\beta_{21}$) estimates from Tables 6-7 for separate and joint models for simulation set 1, investigating varying association parameters. The dashed line identifies the “true” value of $\beta_{21}$ that the data was simulated under.


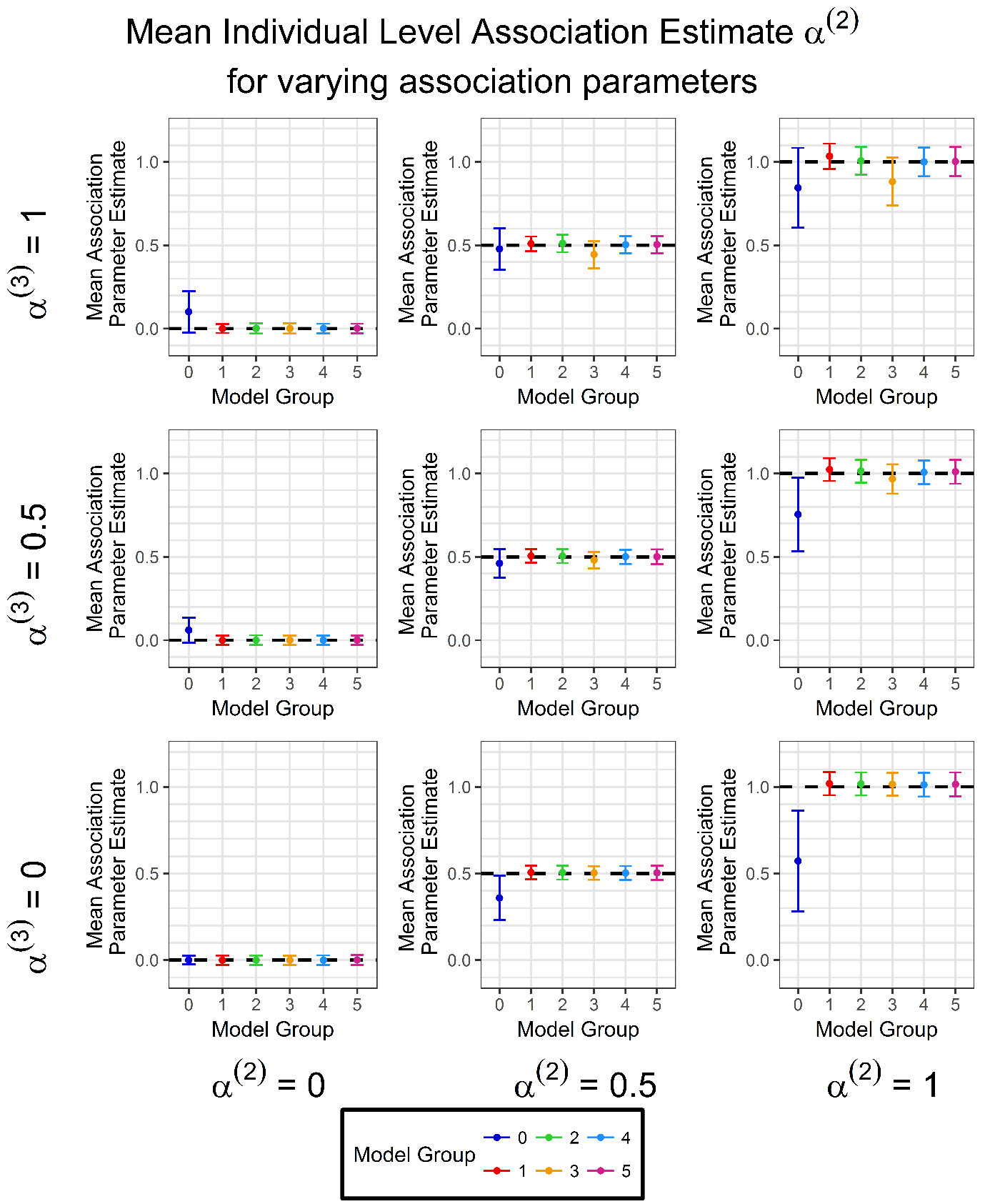


Supplemental Figure S15: Graphical representation of the mean individual level association parameter ($\alpha^{(2)}$) estimates from Table 6-7 for separate and joint models for simulation set 1, investigating varying association parameters. The dashed line identifies the “true” value of $\alpha^{\left( 2 \right)}$ that the data was simulated under.


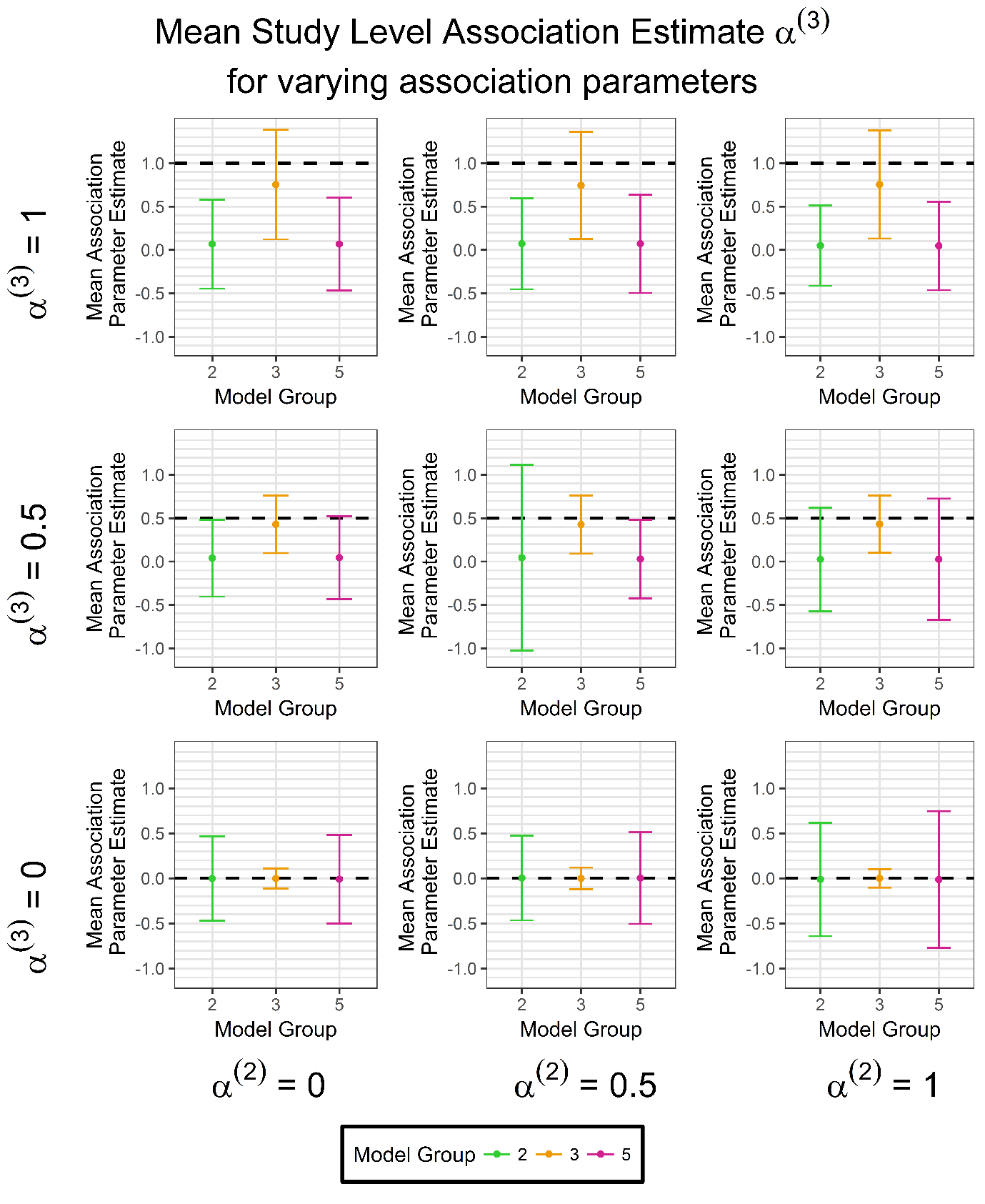


Supplemental Figure S16: Graphical representation of the mean study level association parameter ($\alpha^{(3)}$) estimates from Tables 6-7 for separate and joint models for simulation set 1, investigating varying association parameters. The dashed line identifies the “true” value of $\alpha^{\left( 3 \right)}$ that the data was simulated under.

## Graphical representation of Tables 6-7 (point estimates)


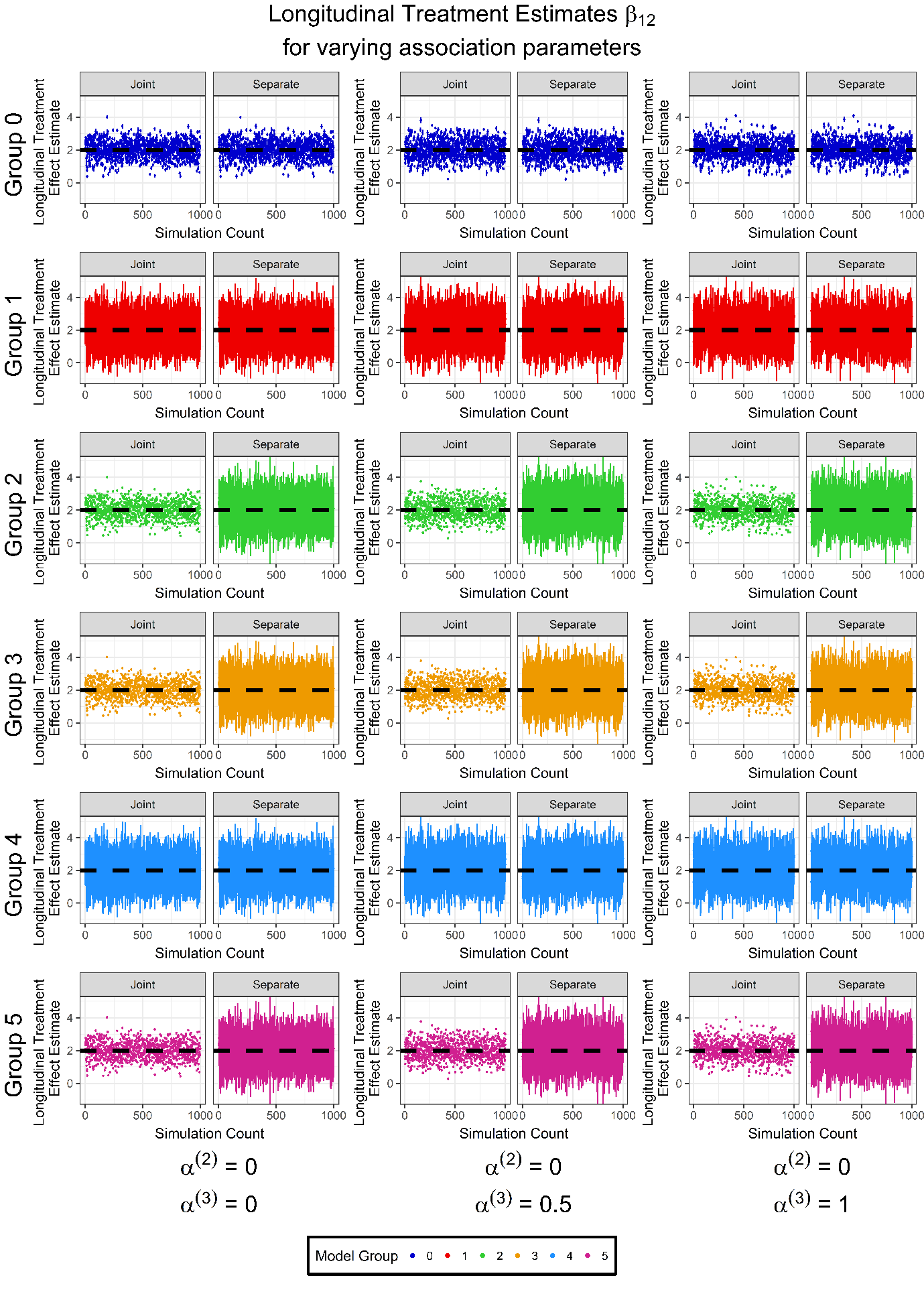


Supplemental Figure S17: Point estimates and confidence intervals for longitudinal treatment effect parameter ($\boldsymbol{\beta}_{\boldsymbol{12}}$) for Simulation Group 1 investigating varying association parameters (values of association parameters that the data was simulated under are stated under each column). The dashed line indicates the value of $\boldsymbol{\beta}_{\boldsymbol{12}}$ that the data was simulated under.


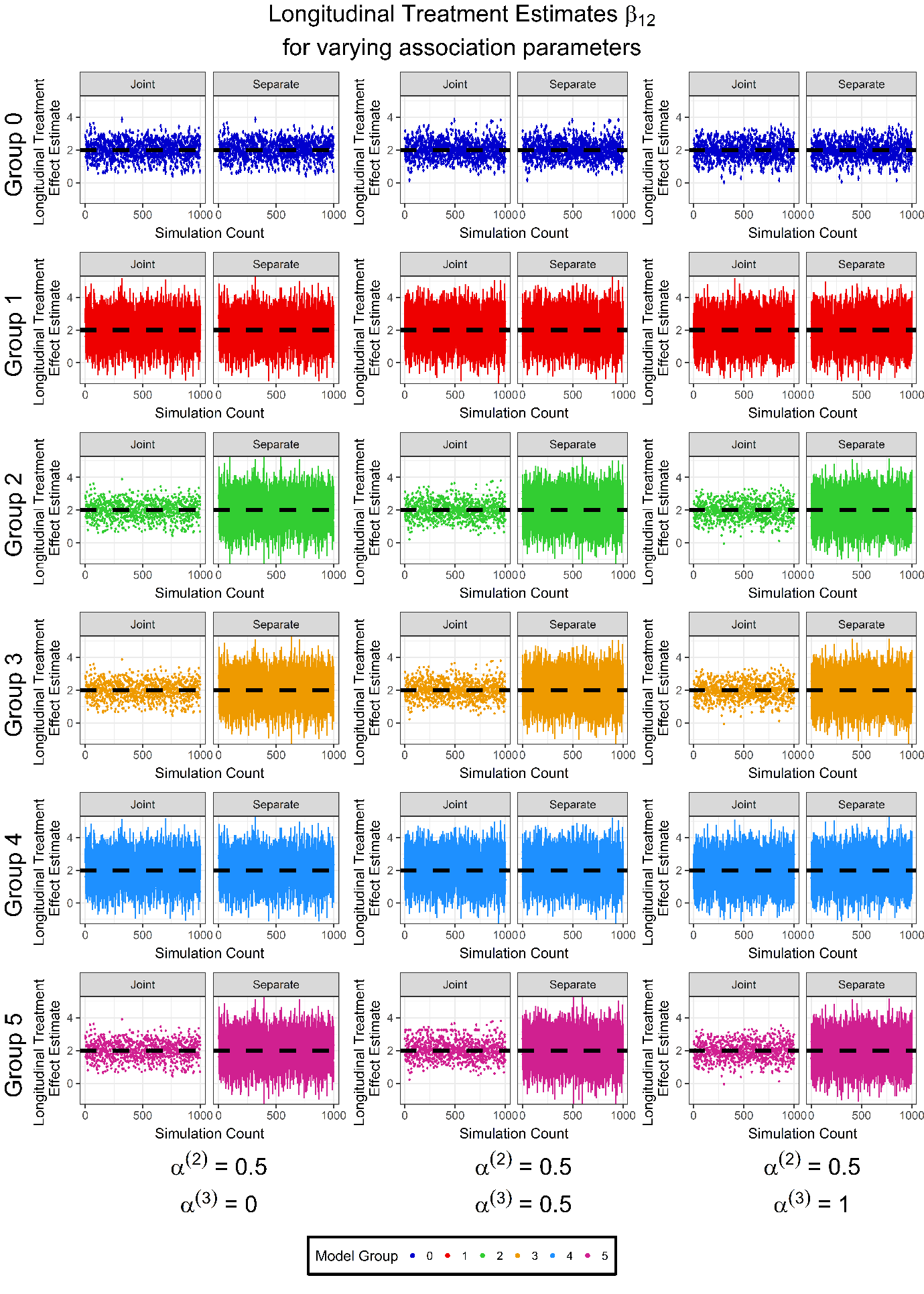


Supplemental Figure S18: Point estimates and confidence intervals for longitudinal treatment effect parameter ($\boldsymbol{\beta}_{\boldsymbol{12}}$) for Simulation Group 1 investigating varying association parameters (values of association parameters that the data was simulated under are stated under each column). The dashed line indicates the value of $\boldsymbol{\beta}_{\boldsymbol{12}}$ that the data was simulated under.


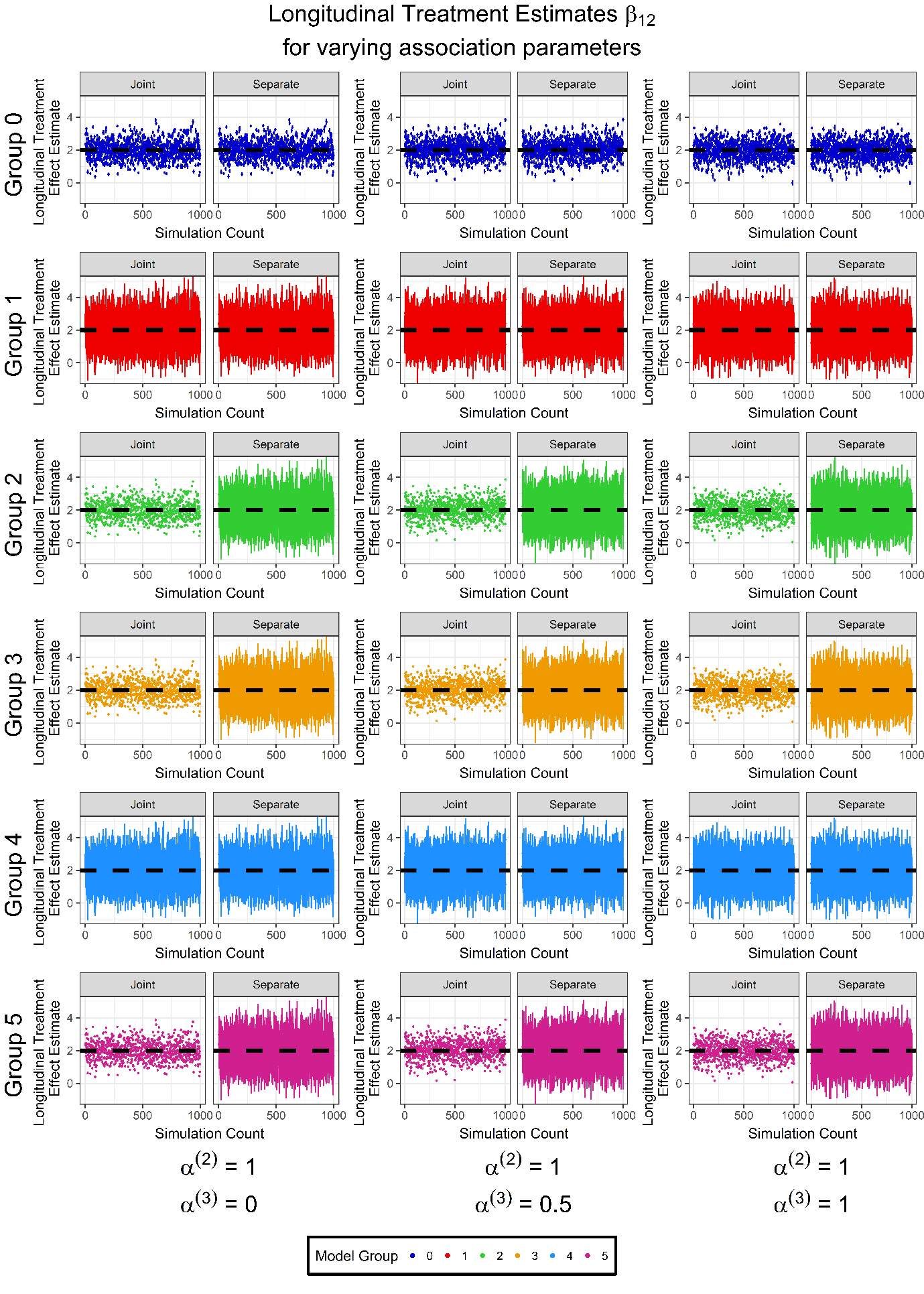


Supplemental Figure S19: Point estimates and confidence intervals for longitudinal treatment effect parameter ($\boldsymbol{\beta}_{\boldsymbol{12}}$) for Simulation Group 1 investigating varying association parameters (values of association parameters that the data was simulated under are stated under each column). The dashed line indicates the value of $\boldsymbol{\beta}_{\boldsymbol{12}}$ that the data was simulated under.


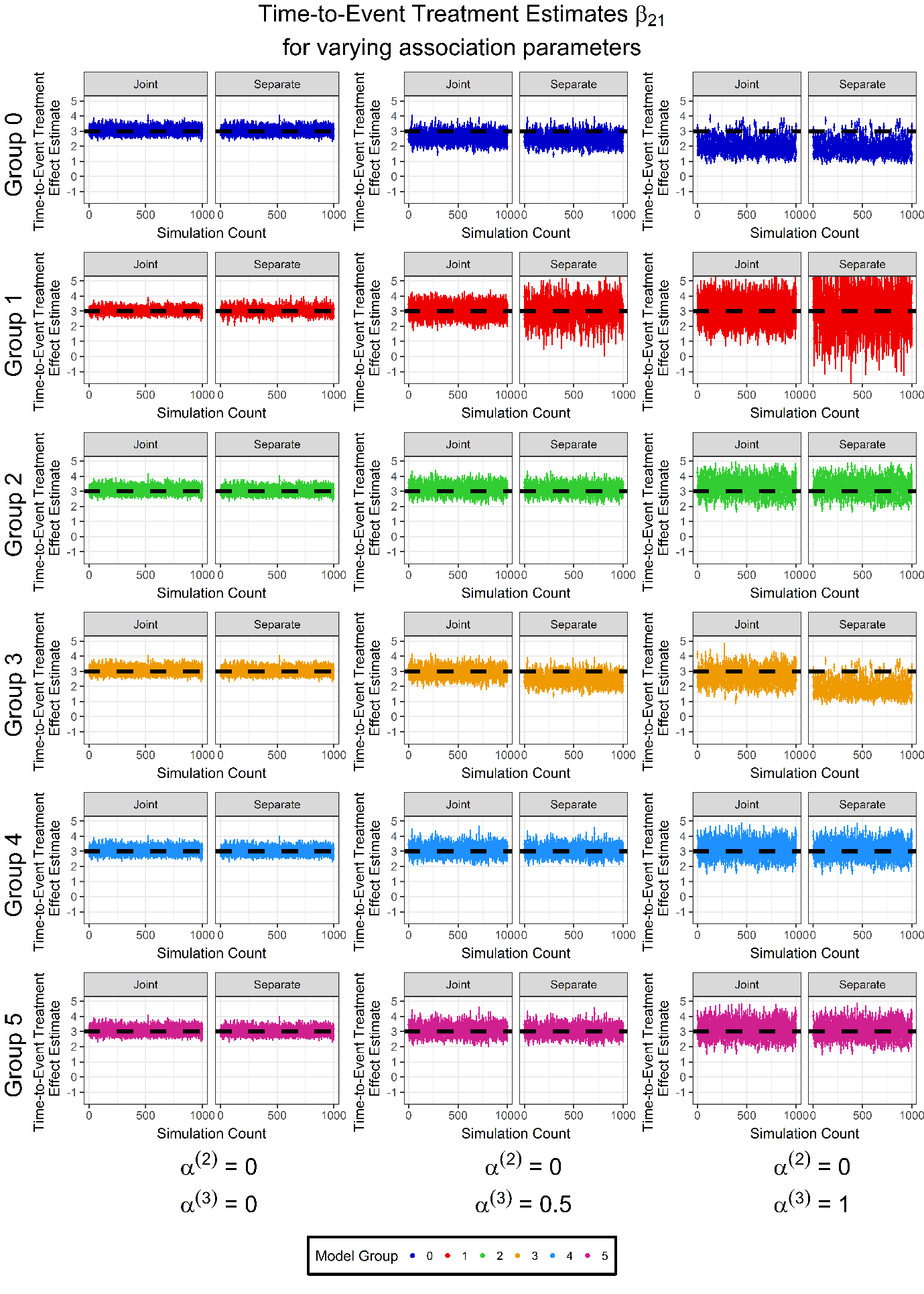


Supplemental Figure S20: Point estimates and confidence intervals for longitudinal treatment effect parameter ($\boldsymbol{\beta}_{\boldsymbol{21}}$) for Simulation Group 1 investigating varying association parameters (values of association parameters that the data was simulated under are stated under each column). The dashed line indicates the value of $\boldsymbol{\beta}_{\boldsymbol{21}}$ that the data was simulated under.


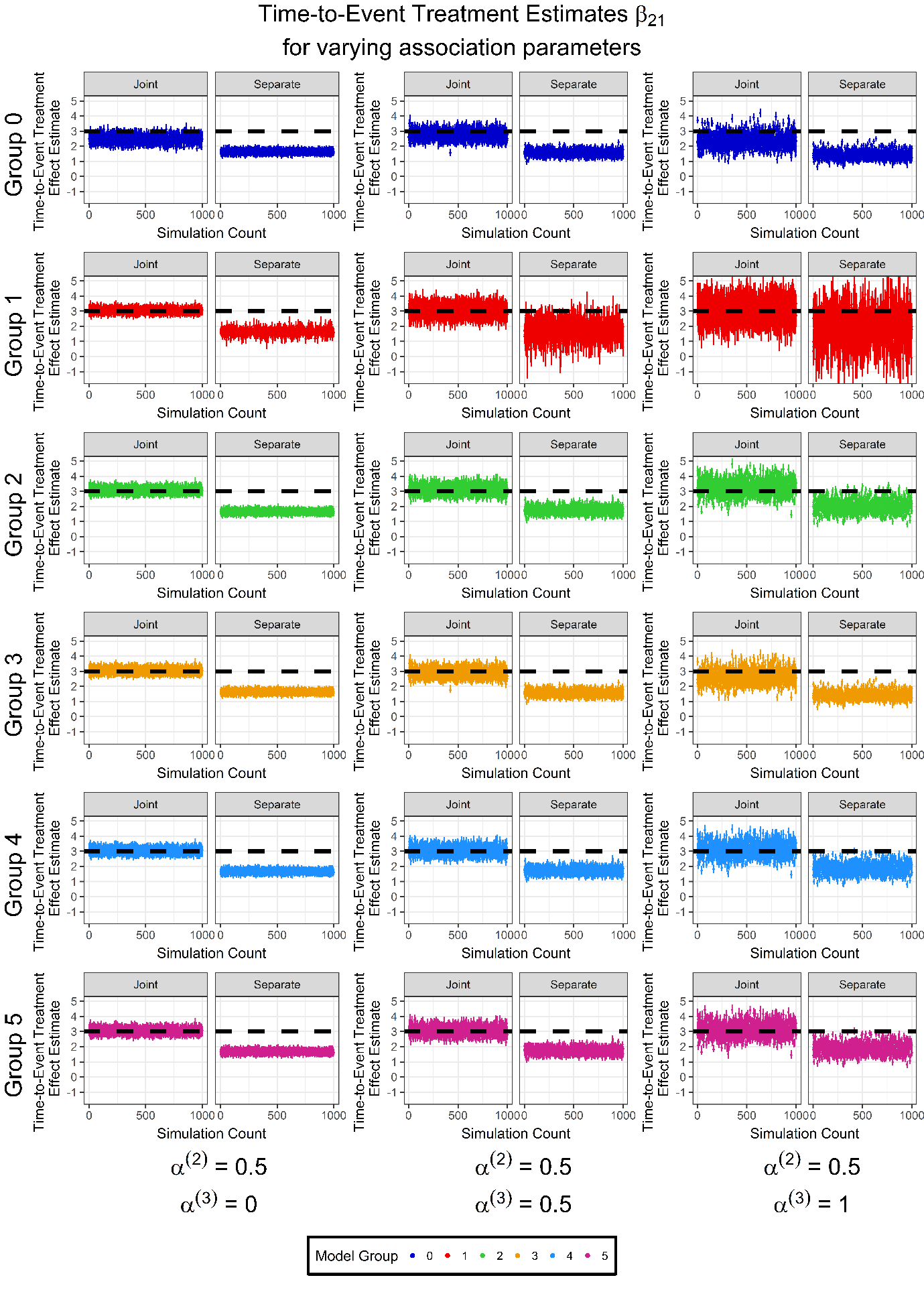


Supplemental Figure S21: Point estimates and confidence intervals for longitudinal treatment effect parameter ($\boldsymbol{\beta}_{\boldsymbol{21}}$) for Simulation Group 1 investigating varying association parameters (values of association parameters that the data was simulated under are stated under each column). The dashed line indicates the value of $\boldsymbol{\beta}_{\boldsymbol{21}}$ that the data was simulated under.


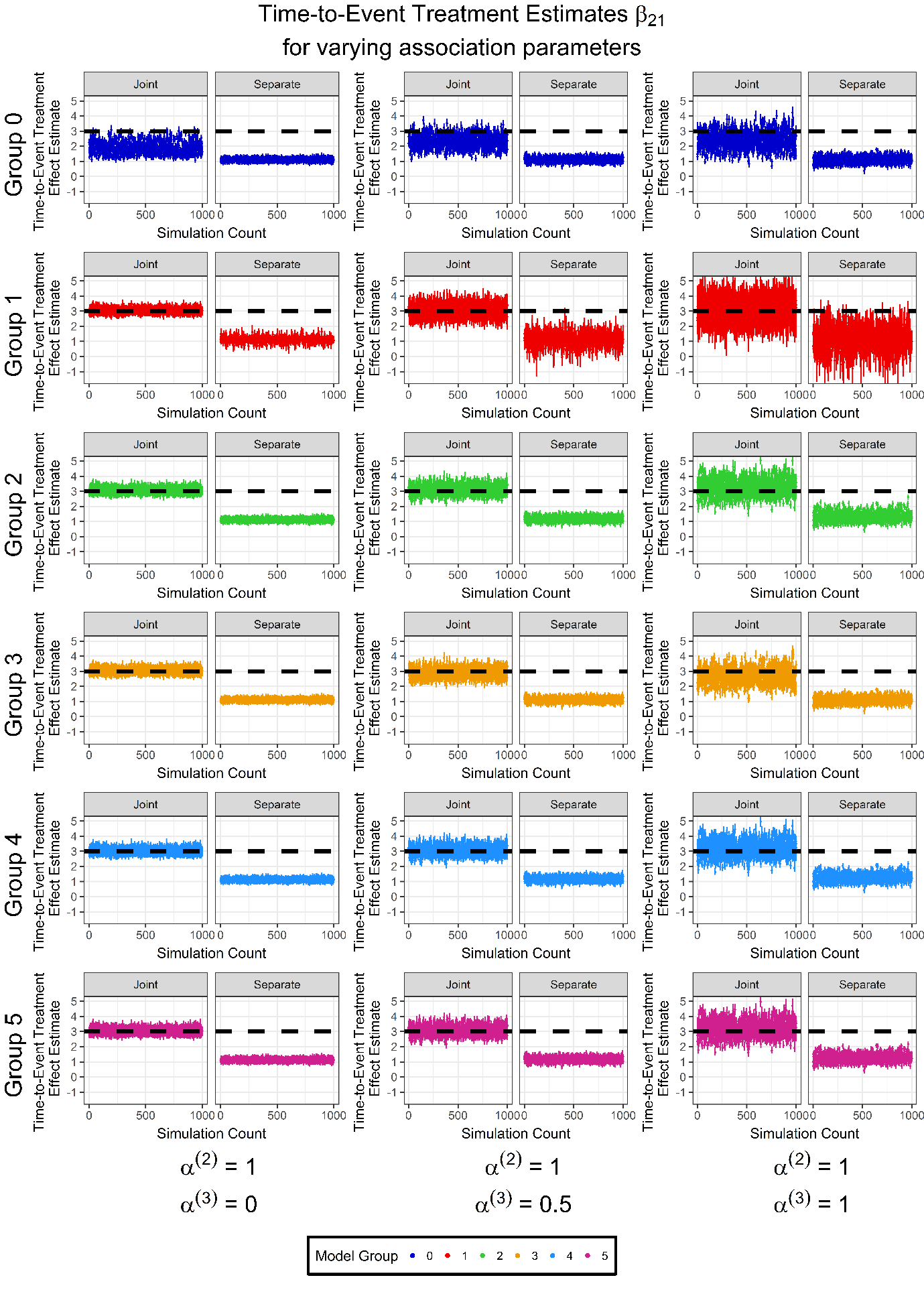


Supplemental Figure S22: Point estimates and confidence intervals for longitudinal treatment effect parameter ($\boldsymbol{\beta}_{\boldsymbol{21}}$) for Simulation Group 1 investigating varying association parameters (values of association parameters that the data was simulated under are stated under each column). The dashed line indicates the value of $\boldsymbol{\beta}_{\boldsymbol{21}}$ that the data was simulated under.


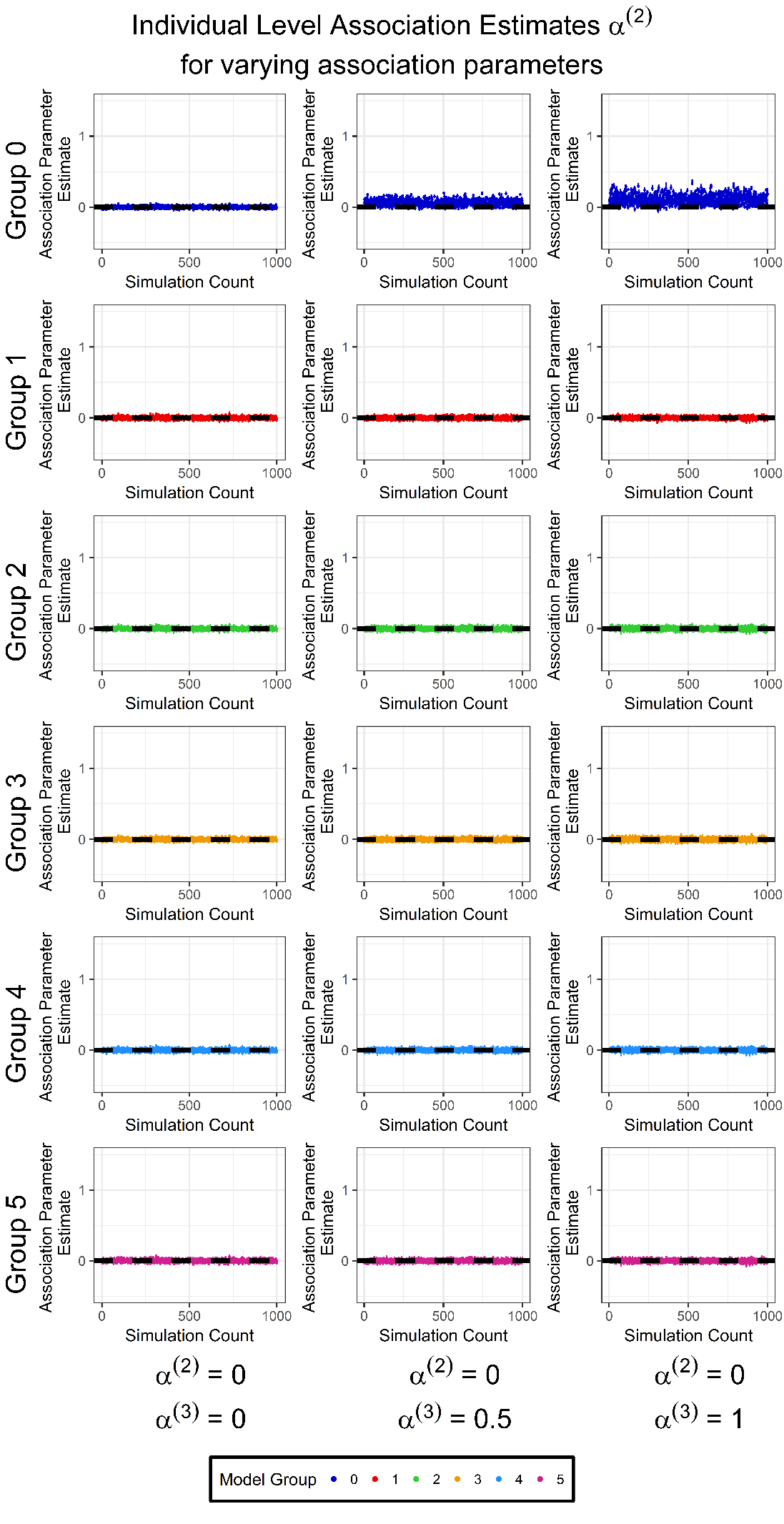


Supplemental Figure S23: Point estimates and confidence intervals for longitudinal treatment effect parameter ($\boldsymbol{\alpha}^{\left( \boldsymbol{2} \right)}$) for Simulation Group 1 investigating varying association parameters (values of association parameters that the data was simulated under are stated under each column). The dashed line indicates the value of $\boldsymbol{\alpha}^{\left( \boldsymbol{2} \right)}$ that the data was simulated under.


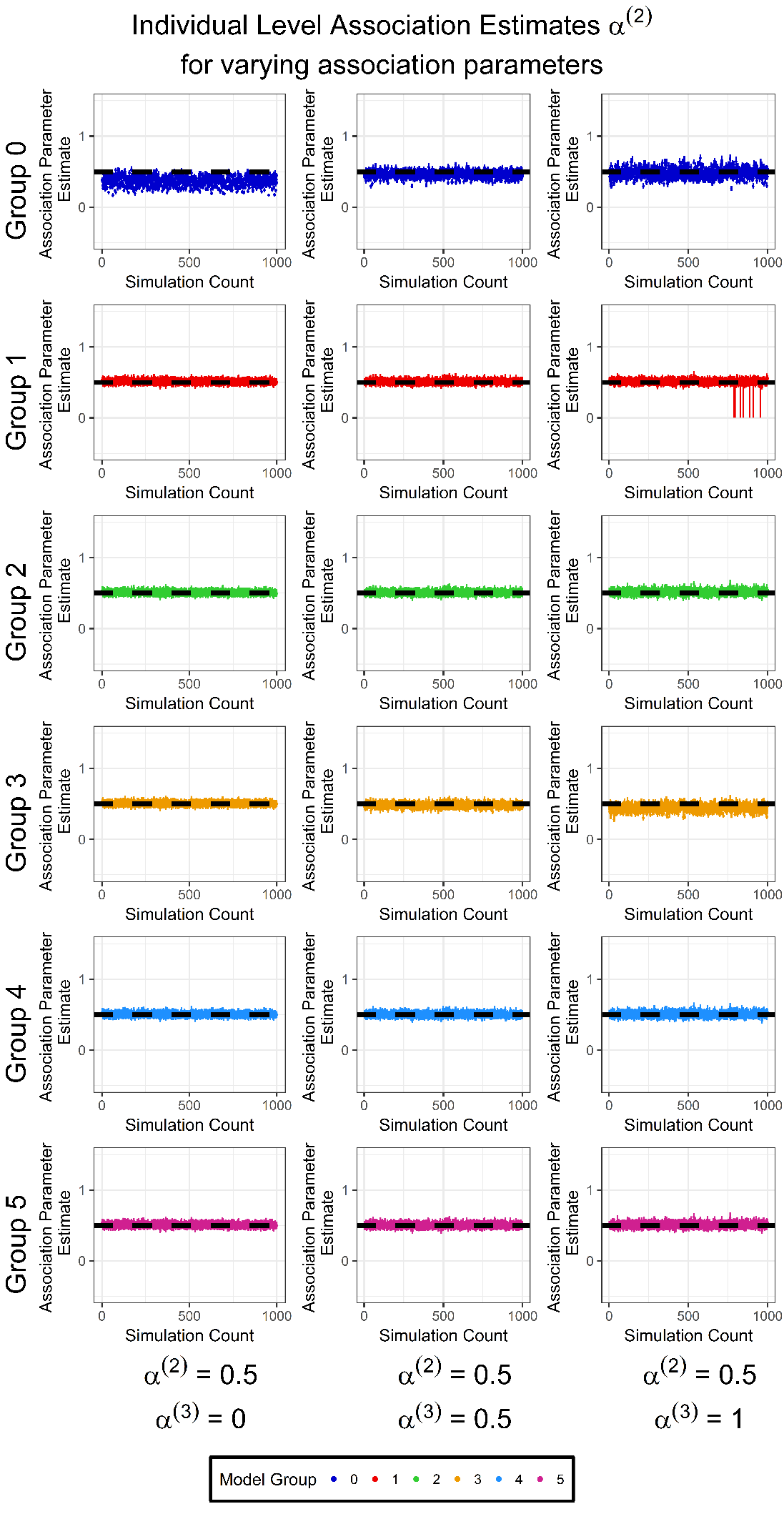


Supplemental Figure S24: Point estimates and confidence intervals for longitudinal treatment effect parameter ($\boldsymbol{\alpha}^{\left( \boldsymbol{2} \right)}$) for Simulation Group 1 investigating varying association parameters (values of association parameters that the data was simulated under are stated under each column). The dashed line indicates the value of $\boldsymbol{\alpha}^{\left( \boldsymbol{2} \right)}$ that the data was simulated under.


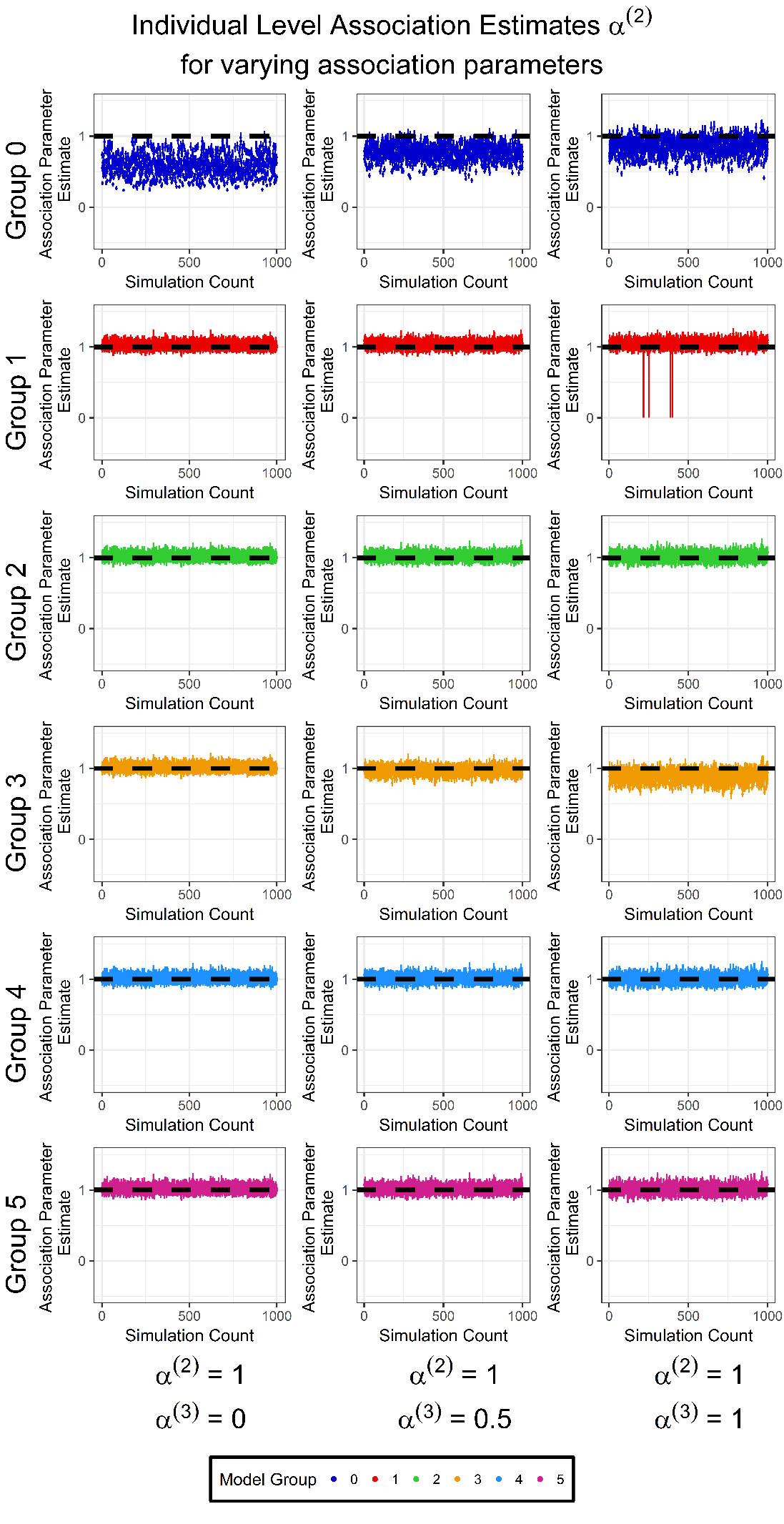


Supplemental Figure S25: Point estimates and confidence intervals for longitudinal treatment effect parameter ($\boldsymbol{\alpha}^{\left( \boldsymbol{2} \right)}$) for Simulation Group 1 investigating varying association parameters (values of association parameters that the data was simulated under are stated under each column). The dashed line indicates the value of $\boldsymbol{\alpha}^{\left( \boldsymbol{2} \right)}$ that the data was simulated under.


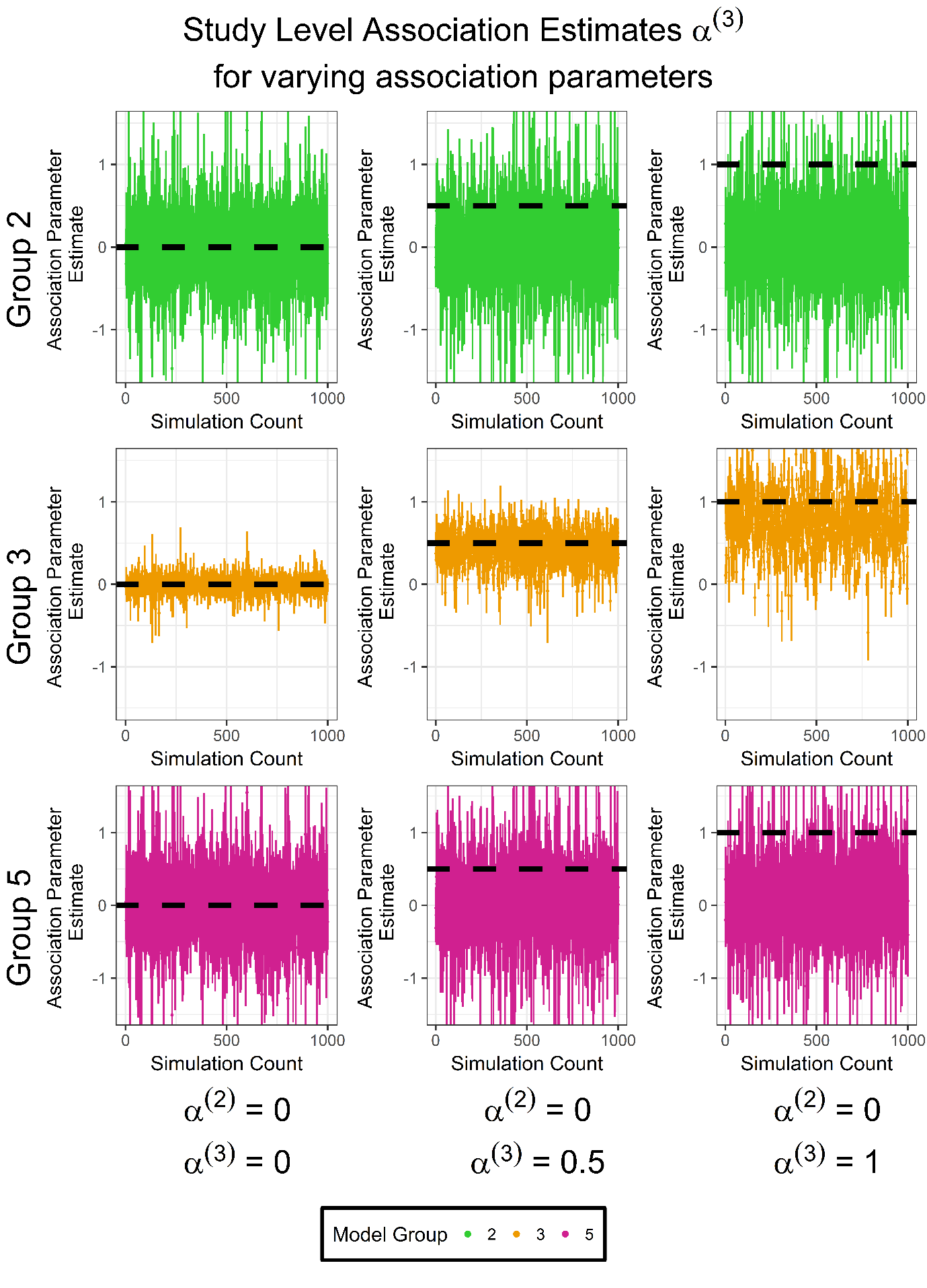


Supplemental Figure S26: Point estimates and confidence intervals for longitudinal treatment effect parameter ($\boldsymbol{\alpha}^{\left( \boldsymbol{3} \right)}$) for Simulation Group 1 investigating varying association parameters (values of association parameters that the data was simulated under are stated under each column). The dashed line indicates the value of $\boldsymbol{\alpha}^{\left( \boldsymbol{3} \right)}$ that the data was simulated under.


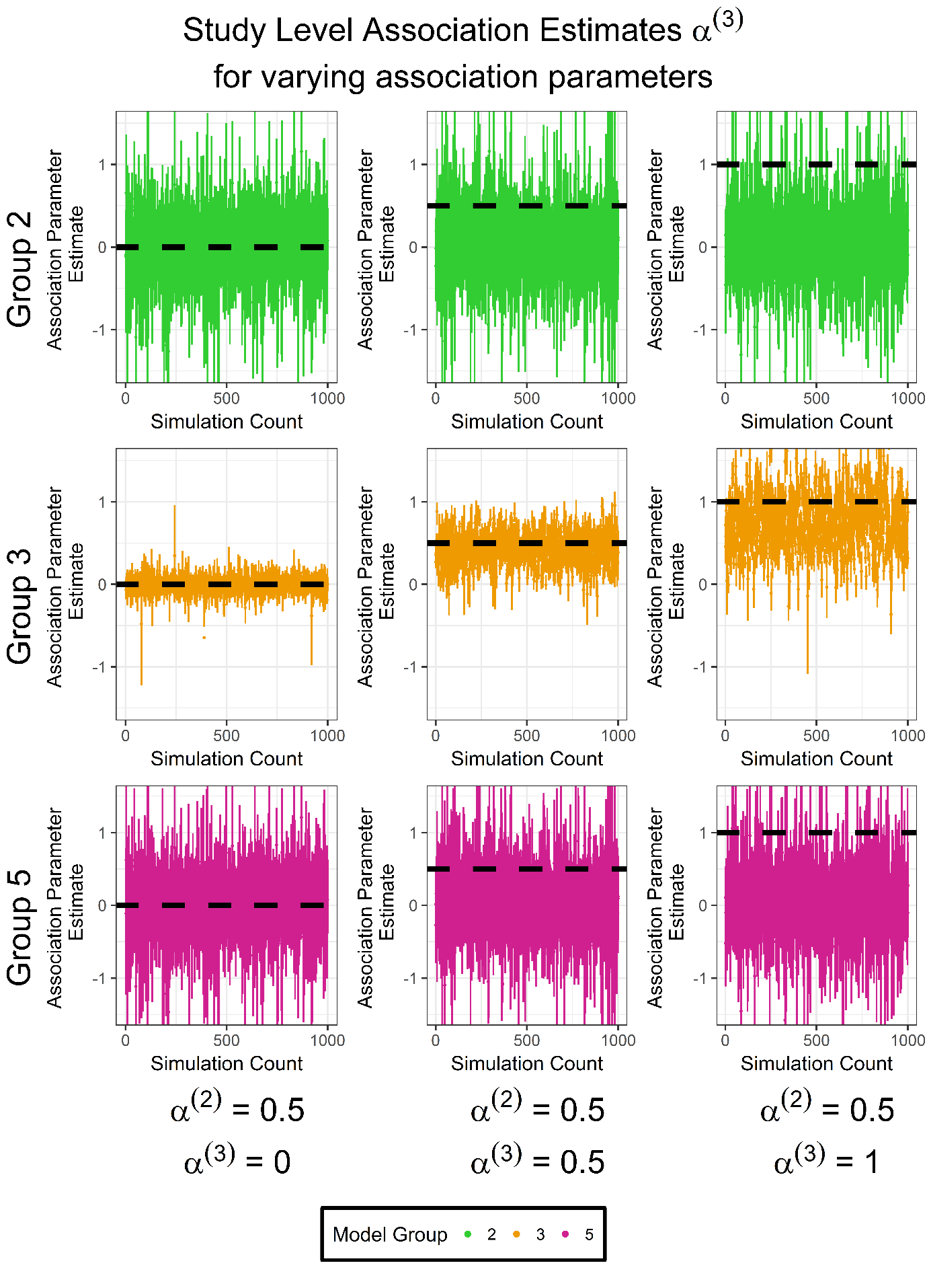


Supplemental Figure S27: Point estimates and confidence intervals for longitudinal treatment effect parameter ($\boldsymbol{\alpha}^{\left( \boldsymbol{3} \right)}$) for Simulation Group 1 investigating varying association parameters (values of association parameters that the data was simulated under are stated under each column). The dashed line indicates the value of $\boldsymbol{\alpha}^{\left( \boldsymbol{3} \right)}$ that the data was simulated under.


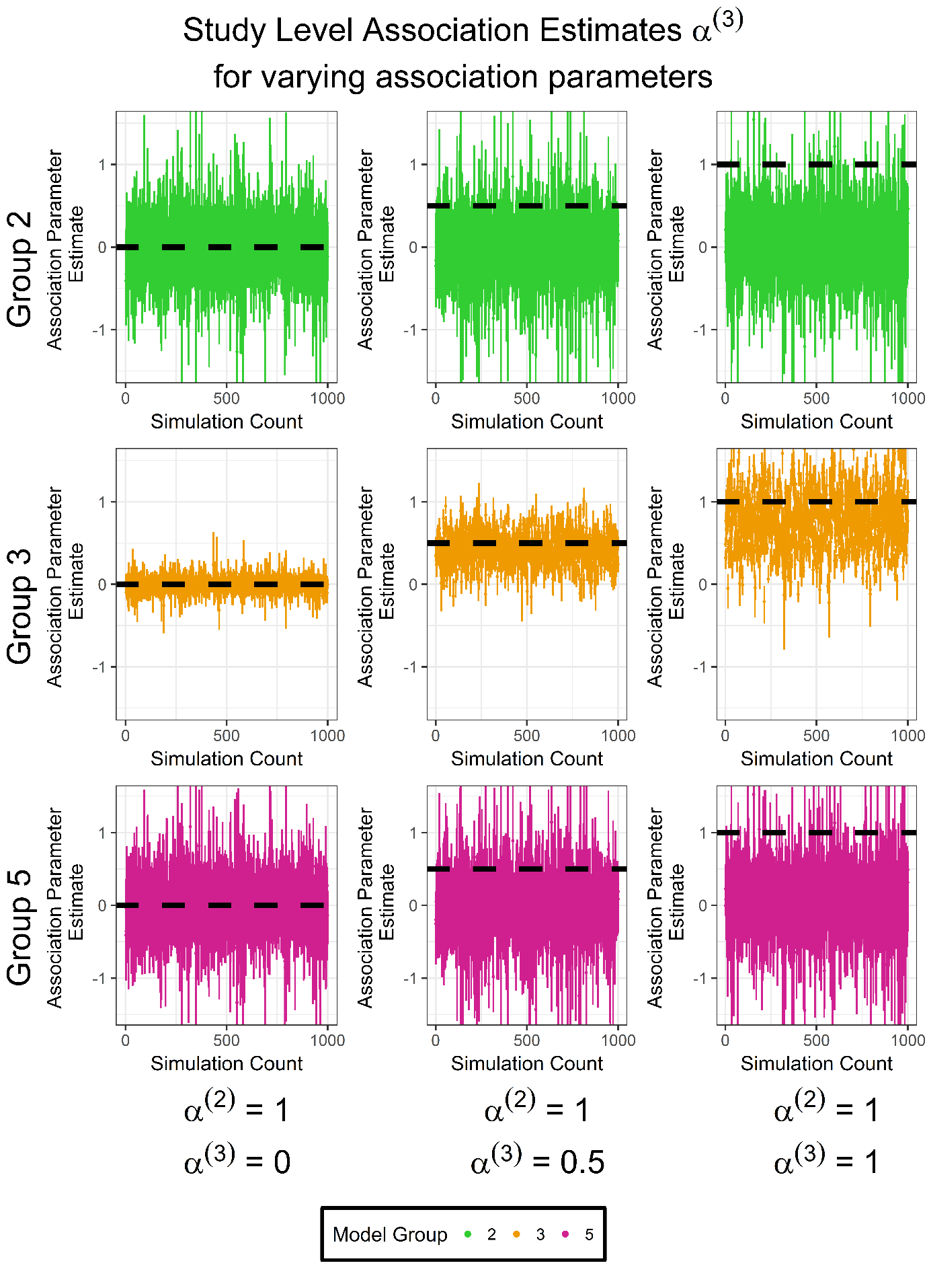


Supplemental Figure S28: Point estimates and confidence intervals for longitudinal treatment effect parameter ($\boldsymbol{\alpha}^{\left( \boldsymbol{3} \right)}$) for Simulation Group 1 investigating varying association parameters (values of association parameters that the data was simulated under are stated under each column). The dashed line indicates the value of $\boldsymbol{\alpha}^{\left( \boldsymbol{3} \right)}$ that the data was simulated under.

# Graphical representations of results from Simulation Set 2: Varying numbers of included studies

## Graphical representation of Table 8 (mean estimates)


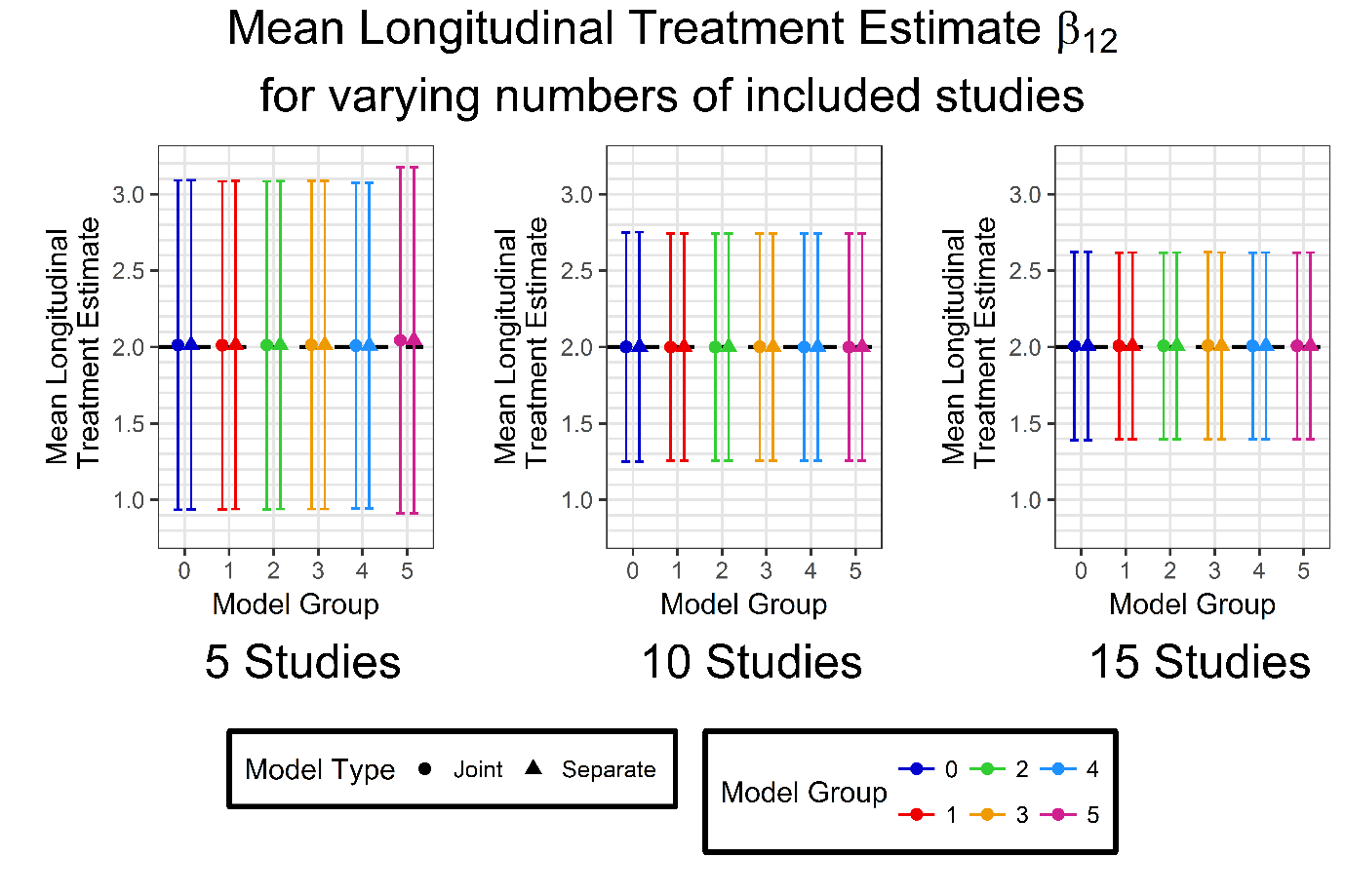


Supplemental Figure S29: Graphical representation of longitudinal treatment effect ($\beta_{12}$) estimates shown in Table 8 for simulation set 2: investigation of varying numbers of included studies. The dashed line indicates the “true” value of $\beta_{12}$ that the data was simulated under


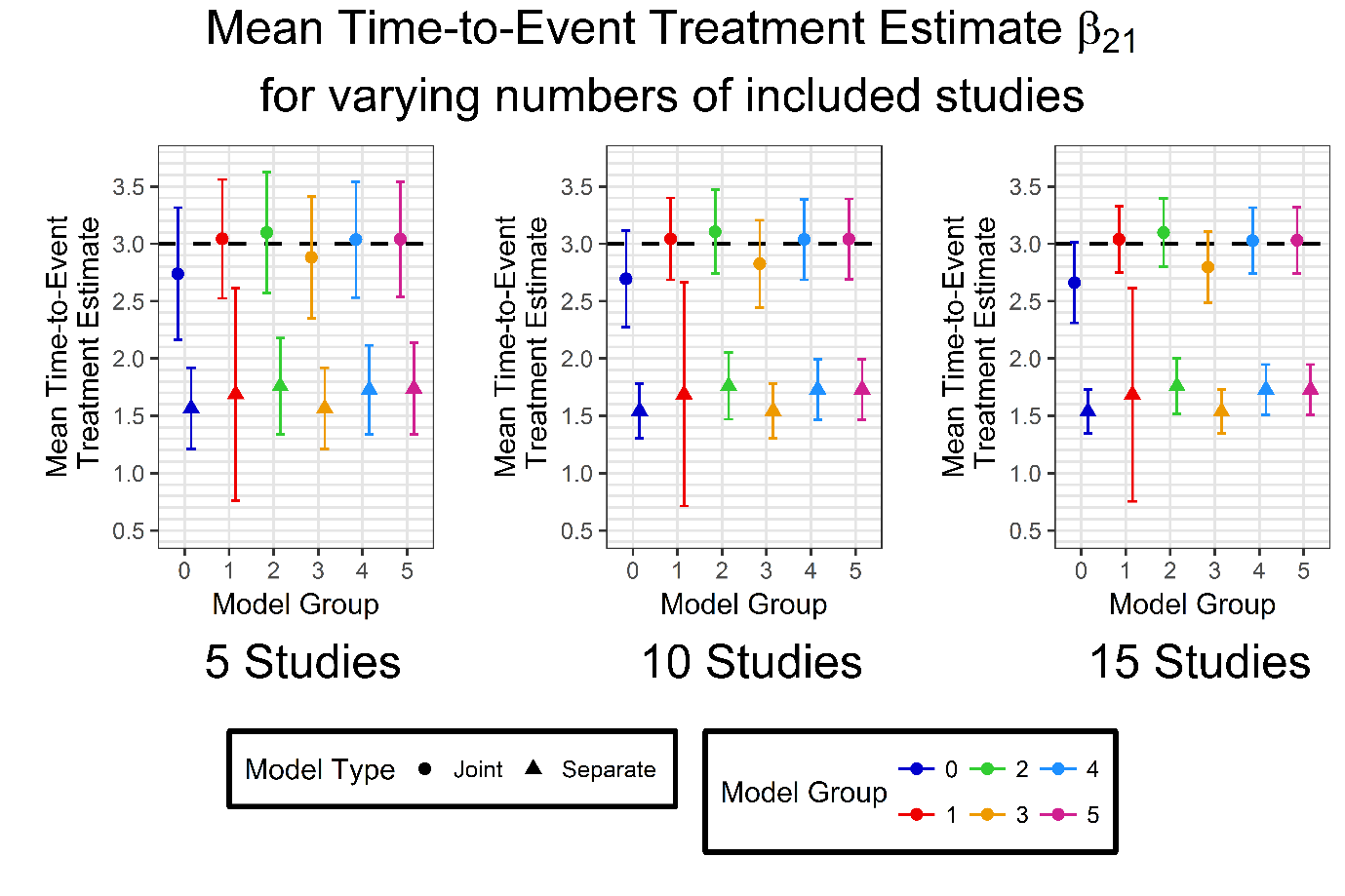


Supplemental Figure S30: Graphical representation of time-to-event treatment effect ($\beta_{21}$) estimates shown in Table 8 for simulation set 2: investigation of varying numbers of included studies. The dashed line indicates the “true” value of $\beta_{21}$ that the data was simulated under


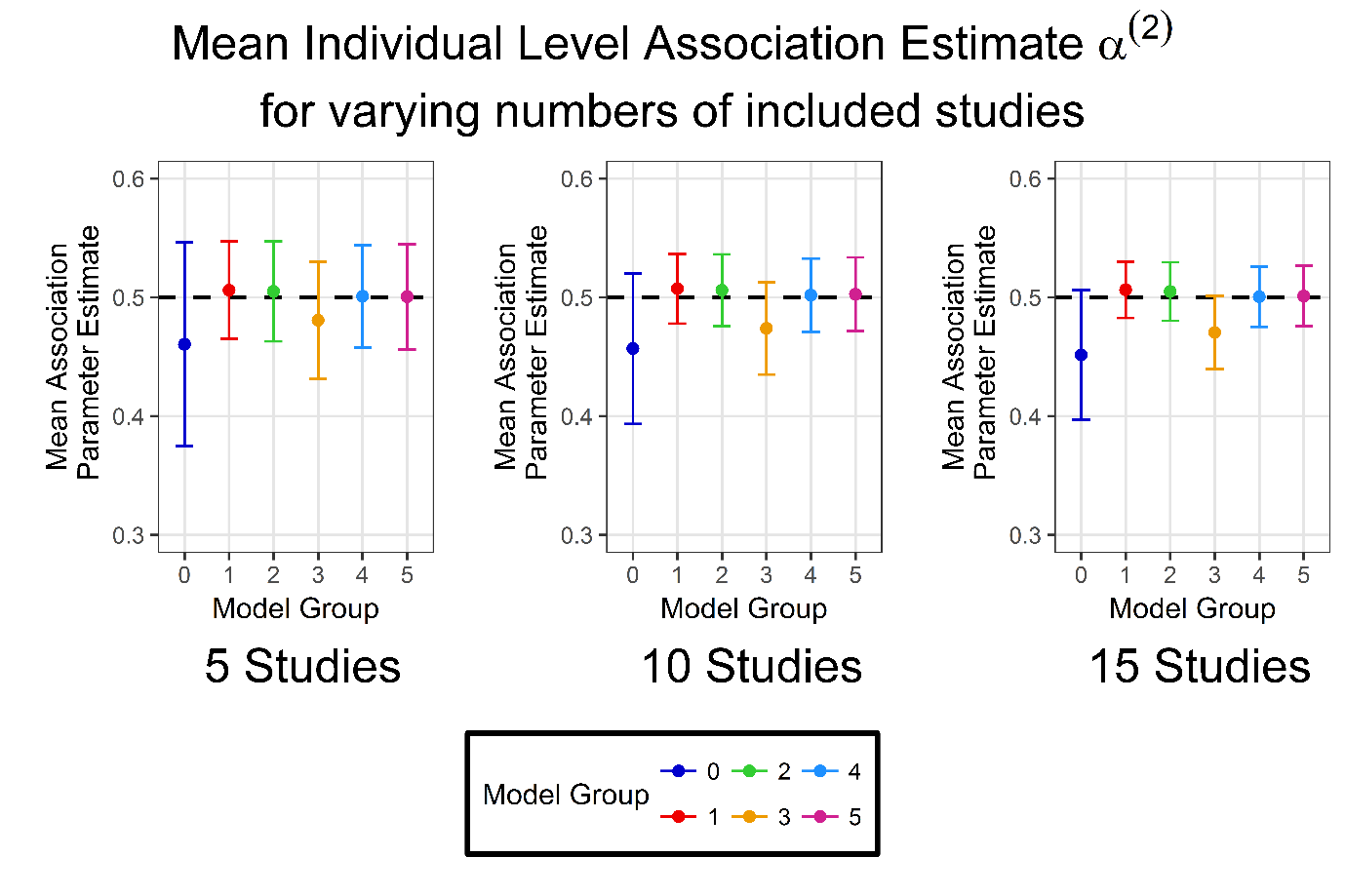


Supplemental Figure S31: Graphical representation of individual level association parameter ($\alpha^{\left( 2 \right)}$) estimates shown in Table 8 for simulation set 2: investigation of varying numbers of included studies. The dashed line indicates the “true” value of $\alpha^{\left( 2 \right)}$ that the data was simulated under


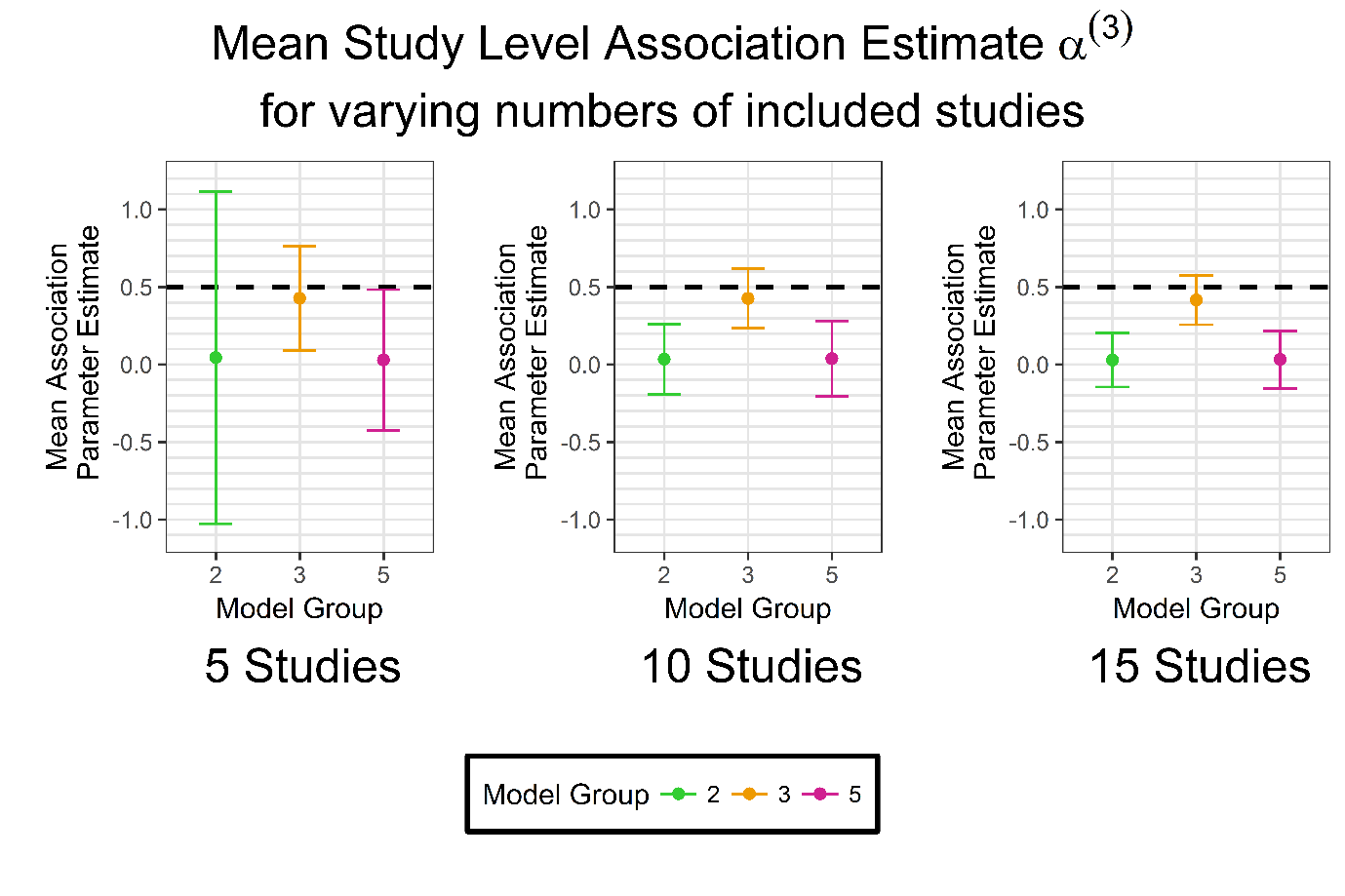


Supplemental Figure S32: Graphical representation of study level association parameter ($\alpha^{\left( 3 \right)}$) estimates shown in Table 8 for simulation set 2: investigation of varying numbers of included studies. The dashed line indicates the “true” value of $\alpha^{\left( 3 \right)}$ that the data was simulated under

## Graphical representation of Table 8 (point estimates)


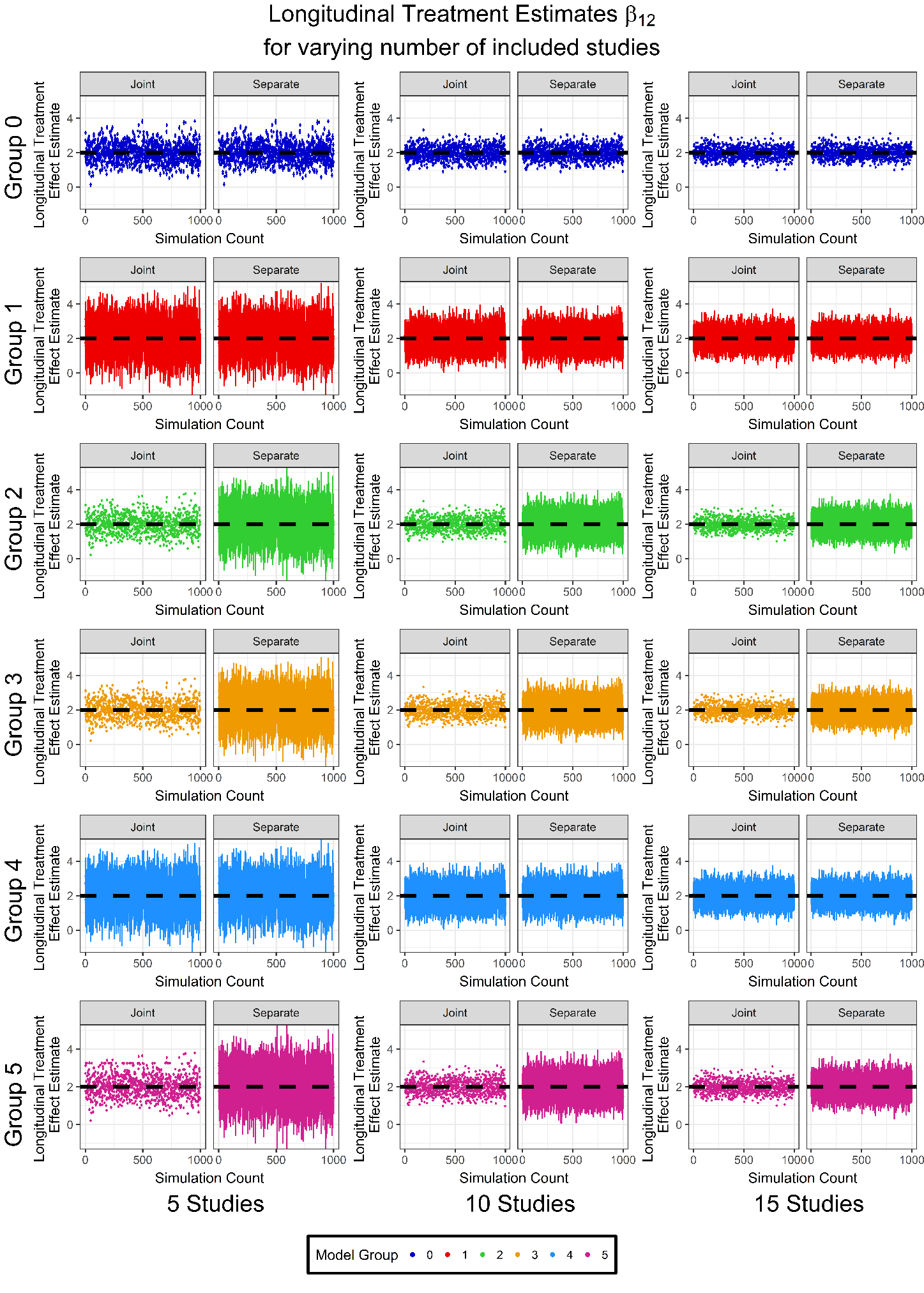


Supplemental Figure S33: Point estimates and confidence intervals for longitudinal treatment effect ($\beta_{12}$) for simulation group 2 investigating varying number of included studies. The dashed line indicates the value of $\beta_{12}$ that the data was simulated under


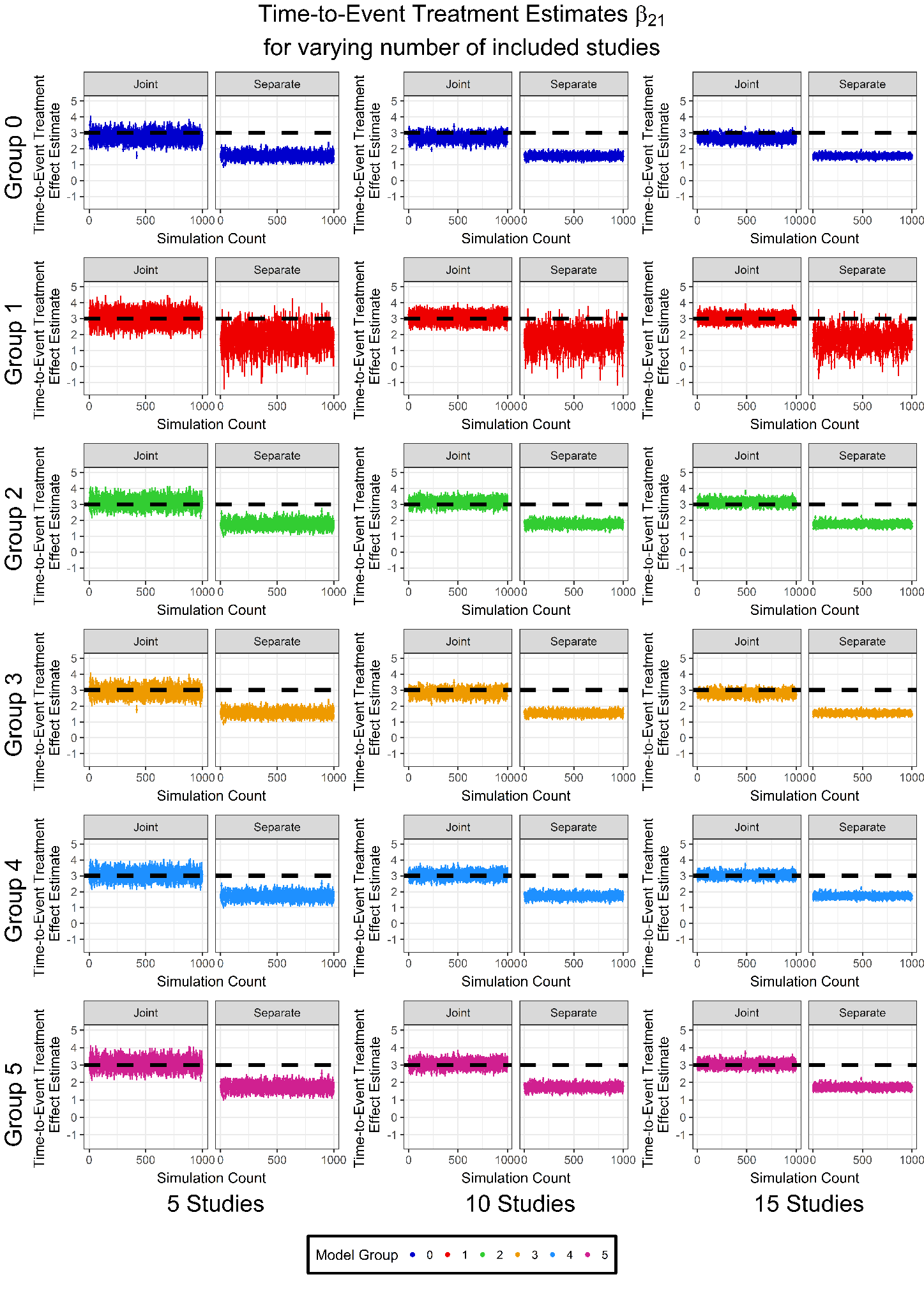


Supplemental Figure S34: Point estimates and confidence intervals for time-to-event treatment effect ($\beta_{21}$) for simulation group 2 investigating varying number of included studies. The dashed line indicates the value of $\beta_{21}$ that the data was simulated under


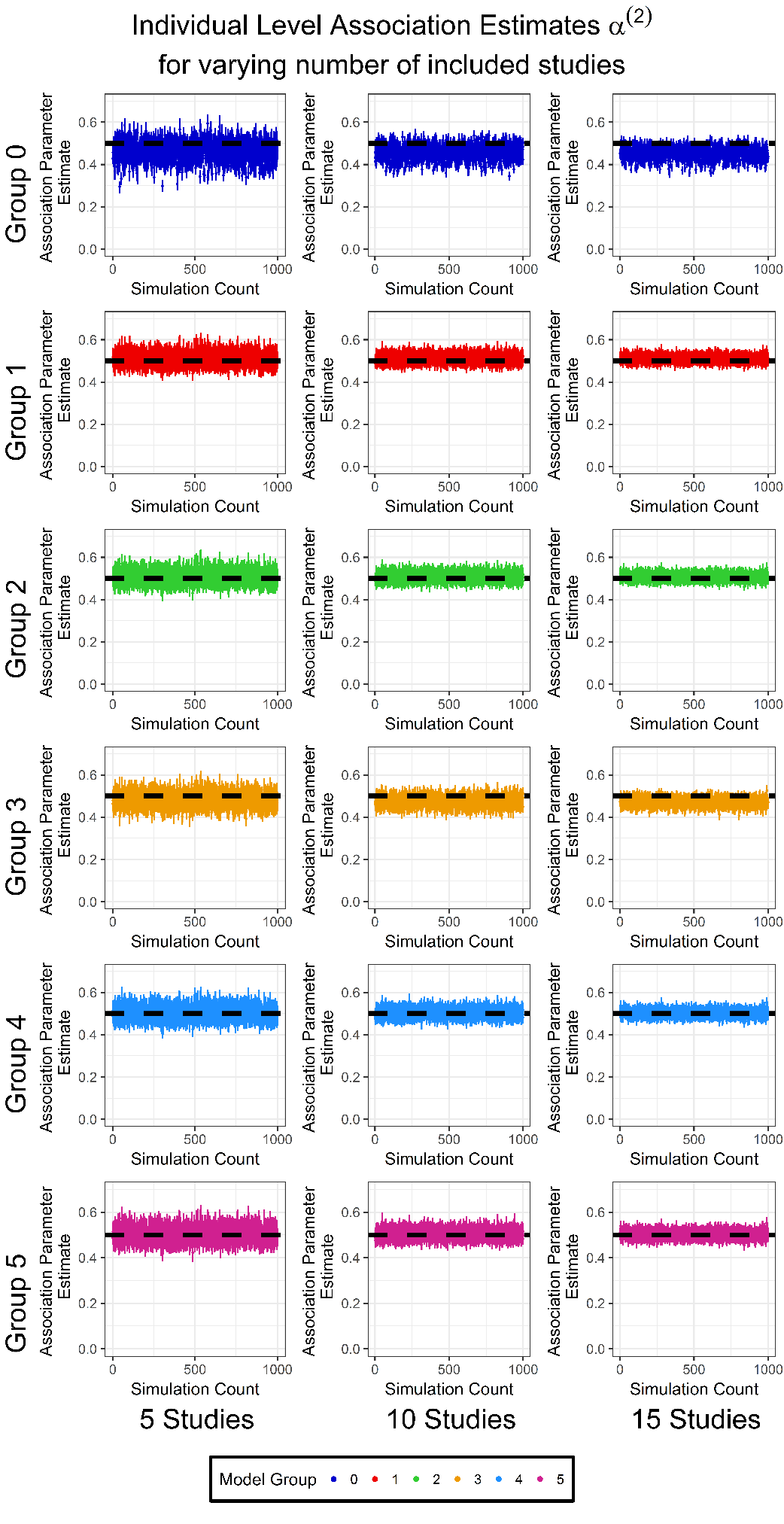


Supplemental Figure S35: Point estimates and confidence intervals for individual level association ($\alpha^{\left( 2 \right)}$) for simulation group 2 investigating varying number of included studies. The dashed line indicates the value of $\alpha^{\left( 2 \right)}$ that the data was simulated under


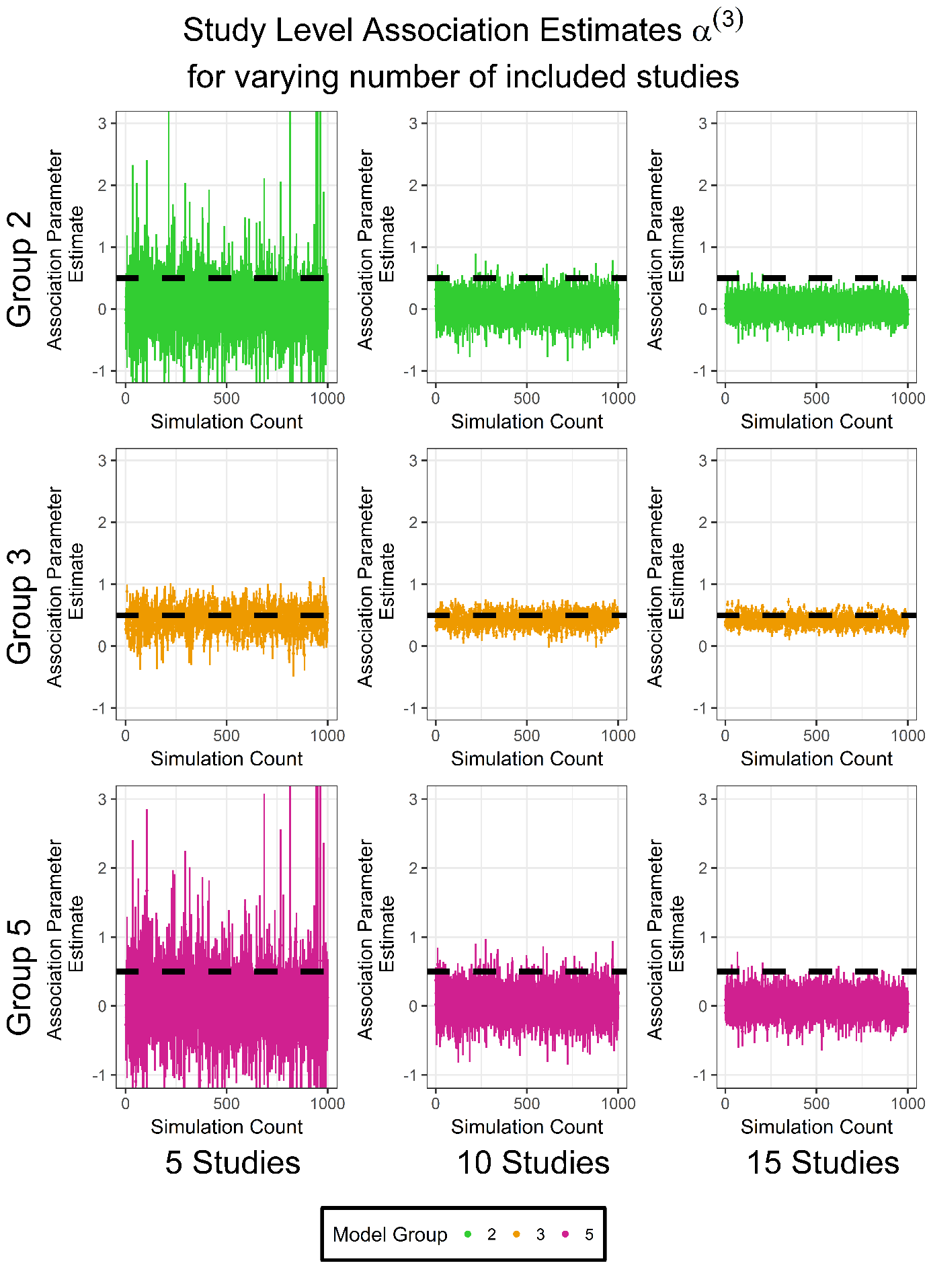


Supplemental Figure S36: Point estimates and confidence intervals for study level association ($\alpha^{\left( 3 \right)}$) for simulation group 2 investigating varying number of included studies. The dashed line indicates the value of $\alpha^{\left( 3 \right)}$ that the data was simulated under

# Graphical representations of results from Simulation Set 3: Varying levels of between study heterogeneity

## Graphical representation of Table 9 (mean estimates)


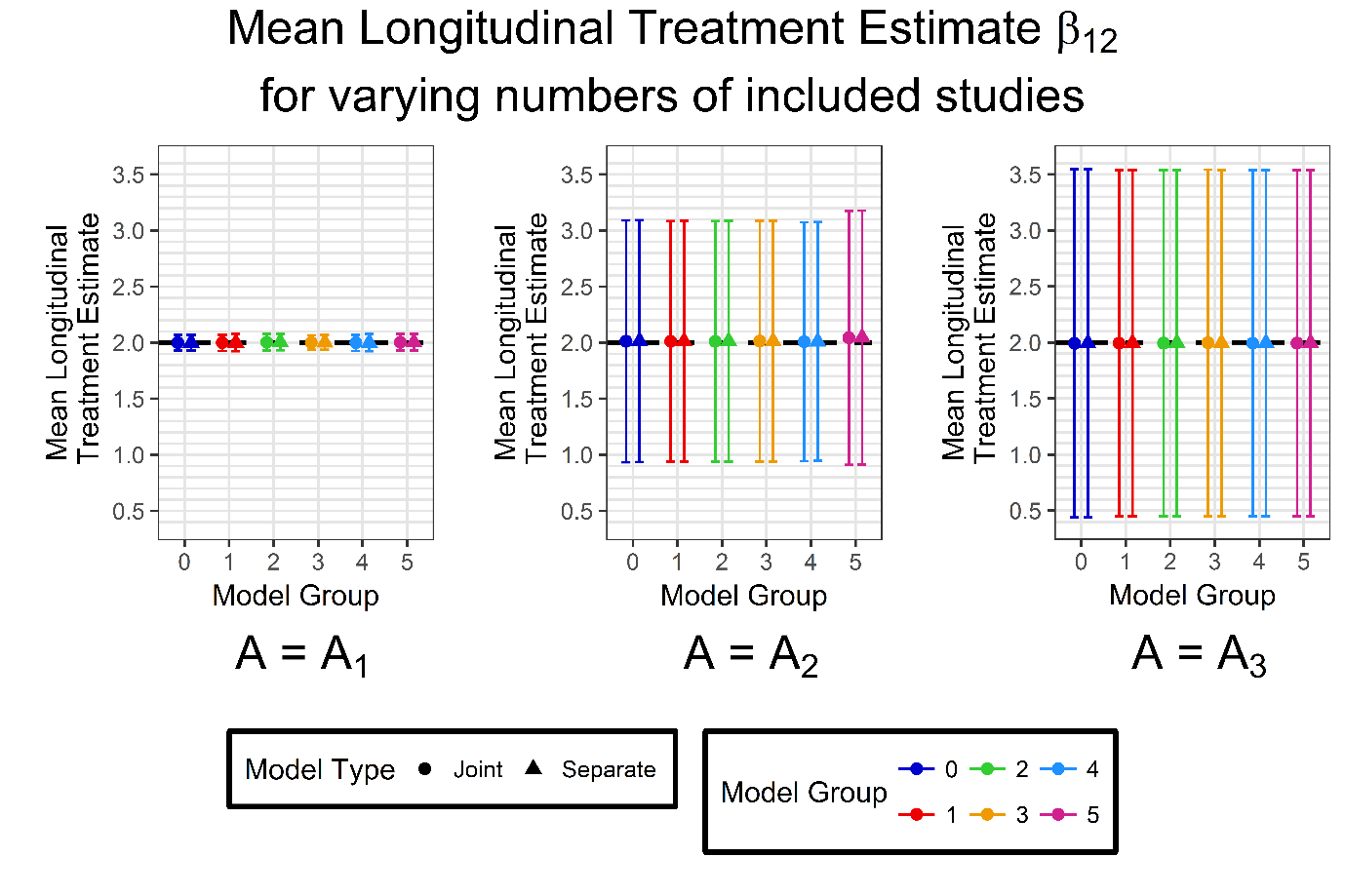


Supplemental Figure S37: Graphical representation of longitudinal treatment effect ($\beta_{12}$) estimates shown in Table 9 for simulation set 3: investigation of varying between study heterogeneity. The dashed line identifies the “true” value $\beta_{12}$ that the data was simulated under


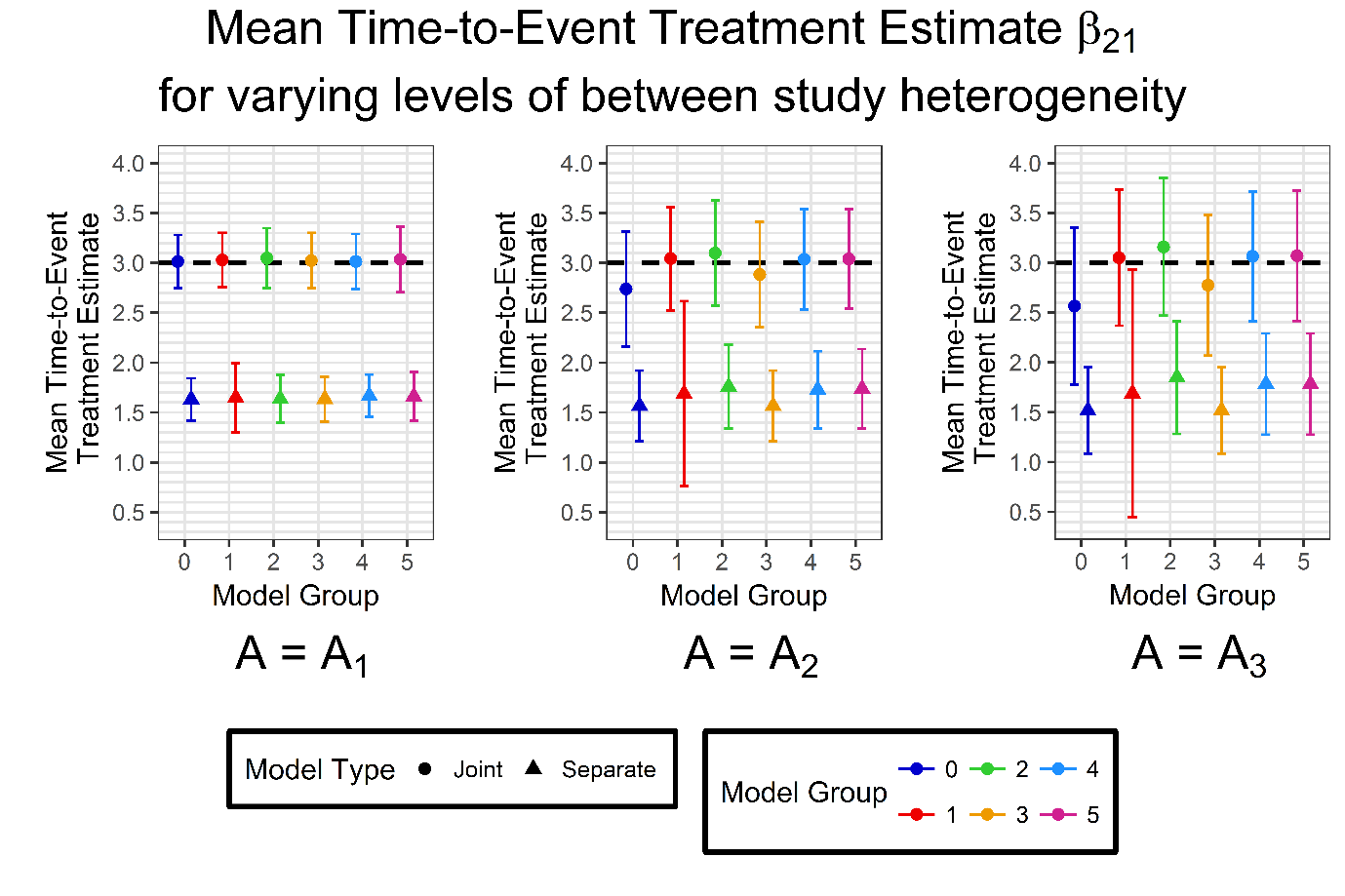


Supplemental Figure S38: Graphical representation of time-to-event treatment effect ($\beta_{21}$) estimates shown in Table 9 for simulation set 3: investigation of varying between study heterogeneity. The dashed line identifies the “true” value of $\beta_{21}$ that the data was simulated under


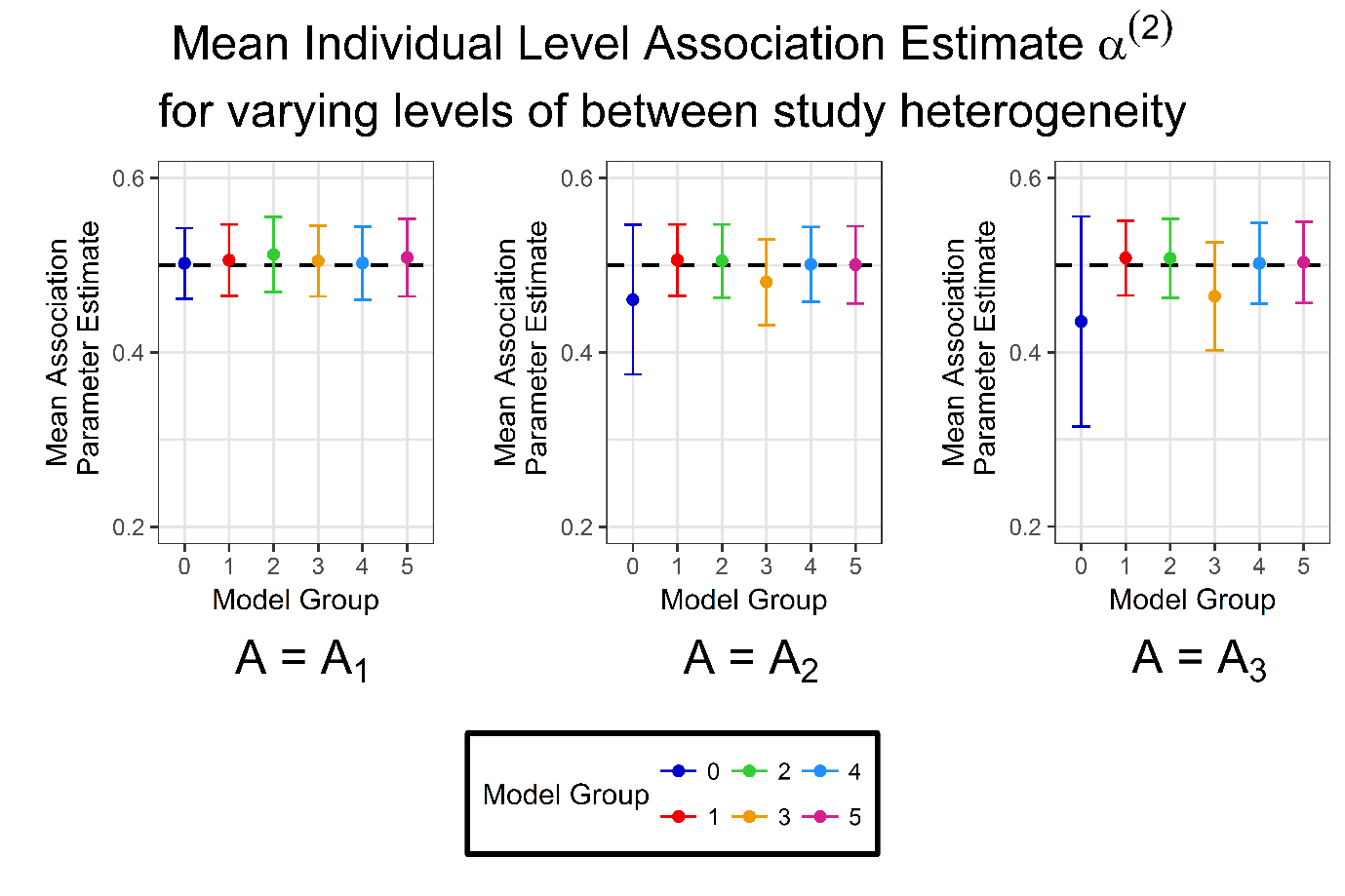


Supplemental Figure S39: Graphical representation of individual level association parameter ($\alpha^{\left( 2 \right)}$) estimates shown in Table 9 for simulation set 3: investigation of varying between study heterogeneity. The dashed line identifies the "true” value of $\alpha^{\left( 2 \right)}$that the data was simulated under


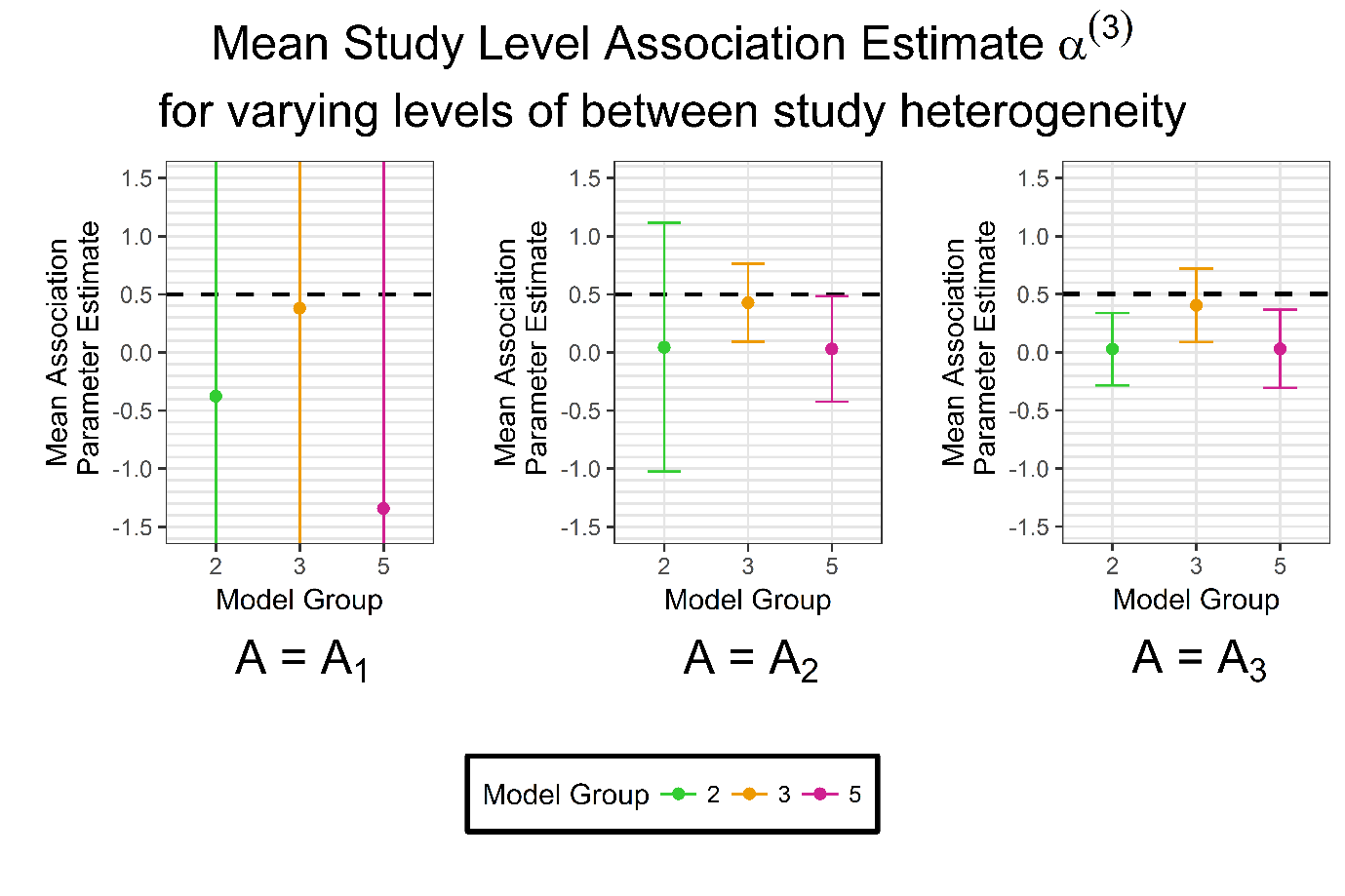


Supplemental Figure S40: Graphical representation of study level association parameter ($\alpha^{\left( 3 \right)}$) estimates shown in Table 9 for simulation set 3: investigation of varying between study heterogeneity. The dashed line identifies the “true” value of $\alpha^{\left( 3 \right)}$that the data was simulated under

## Graphical representation of Table 9 (points estimates)


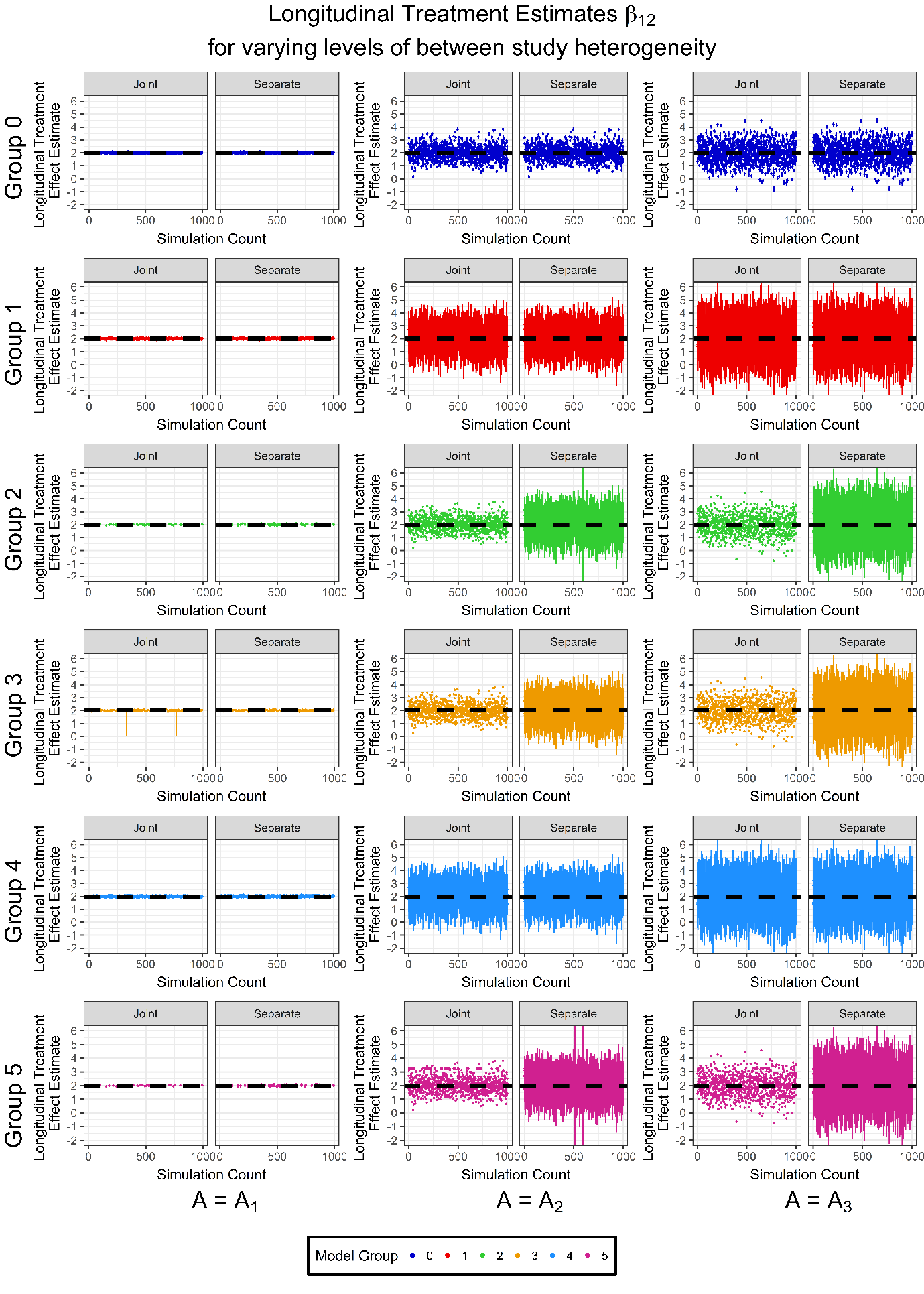


Supplemental Figure S41: Point estimates and confidence intervals for longitudinal treatment effect ($\beta_{12}$) parameter for simulation group 3 investigating varying between study heterogeneity. The dashed line identifies the value of $\beta_{12}$ that the data was simulated under


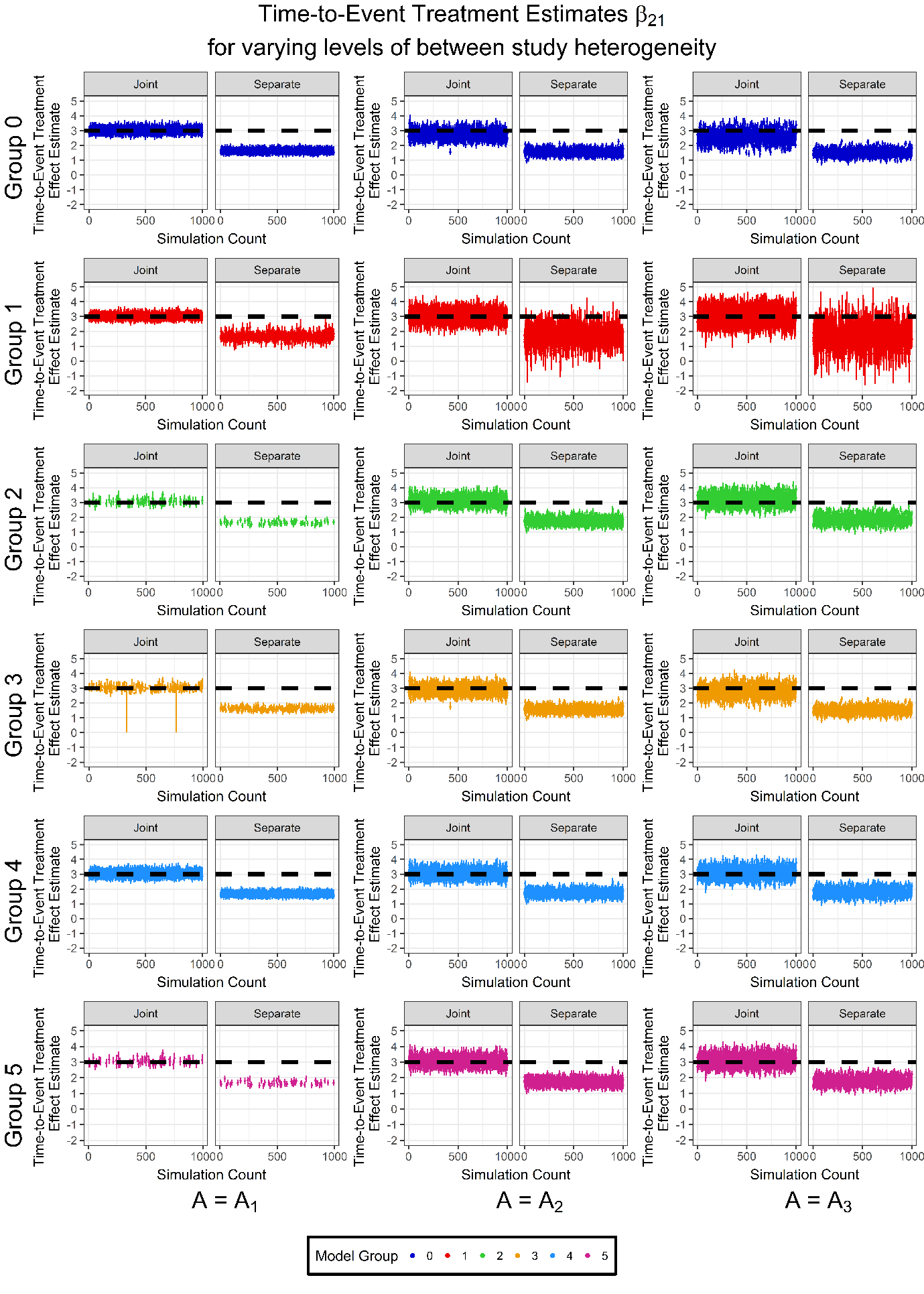


Supplemental Figure S42: Point estimates and confidence intervals for time-to-event treatment effect ($\beta_{21}$) parameter for simulation group 3 investigating varying between study heterogeneity. The dashed line identifies the value of $\beta_{21}$ that the data was simulated under.


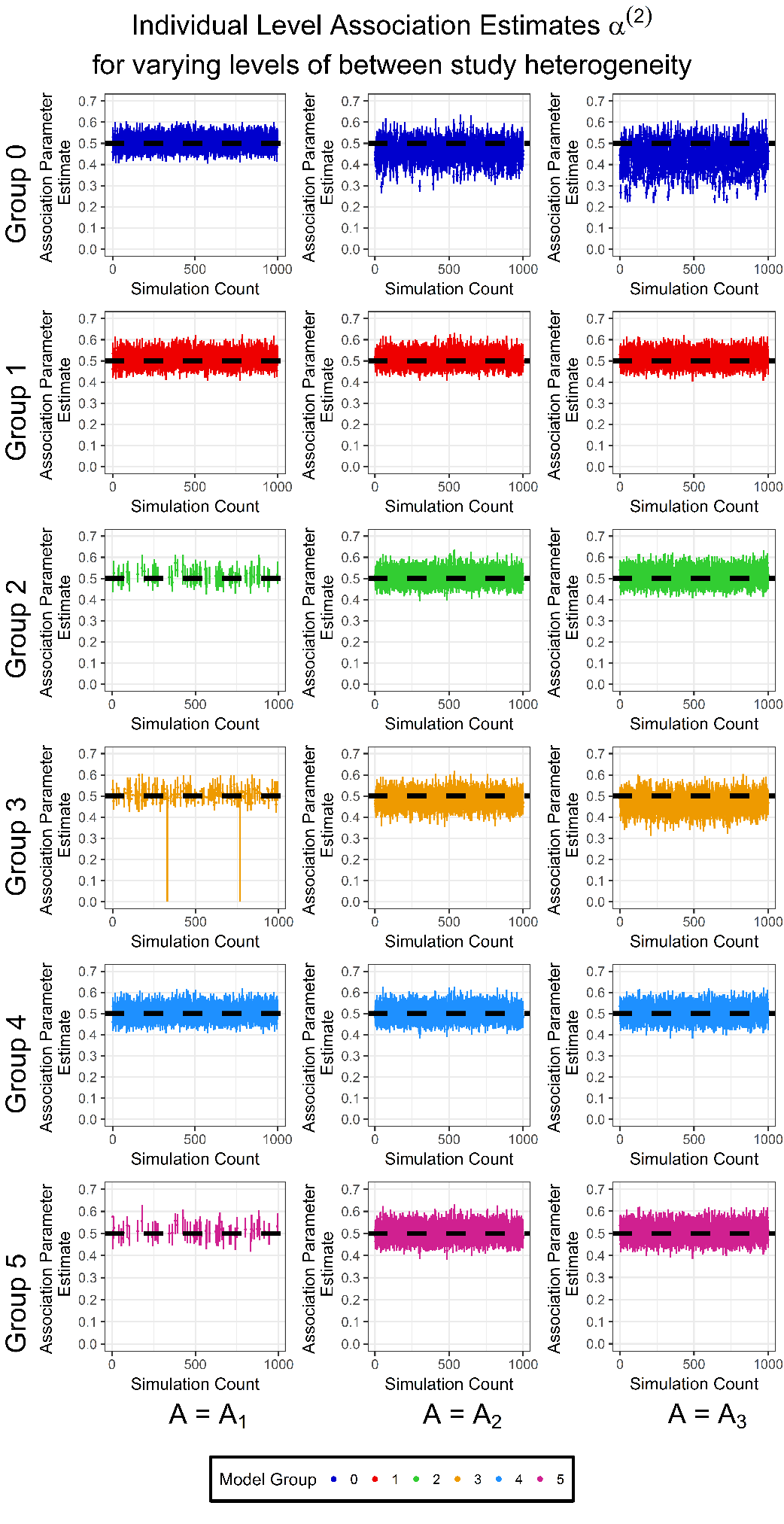


Supplemental Figure S43: Point estimates and confidence intervals for individual level association parameter ($\alpha^{\left( 2 \right)}$) parameter for simulation group 3 investigating varying between study heterogeneity. The dashed line identifies the value of $\alpha^{\left( 2 \right)}$that the data was simulated under.


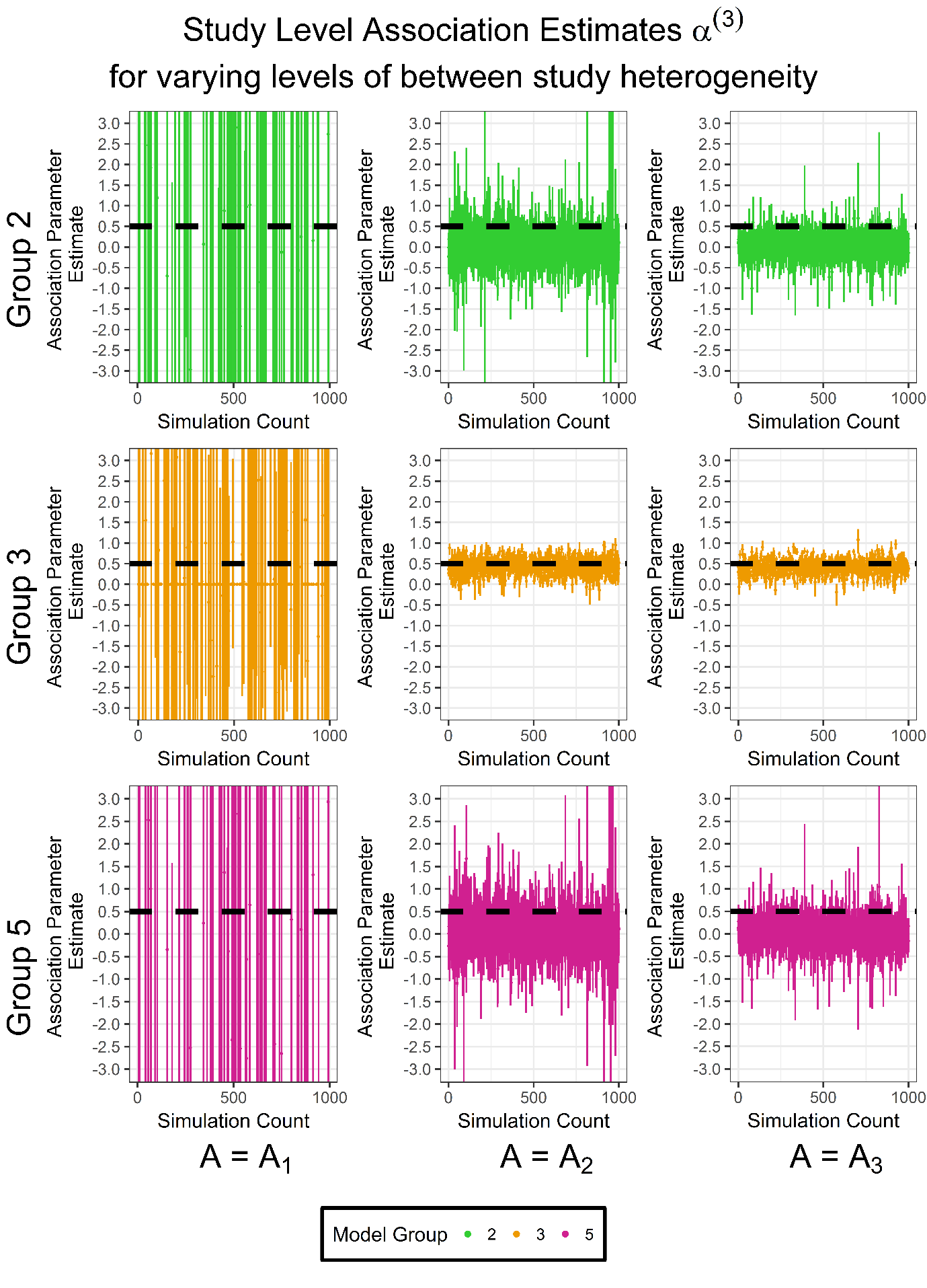


Supplemental Figure S44: Point estimates and confidence intervals for study level association parameter ($\alpha^{\left( 3 \right)}$) parameter for simulation group 3 investigating varying between study heterogeneity. The dashed line identifies that value of $\alpha^{\left( 3 \right)}$ that the data was simulated under
