# Supplementary material for: Investigation of one‐stage meta‐analysis methods for joint longitudinal and time‐to‐event data through simulation and real data application
Source: Stat Med. 2018 Sep 12;38(2):247–68. doi: 10.1002/sim.7961 (PMC6492085; doi:10.1002/sim.7961)
Supplement: Supplementary file 2 — SIM_7961‐Supp‐0002‐Supplemental Material ‐ Maximum Likelihood Estimators.docx [file SIM-38-247-s002.docx]

# Supplemental Material: Maximum Likelihood Estimators used in EM algorithm when fitting One-Stage Joint Meta-Analytic Models

During the Maximisation or M-step of the EM algorithm, parameter estimates were evaluated by calculating derivatives of the expectation of the complete log-likelihood conditional on the observed data with respect to each parameter estimate of interest. These derivatives were then set to zero and rearranged, giving maximum likelihood estimates for the parameters. This text is written with reference to the *joineRmeta* package in the R statistical software, which implements these methods to perform one-stage meta-analysis of joint data.

## General Likelihood formulation

The complete data is defined to be $\boldsymbol{\Omega}$, where $\boldsymbol{\Omega}=(T_{Ski}, \Delta_{ki}, \boldsymbol{Y}_{ki}, \boldsymbol{t}_{ki}, \boldsymbol{b}_{k}^{(3)},\boldsymbol{b}_{ki}^{(2)} )$, with $T_{Ski}$ representing the survival time for individual $i$ in study $k$, $\Delta_{ki}$ is the censoring indicator, $\boldsymbol{Y}_{ki}$ and $\boldsymbol{t}_{ki}$ are the vectors of longitudinal measurements and time points, while $\boldsymbol{b}_{k}^{(3)}$ and $\boldsymbol{b}_{ki}^{(2)}$ are the study and individual level random effects. The observed data is consists of the survival times $T_{Ski}$, the censoring indicator $\Delta_{ki}$, the longitudinal measurements $\boldsymbol{Y}_{ki}$, and the measurement times$\boldsymbol{t}_{ki}$. Equation (1) shows the complete or full likelihood (referred to as $L(\boldsymbol{\Omega})$) of the joint model for multiple studies.

In equation (1), $K$ is the total number of included studies (with study indicator $k=1\ldots K$). The total number of individuals in study $k$ is denoted by $n_{k}$. The total number of longitudinal measurements recorded for individual $i$ from study $k$ is denoted by $m_{ki}$. Definitions for each of the functions that contribute to $L\left( \boldsymbol{\Omega} \right)$ follow:

| $\prod_{k=1}^{K} \left( \int_{-\infty}^{\infty} f\left( \boldsymbol{b}_{k}^{(3)} \vert\boldsymbol{A} \right)\prod_{i=1}^{n_{k}} \left( \int_{-\infty}^{\infty} \left( \prod_{j=1}^{m_{ki}} f\left( Y_{kij} \vert\boldsymbol{\beta}_{1},\boldsymbol{b}_{ki}^{\left( 2 \right)},\boldsymbol{b}_{k}^{\left( 3 \right)},\sigma_{e}^{2} \right) \right)f\left( \boldsymbol{b}_{ki}^{(2)} \vert\boldsymbol{D} \right)f(T_{Ski},\Delta_{ki}\vert\boldsymbol{\beta}_{2},\boldsymbol{b}_{ki}^{(2)},\boldsymbol{b}_{k}^{(3)},\lambda_{0},\boldsymbol{\alpha})d\boldsymbol{b}_{ki}^{(2)} \right)d\boldsymbol{b}_{k}^{(3)} \right)$ | (1) |
| --- | --- |
| $f\left( Y_{kij} \vert\boldsymbol{\beta}_{1},\boldsymbol{b}_{ki}^{(2)},\boldsymbol{b}_{k}^{(3)},\sigma_{e}^{2} \right)={(2\pi\sigma_{e}^{2})}^{-1/2}\exp\left( {-{(Y_{kij}-\eta_{kij})}^{2}}/{2\sigma_{e}^{2}} \right)$ | (2) |
| $f\left( T_{Ski},\Delta_{ki} \vert\boldsymbol{\beta}_{\boldsymbol{2}}\boldsymbol{,}\boldsymbol{b}_{ki}^{(2)},\boldsymbol{b}_{k}^{(3)},\lambda_{0},\boldsymbol{\alpha} \right)=\left[ \lambda_{0}\left( T_{Ski} \right)\exp\left( \left( X_{2ki}\boldsymbol{\beta}_{\boldsymbol{2}} \right)\boldsymbol{+}\alpha^{(2)}\left( Z_{ki}^{(2)}\boldsymbol{b}_{ki}^{(2)} \right)+\alpha^{(3)}\left( Z_{ki}^{(3)}\boldsymbol{b}_{k}^{(3)} \right) \right) \right]^{\Delta_{ki}}$ $\exp\left[ -\int_{0}^{T_{Ski}} \lambda_{0}\left( u \right)\exp\left( \left( X_{2ki}\boldsymbol{\beta}_{\boldsymbol{2}} \right)\boldsymbol{+}\alpha^{(2)}\left( Z_{ki}^{(2)}\boldsymbol{b}_{ki}^{(2)} \right)+\alpha^{(3)}\left( Z_{ki}^{(3)}\boldsymbol{b}_{k}^{(3)} \right) \right)du \right]$ | (3) |
| $f\left( \boldsymbol{b}_{k}^{(3)} \vert\boldsymbol{A} \right)={{(2\pi)}^{-r/2}\left\vert\boldsymbol{A} \right\vert}^{-1/2}\exp\left\{ -\frac{\left( \boldsymbol{b}_{\boldsymbol{k}}^{\left( \boldsymbol{3} \right)} \right)^{T}\boldsymbol{A}^{-1}\left( \boldsymbol{b}_{\boldsymbol{k}}^{\left( \boldsymbol{3} \right)} \right)}{2} \right\}$ | (4) |
| $f\left( \boldsymbol{b}_{ki}^{(2)} \vert\boldsymbol{D} \right)={{(2\pi)}^{-q/2}\left\vert\boldsymbol{D} \right\vert}^{-1/2}exp\left\{ -\frac{\left( \boldsymbol{b}_{\boldsymbol{ki}}^{\left( \boldsymbol{2} \right)} \right)^{T}\boldsymbol{D}^{-1}\left( \boldsymbol{b}_{\boldsymbol{ki}}^{\left( \boldsymbol{2} \right)} \right)}{2} \right\}$ | (5) |

### Longitudinal sub-model component

The probability distribution function of each longitudinal measurement $Y_{kij}$ (recorded for individual $i$ in study $k$ at their $j$th recorded time-point $t_{kij}$) is given by $f\left( Y_{kij} | \boldsymbol{\beta}_{1},\boldsymbol{b}_{ki}^{(2)},\boldsymbol{b}_{k}^{(3)},\sigma_{e}^{2} \right)$, see equation (2) for the full specification. The total contribution of the longitudinal portion to the complete likelihood for a particular individual is found by the product of this function across all time points recorded for the individual in question, i.e.$\prod_{j=1}^{m_{ki}} f\left( Y_{kij} | \boldsymbol{\beta}_{1},\boldsymbol{b}_{ki}^{(2)},\boldsymbol{b}_{k}^{(3)},\sigma_{e}^{2} \right)$. Here, $\boldsymbol{\beta}_{1}$ are the longitudinal population fixed effects, $\boldsymbol{b}_{ki}^{(2)}$ are the individual specific (level 2) random effects, $\boldsymbol{b}_{k}^{(3)}$ are the study specific (level 3) random effects (if present), and $\sigma_{e}^{2}$ is the variance of the measurement errors (represented by $\varepsilon_{kij}$). These longitudinal measurements can occur at times unique to each individual. This vector of measurement times for each individual is denoted by $\boldsymbol{t}_{ki}$ and is of length $m_{ki}$. It is assumed that the longitudinal measure recorded at each time point for an individual can be considered normally distributed. The mean of this normal distribution is the sum of the fixed ($\boldsymbol{x}_{\boldsymbol{1}\boldsymbol{kij}}\boldsymbol{\beta}_{\boldsymbol{1}}$) and random components ($\boldsymbol{z}_{\boldsymbol{kij}}^{\boldsymbol{(2)}}\boldsymbol{b}_{\boldsymbol{ki}}^{\boldsymbol{(2)}}$ and $\boldsymbol{z}_{\boldsymbol{kij}}^{\boldsymbol{(3)}}\boldsymbol{b}_{\boldsymbol{k}}^{\boldsymbol{(3)}}$) of the longitudinal model (represented by $\eta_{kij}$ where $\eta_{kij}=\boldsymbol{x}_{\boldsymbol{1}\boldsymbol{kij}}\boldsymbol{\beta}_{\boldsymbol{1}}\boldsymbol{+}\boldsymbol{z}_{\boldsymbol{kij}}^{\boldsymbol{(2)}}\boldsymbol{b}_{\boldsymbol{ki}}^{\boldsymbol{(2)}}+\boldsymbol{z}_{\boldsymbol{kij}}^{\boldsymbol{(3)}}\boldsymbol{b}_{\boldsymbol{k}}^{\boldsymbol{(3)}}$), with variance equal to $\sigma_{e}^{2}$. Note, that in the Maximisation or M-step of the Expectation Maximisation (EM) algorithm (the procedure employed in the jointmeta1 function of the joineRmeta package to fit the one-stage joint model) the random effects contribute to the mean rather than the variance of the longitudinal measurements. This is because in the Expectation or E-step, estimates of functions of the random effects are produced. In the M-step, the likelihood of the model is maximized, with functions of the random effects held constant at the estimates produced in the E-step (resulting in their contribution to the mean rather than the variance of the longitudinal measurement distribution).

In $\eta_{kij}$, $\boldsymbol{x}_{\boldsymbol{1}\boldsymbol{kij}}$ represents the covariates for the fixed effects at time point $j$ for individual $i$ in study $k$. The covariates over all time points for the individual are held in the design matrix $\boldsymbol{X}_{\boldsymbol{1}\boldsymbol{ki}}$, which will have $m_{ki}$ rows and $p_{1}$ columns (where $p_{1}$ is the number of fixed effects in the longitudinal sub-model).

The covariates for the individual specific (level 2) random effects at time point $j$ for individual $i$ in study $k$ are represented by $\boldsymbol{z}_{\boldsymbol{kij}}^{\boldsymbol{(2)}}$, and those for the study level random effects by $\boldsymbol{z}_{\boldsymbol{kij}}^{\boldsymbol{(3)}}$. The covariates over all time points are held in design matrices $\boldsymbol{Z}_{\boldsymbol{ki}}^{\boldsymbol{(2)}}$ and $\boldsymbol{Z}_{\boldsymbol{ki}}^{\boldsymbol{(3)}}$ respectively. The random effects themselves are represented by $\boldsymbol{b}_{ki}^{(2)}$ and $\boldsymbol{b}_{k}^{(3)}$ for the individual and study level random effects respectively. If study level random effects are not included in the model, terms involving $\boldsymbol{b}_{k}^{(3)}$ are not present. The model assumes that the covariates assigned random effects have also been assigned fixed effects (i.e. that the columns forming design matrices $\boldsymbol{Z}_{\boldsymbol{ki}}^{\boldsymbol{(2)}}$ and $\boldsymbol{Z}_{\boldsymbol{ki}}^{\boldsymbol{(3)}}$ are subsets of the columns of design matrix $\boldsymbol{X}_{\boldsymbol{1}\boldsymbol{ki}}$).

Throughout, only time varying covariates that can be expressed as functions of time (e.g. interactions between time and stationary variables, such as treatment group) can be assigned random effects in the longitudinal sub-model. Other time varying covariates that cannot be stated as some function of time and a stationary covariate are not allowed to be assigned random effects (e.g. weight measured at successive time points). This is due to the necessity to know the value of the random effects at $T_{Ski}$ (the survival time of individual $i$ in study $k$); currently the value in the association structure for a time-varying covariate not calculable from a time variable and a stationary covariate cannot be approximated by the package.

### Time-to-event sub-model component

The probability distribution function of the time-to-event component is represented by $f(T_{Ski},\Delta_{ki}|\boldsymbol{\beta}_{2},\boldsymbol{b}_{ki}^{(2)},\boldsymbol{b}_{k}^{(3)},\lambda_{0},\boldsymbol{\alpha})$, as specified in equation (3). The exact structure of this component depends on the association structure of the joint model. Only the random effects proportional association structure is currently considered.

Throughout, $\boldsymbol{X}_{\boldsymbol{2}}$ represents the design matrix for the fixed effects in the time-to-event sub-model, and $\boldsymbol{\beta}_{\boldsymbol{2}}$ the fixed effect coefficients, while $\lambda_{0}$ represents the unspecified baseline hazard function. The design matrix $\boldsymbol{X}_{\boldsymbol{2}}$ will have number of rows equal to the number of individuals in the analysis (total number of individuals across studies for non-stratified models ($\sum_{k=1}^{K} n_{k}$), or number of individuals within a study ($n_{k}$) for models with a stratified baseline, in which case the fixed effect design matrix would be denoted $\boldsymbol{X}_{\boldsymbol{2}\boldsymbol{k}}$), and will always have $p_{2}$ columns. Here $p_{2}$ is the number of fixed effects in the time-to-event sub-model. It is assumed that $\boldsymbol{X}_{\boldsymbol{2}}$ contains no time variable covariates.

The survival time${(T}_{Ski})$ is the minimum of the true event time $(T_{Eki})$ and the censoring time$(T_{Cki})$ for individual $i$ in study $k$. The event indicator ($\Delta_{ki}$) takes a value of 1 if the individual experienced an event at $T_{Ski}$, and 0 otherwise. Terms shared between the sub-models (preceded by $\boldsymbol{\alpha}$ terms) have the same definitions as in the longitudinal sub-model section. Note that any point where time is used in the shared terms in $f\left( T_{Ski},\Delta_{ki} | \boldsymbol{\beta}_{\boldsymbol{2}},\boldsymbol{b}_{ki}^{(2)},\boldsymbol{b}_{k}^{(3)},\lambda_{0},\boldsymbol{\alpha} \right)$, $T_{Ski}$ (or times from 0 to $T_{Ski}$) are used in place of longitudinal times $t_{kij}$. Similarly any time varying covariates in the shared terms take values at times relating to the survival data rather than the longitudinal data.

The association terms (represented by $\boldsymbol{\alpha}$ terms) have bracketed superscripts to identify the data level they relate to. Specifically, $\alpha^{(2)}$ denotes the association parameter for shared zero mean individual level (level 2) random effects, and $\alpha^{\left( 3 \right)}$ represents the association parameter for shared zero mean study level (level 3) random effects. This function can currently only fit models that share zero mean random effects between sub-models, with common association parameter across random effects at the same level, termed random effects only proportional association.

As before, if no study level random effects are included in the model the $\alpha^{(3)}\left( \boldsymbol{Z}_{\boldsymbol{ki}}^{\boldsymbol{(3)}}\boldsymbol{b}_{\boldsymbol{k}}^{\boldsymbol{(3)}} \right)$ component is not present.

### Study specific random effects component

The probability distribution of the zero mean study specific (level 3) random effects, $f\left( \boldsymbol{b}_{k}^{\left( 3 \right)}|\boldsymbol{A} \right)$, is given in equation (4). If no study level random effects are included in the model, then $f\left( \boldsymbol{b}_{k}^{\left( 3 \right)}|\boldsymbol{A} \right)$ is not present in equation (1) (and consequently $\boldsymbol{b}_{k}^{(3)}$ does not require integrating out). The random effects are considered to follow a zero mean multivariate normal distribution with covariance matrix $\boldsymbol{A}$ of dimension$r$ (the number of study level random effects).

If only one study specific random effect is considered, giving $\boldsymbol{A}=\sigma_{A}^{2}$ and $r=1$, then equation (4) becomes a univariate distribution:

|  | $f\left( \boldsymbol{b}_{k}^{(3)} \vert\sigma_{A}^{2} \right)=\left( 2\pi\sigma_{A}^{2} \right)^{-1/2}exp\left\{ {{-\left( \boldsymbol{b}_{\boldsymbol{k}}^{\boldsymbol{(3)}} \right)}^{2}}/{2\sigma_{A}^{2}} \right\}$ |  |
| --- | --- | --- |

### Individual level random effects component

The probability distribution function of the zero mean individual specific (level 2) random effects is given by $f\left( \boldsymbol{b}_{ki}^{(2)} | \boldsymbol{D} \right)$, see equation (5), where $q$ represents the number of individual specific (level 2) random effects. As with the study level random effects, the individual level random effects are assumed to follow a multivariate normal distribution with covariance matrix $\boldsymbol{D}$ if $q>1$:

If only one individual level random effect is included in the model, then $\boldsymbol{D=}\sigma_{D}^{2}$ and $q=1$, then equation (5) becomes a univariate distribution:

|  | $f\left( \boldsymbol{b}_{ki}^{(2)} \vert\sigma_{D}^{2} \right)=\left( 2\pi\sigma_{D}^{2} \right)^{-1/2}exp\left\{ {{-\left( \boldsymbol{b}_{ki}^{(2)} \right)}^{2}}/{2\sigma_{D}^{2}} \right\}$ |  |
| --- | --- | --- |

The *jointmeta1()* function assumes that all models fitted will have at least one individual level random effect, and so $f\left( \boldsymbol{b}_{ki}^{(2)} | \boldsymbol{D} \right)$ will always be present in the likelihood.

## Maximum Likelihood Estimators

### Estimation of the unspecified baseline hazard

Wulfsohn and Tsiatis [78] note that the baseline hazard function is only considered to take weight at an event time. This is in line with the discussion that follows the main text in Cox [50]. Additionally, the closed form estimate of the baseline hazard function in Wulfsohn and Tsiatis [78] takes the form of the Breslow estimator for the baseline hazard (see Breslow, part of the discussion of Cox [50]). The estimators in the models fitted by *joineRmeta* take a similar form, based on the Breslow estimator, but summed across all the included studies:

| $\hat{\lambda}_{0}\left( u_{g} \right)=\sum_{k=1}^{K} \sum_{i=1}^{n_{k}} \frac{\Delta_{ki}\mathbb{I}\left( T_{Ski}=u_{g} \right)}{\sum_{h\in R(u_{g})} \exp\left( X_{2h}\boldsymbol{\beta}_{\boldsymbol{2}} \right)\mathbb{E}\left[ \exp\left( \alpha^{(2)}\left( Z_{h}^{(2)}\boldsymbol{b}_{h}^{(2)} \right) \right) \right]\mathbb{E}\left[ \exp\left( \alpha^{(3)}\left( Z_{h}^{(3)}\boldsymbol{b}_{h}^{(3)} \right) \right) \right]}$ | (6) |
| --- | --- |

In equation (6), $g$ represents a particular event time (where $g=1,\ldots,G$ is used to count through the unique event times). The indicator variable $\mathbb{I(}T_{Ski}=u_{g})$ ensures that the baseline hazard function only takes weight at times when there is an event or censoring, and multiplying it by the censoring indicator $\Delta_{ki}$ ensures weight is only taken event times. The risk set (individuals who have not yet been censored or have not yet experienced an event) at event time $u_{g}$ is denoted by $R(u_{g})$. For stratified models the risk set is drawn separately from each included study’s population, whereas for un-stratified models the risk set is drawn from all individuals in the meta-analysis. As such, for models with a stratified baseline hazard, a separate baseline hazard will be calculated for each study included in the meta-analysis. The expression in equation (6) is given for an un-stratified baseline hazard, however the expression can be simply modified to the stratified case by changing those who contribute to the risk set.

### Estimation of the longitudinal fixed effect coefficients

The longitudinal sub-model fixed effects ($\boldsymbol{\beta}_{\boldsymbol{1}}$) were estimated using the Ordinary Least Squares (OLS) estimator. This estimator is based on the residual sum of squares (RSS), denoted by:

|  | $\left( \boldsymbol{Y-}\boldsymbol{X}_{\boldsymbol{1}}\boldsymbol{\beta}_{\boldsymbol{1}}\mathbb{-E}\left[ \boldsymbol{Z}^{\left( \boldsymbol{2} \right)}\boldsymbol{b}^{\left( \boldsymbol{2} \right)} \right]\mathbb{-E}\left[ \boldsymbol{Z}^{\left( \boldsymbol{3} \right)}\boldsymbol{b}^{\left( \boldsymbol{3} \right)} \right] \right)^{T}\left( \boldsymbol{Y-}\boldsymbol{X}_{\boldsymbol{1}}\boldsymbol{\beta}_{\boldsymbol{1}}\mathbb{-E}\left[ \boldsymbol{Z}^{\left( \boldsymbol{2} \right)}\boldsymbol{b}^{\left( \boldsymbol{2} \right)} \right]\mathbb{-E}\left[ \boldsymbol{Z}^{\left( \boldsymbol{3} \right)}\boldsymbol{b}^{\left( \boldsymbol{3} \right)} \right] \right)$ $=\left( \left( \boldsymbol{Y-}\mathbb{E}\left[ \boldsymbol{Z}^{\left( \boldsymbol{2} \right)}\boldsymbol{b}^{\left( \boldsymbol{2} \right)} \right]\mathbb{-E}\left[ \boldsymbol{Z}^{\left( \boldsymbol{3} \right)}\boldsymbol{b}^{\left( \boldsymbol{3} \right)} \right] \right)\boldsymbol{-}\boldsymbol{X}_{\boldsymbol{1}}\boldsymbol{\beta}_{\boldsymbol{1}} \right)^{T}\left( \left( \boldsymbol{Y-}\mathbb{E}\left[ \boldsymbol{Z}^{\left( \boldsymbol{2} \right)}\boldsymbol{b}^{\left( \boldsymbol{2} \right)} \right]\mathbb{-E}\left[ \boldsymbol{Z}^{\left( \boldsymbol{3} \right)}\boldsymbol{b}^{\left( \boldsymbol{3} \right)} \right] \right)\boldsymbol{-}\boldsymbol{X}_{\boldsymbol{1}}\boldsymbol{\beta}_{\boldsymbol{1}} \right)$ $=\left( \boldsymbol{Y-}\mathbb{E}\left[ \boldsymbol{Z}^{\left( \boldsymbol{2} \right)}\boldsymbol{b}^{\left( \boldsymbol{2} \right)} \right]\mathbb{-E}\left[ \boldsymbol{Z}^{\left( \boldsymbol{3} \right)}\boldsymbol{b}^{\left( \boldsymbol{3} \right)} \right] \right)^{T}\left( \boldsymbol{Y-}\mathbb{E}\left[ \boldsymbol{Z}^{\left( \boldsymbol{2} \right)}\boldsymbol{b}^{\left( \boldsymbol{2} \right)} \right]\mathbb{-E}\left[ \boldsymbol{Z}^{\left( \boldsymbol{3} \right)}\boldsymbol{b}^{\left( \boldsymbol{3} \right)} \right] \right) -2\boldsymbol{\beta}_{\boldsymbol{1}}^{\boldsymbol{T}}\boldsymbol{X}_{\boldsymbol{1}}^{\boldsymbol{T}}\left( \boldsymbol{Y-}\mathbb{E}\left[ \boldsymbol{Z}^{\left( \boldsymbol{2} \right)}\boldsymbol{b}^{\left( \boldsymbol{2} \right)} \right]\mathbb{-E}\left[ \boldsymbol{Z}^{\left( \boldsymbol{3} \right)}\boldsymbol{b}^{\left( \boldsymbol{3} \right)} \right] \right)+\boldsymbol{\beta}_{\boldsymbol{1}}^{\boldsymbol{T}}\boldsymbol{X}_{\boldsymbol{1}}^{\boldsymbol{T}}\boldsymbol{X}_{\boldsymbol{1}}\boldsymbol{\beta}_{\boldsymbol{1}}$ | (7) |
| --- | --- | --- |

As population parameters are being estimated, common across all included studies, the estimation procedure involves all data in the dataset. The first derivative of this expression with respect to the fixed effect coefficients $\boldsymbol{\beta}_{\boldsymbol{1}}$ gives:

|  | $\frac{d}{d\boldsymbol{\beta}_{\boldsymbol{1}}}=-2\boldsymbol{X}_{\boldsymbol{1}}^{\boldsymbol{T}}\left( \boldsymbol{Y-}\mathbb{E}\left[ \boldsymbol{Z}^{\left( \boldsymbol{2} \right)}\boldsymbol{b}^{\left( \boldsymbol{2} \right)} \right]\mathbb{-E}\left[ \boldsymbol{Z}^{\left( \boldsymbol{3} \right)}\boldsymbol{b}^{\left( \boldsymbol{3} \right)} \right] \right)+\boldsymbol{2}\boldsymbol{X}_{\boldsymbol{1}}^{\boldsymbol{T}}\boldsymbol{X}_{\boldsymbol{1}}\boldsymbol{\beta}_{\boldsymbol{1}}$ | (8) |
| --- | --- | --- |

Setting this equal to zero, and rearranging, gives the ordinary least squares estimate of the longitudinal fixed effects:

|  | ${\hat{\boldsymbol{\beta}}}_{1}=\left( \boldsymbol{X}^{\boldsymbol{T}}\boldsymbol{X} \right)^{-1}\left( \boldsymbol{X}^{\boldsymbol{T}}\left( \boldsymbol{Y-}\mathbb{E}\left[ \boldsymbol{Z}^{\left( \boldsymbol{2} \right)}\boldsymbol{b}^{\left( \boldsymbol{2} \right)} \right]\mathbb{-E}\left[ \boldsymbol{Z}^{\left( \boldsymbol{3} \right)}\boldsymbol{b}^{\left( \boldsymbol{3} \right)} \right] \right) \right)$ | (9) |
| --- | --- | --- |

### Estimation of the time-to-event fixed effect coefficients and association parameters

The fixed effect coefficients ($\boldsymbol{\beta}_{\boldsymbol{2}}$) in the time-to-event sub-model and the association parameters ($\alpha^{\left( 2 \right)}$ and $\alpha^{\left( 3 \right)}$) will be estimated using a one-step Newton Raphson method (as is currently used in *joineR* [80] , and discussed by Rizopoulos [52]). To update the estimates of these parameters, the score vector $\boldsymbol{S}$ and the information matrix $\boldsymbol{I}$ must be calculated.

It should be highlighted that during model fitting, the association parameters are calculated along with the fixed effect parameters. Therefore the score vector $\boldsymbol{S}$ will be of length $p_{2}$ (the number of time-to-event sub-model fixed effect coefficients) plus the number of association parameters (one for each level of random effects included in the model). Consequently, if the model just contains individual level random effects, $\boldsymbol{S}$ will be of length $p_{2}+1$, whereas if the model contains both individual and study level random effects $\boldsymbol{S}$ will have length $p_{2}+2$. Note that the software requires at least one individual level random effect to be specified in the model (to ensure the presence of parameters to share between sub-models). The first $p_{2}$ elements of $\boldsymbol{S}$ equal the first differentiate of the expected log-likelihood with respect to each value in the vector of time-to-event fixed effect coefficients $\boldsymbol{\beta}_{\boldsymbol{2}}$, given the estimated values of the functions of any random effects. The remaining elements are the first differentiate with respect to each association parameter included in the model (ordered with the association parameter for the individual level random effects first, followed by that for the study level random effects if included).

The information matrix $\boldsymbol{I}$ is a square matrix that contains the negative of the second order differentiates of the expected log-likelihood, giving a matrix with number of rows and columns equal to $p_{2}$ plus the number of association parameters.

The estimates of the elements of $\boldsymbol{\beta}_{\boldsymbol{2}}$ can be updated at each iteration using equation (10), where $\nu$ is the iteration counter, such that ${\hat{\boldsymbol{\beta}}}_{2\left( \nu-1 \right)}$ are the estimates of the time-to-event sub-model fixed effect coefficients from iteration $\nu-1$. Furthermore, $\boldsymbol{S}_{\left( \nu-1 \right)}({\hat{\boldsymbol{\beta}}}_{2\left( \nu-1 \right)})$ is the score function, and $\boldsymbol{I}_{\left( \nu-1 \right)}^{-1}({\hat{\boldsymbol{\beta}}}_{2\left( \nu-1 \right)})$ the inverse of the information matrix, both based on the coefficient estimates ${\hat{\boldsymbol{\beta}}}_{2\left( \nu-1 \right)}$:

|  | ${\hat{\boldsymbol{\beta}}}_{2(\nu)}={\hat{\boldsymbol{\beta}}}_{2(\nu-1)}+\boldsymbol{I}_{\left( \nu-1 \right)}^{-1}({\hat{\boldsymbol{\beta}}}_{2\left( \nu-1 \right)})\boldsymbol{S}_{\left( \nu-1 \right)}({\hat{\boldsymbol{\beta}}}_{2\left( \nu-1 \right)})$ | (10) |
| --- | --- | --- |

The estimates of the association parameters are updated in a similar way. Below, the expressions necessary to calculate the score vector and the information matrix at each step are stated. The expressions are given for a case where both individual level and study level random effects have been included in the model (giving the score vector $\boldsymbol{S}$ a length of $p_{2}+2$, and the information matrix $\boldsymbol{I}$ row and column dimensions equal to $p_{2}+2$).

The first $p_{2}$ elements of $\boldsymbol{S}$, relate to the coefficients of the fixed effects in the time-to-event sub-model, and will have the form:

$$\boldsymbol{S}\left( \beta_{2g} \right)=\sum_{k=1}^{K} \sum_{i=1}^{n_{k}} \Delta_{ki}x_{2kig}-\sum_{k=1}^{K} \sum_{i=1}^{n_{k}} \int_{0}^{T_{Ski}} \lambda_{0}\left( u \right)\left( x_{2kig} \right)\exp\left( \boldsymbol{X}_{\boldsymbol{2}\boldsymbol{ki}}\boldsymbol{\beta}_{\boldsymbol{2}} \right)\mathbb{E}\left[ \exp\left( \alpha^{(2)}\left( \boldsymbol{Z}_{\boldsymbol{ki}}^{\boldsymbol{(2)}}\boldsymbol{b}_{\boldsymbol{ki}}^{\boldsymbol{(2)}} \right) \right) \right]\mathbb{E}\left[ \exp\left( \alpha^{(3)}\left( \boldsymbol{Z}_{\boldsymbol{ki}}^{\boldsymbol{(3)}}\boldsymbol{b}_{\boldsymbol{k}}^{\boldsymbol{(3)}} \right) \right) \right]du$$

In the above equation, $g$ takes values 1 to $p_{2}$. The remaining elements of the score vector relate to the association parameters for the individual level random effects ($\alpha^{\left( 2 \right)})$, and (if included) the study level random effects ($\alpha^{\left( 3 \right)}$), and take the following forms:

$$\boldsymbol{S}\left( \alpha^{(2)} \right)=\sum_{k=1}^{K} \sum_{i=1}^{n_{k}} \Delta_{ki}\mathbb{E}\left[ \left( \boldsymbol{Z}_{\boldsymbol{ki}}^{\boldsymbol{(2)}}\boldsymbol{b}_{\boldsymbol{ki}}^{\boldsymbol{(2)}} \right) \right]-\sum_{k=1}^{K} \sum_{i=1}^{n_{k}} \int_{0}^{T_{Ski}} \lambda_{0}\left( u \right)\exp\left( \boldsymbol{X}_{\boldsymbol{2}\boldsymbol{ki}}\boldsymbol{\beta}_{\boldsymbol{2}} \right)\mathbb{E}\left[ \left( \boldsymbol{Z}_{\boldsymbol{ki}}^{\boldsymbol{(2)}}\boldsymbol{b}_{\boldsymbol{ki}}^{\boldsymbol{(2)}} \right)\exp\left( \alpha^{(2)}\left( \boldsymbol{Z}_{\boldsymbol{ki}}^{\boldsymbol{(2)}}\boldsymbol{b}_{\boldsymbol{ki}}^{\boldsymbol{(2)}} \right) \right) \right]\mathbb{E}\left[ \exp\left( \alpha^{(3)}\left( \boldsymbol{Z}_{\boldsymbol{ki}}^{\boldsymbol{(3)}}\boldsymbol{b}_{\boldsymbol{k}}^{\boldsymbol{(3)}} \right) \right) \right]du$$

$$\boldsymbol{S}\left( \alpha^{(3)} \right)=\sum_{k=1}^{K} \sum_{i=1}^{n_{k}} \Delta_{ki}\mathbb{E}\left[ \left( \boldsymbol{Z}_{\boldsymbol{ki}}^{\boldsymbol{(3)}}\boldsymbol{b}_{\boldsymbol{k}}^{\boldsymbol{(3)}} \right) \right]-\sum_{k=1}^{K} \sum_{i=1}^{n_{k}} \int_{0}^{T_{Ski}} \lambda_{0}\left( u \right)\exp\left( \boldsymbol{X}_{\boldsymbol{2}\boldsymbol{ki}}\boldsymbol{\beta}_{\boldsymbol{2}} \right)\mathbb{E}\left[ \exp\left( \alpha^{(2)}\left( \boldsymbol{Z}_{\boldsymbol{ki}}^{\boldsymbol{(2)}}\boldsymbol{b}_{\boldsymbol{ki}}^{\boldsymbol{(2)}} \right) \right) \right]\mathbb{E}\left[ \left( \boldsymbol{Z}_{\boldsymbol{ki}}^{\boldsymbol{(3)}}\boldsymbol{b}_{\boldsymbol{k}}^{\boldsymbol{(3)}} \right)\exp\left( \alpha^{(3)}\left( \boldsymbol{Z}_{\boldsymbol{ki}}^{\boldsymbol{(3)}}\boldsymbol{b}_{\boldsymbol{k}}^{\boldsymbol{(3)}} \right) \right) \right]du$$

The information matrix $\boldsymbol{I}$ will have the following form:

$$\boldsymbol{I}=\left( \begin{matrix} I\left( \beta_{21} \right) & \cdots& I\left( \beta_{21},\beta_{2p_{2}} \right) & I\left( \beta_{21},\alpha^{(2)} \right) & I\left( \beta_{21},\alpha^{(3)} \right) \\ \vdots& \ddots& \vdots& \vdots& \vdots\\ I\left( \beta_{21},\beta_{2p_{2}} \right) & \cdots& I\left( \beta_{2p_{2}} \right) & I\left( \beta_{2p_{2}},\alpha^{(2)} \right) & I\left( \beta_{2p_{2}},\alpha^{(3)} \right) \\ I\left( \beta_{21},\alpha^{(2)} \right) & \cdots& I\left( \beta_{2p_{2}},\alpha^{(2)} \right) & I\left( \alpha^{(2)} \right) & I\left( \alpha^{(2)},\alpha^{(3)} \right) \\ I\left( \beta_{21},\alpha^{(3)} \right) & \cdots& I\left( \beta_{2p_{2}},\alpha^{(3)} \right) & I\left( \alpha^{(2)},\alpha^{(3)} \right) & I\left( \alpha^{(3)} \right) \end{matrix} \right)$$

Note that any row or column containing the study level association parameter $\alpha^{\left( 3 \right)}$ will not be present if study level random effects are not included in the model. The elements in the information matrix correspond to different expressions depending on their location in the matrix. The first $p_{2}$ values on the diagonal have form:

$$I\left( \beta_{2g} \right)=\sum_{k=1}^{K} \sum_{i=1}^{n_{k}} \int_{0}^{T_{Ski}} \lambda_{0}\left( u \right)\left( x_{2kig} \right)^{2}\exp\left( \boldsymbol{X}_{\boldsymbol{2}\boldsymbol{ki}}\boldsymbol{\beta}_{\boldsymbol{2}} \right)\mathbb{E}\left[ \exp\left( \alpha^{(2)}\left( \boldsymbol{Z}_{\boldsymbol{ki}}^{\boldsymbol{(2)}}\boldsymbol{b}_{\boldsymbol{ki}}^{\boldsymbol{(2)}} \right) \right) \right]\mathbb{E}\left[ \exp\left( \alpha^{(3)}\left( \boldsymbol{Z}_{\boldsymbol{ki}}^{\boldsymbol{(3)}}\boldsymbol{b}_{\boldsymbol{k}}^{\boldsymbol{(3)}} \right) \right) \right]du$$

Whilst the off-diagonals in the first $p_{2}$ rows and columns of the information matrix take form:

$$I\left( \beta_{2g},\beta_{2f} \right)=\sum_{k=1}^{K} \sum_{i=1}^{n_{k}} \int_{0}^{T_{Ski}} \lambda_{0}\left( u \right)\left( x_{2kig}x_{2kif} \right)\exp\left( \boldsymbol{X}_{\boldsymbol{2}\boldsymbol{ki}}\boldsymbol{\beta}_{\boldsymbol{2}} \right)\mathbb{E}\left[ \exp\left( \alpha^{(2)}\left( \boldsymbol{Z}_{\boldsymbol{ki}}^{\boldsymbol{(2)}}\boldsymbol{b}_{\boldsymbol{ki}}^{\boldsymbol{(2)}} \right) \right) \right]\mathbb{E}\left[ \exp\left( \alpha^{(3)}\left( \boldsymbol{Z}_{\boldsymbol{ki}}^{\boldsymbol{(3)}}\boldsymbol{b}_{\boldsymbol{k}}^{\boldsymbol{(3)}} \right) \right) \right]du$$

In the above expressions, $g$ and $f$ take values between 1 and $p_{2}$ (with $g\neq f$). The remaining on-diagonals take form:

$$I\left( \alpha^{(2)} \right)=\sum_{k=1}^{K} \sum_{i=1}^{n_{k}} \int_{0}^{T_{Ski}} \lambda_{0}\left( u \right)\exp\left( \boldsymbol{X}_{\boldsymbol{2ki}}\boldsymbol{\beta}_{\boldsymbol{2}} \right)\mathbb{E}\left[ \left( \boldsymbol{Z}_{\boldsymbol{ki}}^{\boldsymbol{(2)}}\boldsymbol{b}_{\boldsymbol{ki}}^{\boldsymbol{(2)}} \right)^{2}\exp\left( \alpha^{(2)}\left( \boldsymbol{Z}_{\boldsymbol{ki}}^{\boldsymbol{(2)}}\boldsymbol{b}_{\boldsymbol{ki}}^{\boldsymbol{(2)}} \right) \right) \right]\mathbb{E}\left[ \exp\left( \alpha^{(3)}\left( \boldsymbol{Z}_{\boldsymbol{ki}}^{\boldsymbol{(3)}}\boldsymbol{b}_{\boldsymbol{k}}^{\boldsymbol{(3)}} \right) \right) \right]du$$

$$I\left( \alpha^{(3)} \right)=\sum_{k=1}^{K} \sum_{i=1}^{n_{k}} \int_{0}^{T_{Ski}} \lambda_{0}\left( u \right)\exp\left( \boldsymbol{X}_{\boldsymbol{2}\boldsymbol{ki}}\boldsymbol{\beta}_{\boldsymbol{2}} \right)\mathbb{E}\left[ \exp\left( \alpha^{(2)}\left( \boldsymbol{Z}_{\boldsymbol{ki}}^{\boldsymbol{(2)}}\boldsymbol{b}_{\boldsymbol{ki}}^{\boldsymbol{(2)}} \right) \right) \right]\mathbb{E}\left[ \left( \boldsymbol{Z}_{\boldsymbol{ki}}^{\boldsymbol{(3)}}\boldsymbol{b}_{\boldsymbol{k}}^{\boldsymbol{(3)}} \right)^{2}\exp\left( \alpha^{(3)}\left( \boldsymbol{Z}_{\boldsymbol{ki}}^{\boldsymbol{(3)}}\boldsymbol{b}_{\boldsymbol{k}}^{\boldsymbol{(3)}} \right) \right) \right]du$$

The remaining off diagonal elements take the following forms, depending on their placement in the information matrix (see above for matrix structure).

$$I\left( \beta_{2g},\alpha^{(2)} \right)=\sum_{k=1}^{K} \sum_{i=1}^{n_{k}} \int_{0}^{T_{Ski}} \lambda_{0}\left( u \right)\left( x_{2kig} \right)\exp\left( \boldsymbol{X}_{\boldsymbol{2}\boldsymbol{ki}}\boldsymbol{\beta}_{\boldsymbol{2}} \right)\mathbb{E}\left[ \left( \boldsymbol{Z}_{\boldsymbol{ki}}^{\boldsymbol{(2)}}\boldsymbol{b}_{\boldsymbol{ki}}^{\boldsymbol{(2)}} \right)\exp\left( \alpha^{(2)}\left( \boldsymbol{Z}_{\boldsymbol{ki}}^{\boldsymbol{(2)}}\boldsymbol{b}_{\boldsymbol{ki}}^{\boldsymbol{(2)}} \right) \right) \right]\mathbb{E}\left[ \exp\left( \alpha^{(3)}\left( \boldsymbol{Z}_{\boldsymbol{ki}}^{\boldsymbol{(3)}}\boldsymbol{b}_{\boldsymbol{k}}^{\boldsymbol{(3)}} \right) \right) \right]du$$

$$I\left( \beta_{2g},\alpha^{(3)} \right)=\sum_{k=1}^{K} \sum_{i=1}^{n_{k}} \int_{0}^{T_{Ski}} \lambda_{0}\left( u \right)\left( x_{2kig} \right)\exp\left( \boldsymbol{X}_{\boldsymbol{2}\boldsymbol{ki}}\boldsymbol{\beta}_{\boldsymbol{2}} \right)\mathbb{E}\left[ \exp\left( \alpha^{(2)}\left( \boldsymbol{Z}_{\boldsymbol{ki}}^{\boldsymbol{(2)}}\boldsymbol{b}_{\boldsymbol{ki}}^{\boldsymbol{(2)}} \right) \right) \right]\mathbb{E}\left[ \left( \boldsymbol{Z}_{\boldsymbol{ki}}^{\boldsymbol{(3)}}\boldsymbol{b}_{\boldsymbol{k}}^{\boldsymbol{(3)}} \right)\exp\left( \alpha^{(3)}\left( \boldsymbol{Z}_{\boldsymbol{ki}}^{\boldsymbol{(3)}}\boldsymbol{b}_{\boldsymbol{k}}^{\boldsymbol{(3)}} \right) \right) \right]du$$

$$I\left( \alpha^{(2)},\alpha^{(3)} \right)=\sum_{k=1}^{K} \sum_{i=1}^{n_{k}} \int_{0}^{T_{Ski}} \lambda_{0}\left( u \right)\exp\left( \boldsymbol{X}_{\boldsymbol{2}\boldsymbol{ki}}\boldsymbol{\beta}_{\boldsymbol{2}} \right)\mathbb{E}\left[ \left( \boldsymbol{Z}_{\boldsymbol{ki}}^{\boldsymbol{(2)}}\boldsymbol{b}_{\boldsymbol{ki}}^{\boldsymbol{(2)}} \right)\exp\left( \alpha^{(2)}\left( \boldsymbol{Z}_{\boldsymbol{ki}}^{\boldsymbol{(2)}}\boldsymbol{b}_{\boldsymbol{ki}}^{\boldsymbol{(2)}} \right) \right) \right]\mathbb{E}\left[ \left( \boldsymbol{Z}_{\boldsymbol{ki}}^{\boldsymbol{(3)}}\boldsymbol{b}_{k}^{(3)} \right)\exp\left( \alpha^{(3)}\left( \boldsymbol{Z}_{\boldsymbol{ki}}^{\boldsymbol{(3)}}\boldsymbol{b}_{\boldsymbol{k}}^{\boldsymbol{(3)}} \right) \right) \right]du$$

Using these expressions that make up the score vector $\boldsymbol{S}$ and information matrix $\boldsymbol{I}$ along with the Newton-Raphson procedure stated in equation (10), the estimates for the time-to-event sub-model fixed effect coefficients $\boldsymbol{\beta}_{\boldsymbol{2}}$, and the association parameters $\alpha^{\left( 2 \right)}$ and $\alpha^{\left( 3 \right)}$, can be updated at each iteration.

### Estimation of the longitudinal measurement error variance

The maximum likelihood estimator for the measurement error variance $\sigma_{e}^{2}$ in the longitudinal sub-model is estimated using standard maximum likelihood theory. Displaying only the portion of likelihood that involves this parameter (with $\boldsymbol{\eta}_{\boldsymbol{ki}}=\boldsymbol{X}_{\boldsymbol{1}\boldsymbol{ki}}\boldsymbol{\beta}_{\boldsymbol{1}}\mathbb{+E}\left[ \boldsymbol{Z}_{\boldsymbol{ki}}^{\left( \boldsymbol{2} \right)}\boldsymbol{b}_{\boldsymbol{ki}}^{\left( \boldsymbol{2} \right)} \right]\mathbb{+E}\left[ \boldsymbol{Z}_{\boldsymbol{ki}}^{\left( \boldsymbol{3} \right)}\boldsymbol{b}_{\boldsymbol{k}}^{\left( \boldsymbol{3} \right)} \right]$):

|  | $\boldsymbol{L}\left( \boldsymbol{\Omega} \right)=\prod_{k=1}^{K} \prod_{i=1}^{n_{k}} \frac{1}{\sqrt{2\pi\sigma_{e}^{2}}}\exp\left\{ -\frac{\left( \boldsymbol{Y}_{\boldsymbol{ki}}-\boldsymbol{\eta}_{\boldsymbol{ki}} \right)^{2}}{2\sigma_{e}^{2}} \right\}$ $=\left( \frac{1}{\sqrt{2\pi\sigma_{e}^{2}}} \right)^{\sum_{k=1}^{K} n_{k}}\exp\left( -\frac{1}{2\sigma_{e}^{2}}\sum_{k=1}^{K} \sum_{i=1}^{n_{k}} \left( \boldsymbol{Y}_{\boldsymbol{ki}}-\boldsymbol{\eta}_{\boldsymbol{ki}} \right)^{2} \right)$ | (11) |
| --- | --- | --- |

Giving a log-likelihood of:

|  | $\mathcal{l}\left( \boldsymbol{\Omega} \right)=-\frac{\sum_{k=1}^{K} n_{k}}{2}\log\left( 2\pi\right)-\frac{\sum_{k=1}^{K} n_{k}}{2}\log\left( \sigma_{e}^{2} \right)-\frac{1}{{2\sigma}_{e}^{2}}\sum_{k=1}^{K} \sum_{i=1}^{n_{k}} \left( \boldsymbol{Y}_{\boldsymbol{ki}}-\boldsymbol{\eta}_{\boldsymbol{ki}} \right)^{2}$ | (12) |
| --- | --- | --- |

Taking the first derivative of equation (12) with respect to $\sigma_{e}^{2}$ and setting equal to zero gives:

|  | $0=-\frac{\sum_{k=1}^{K} n_{k}}{2\sigma_{e}^{2}}+\frac{1}{2\sigma_{e}^{4}}\sum_{k=1}^{K} \sum_{i=1}^{n_{k}} \left( \boldsymbol{Y}_{\boldsymbol{ki}}-\boldsymbol{\eta}_{\boldsymbol{ki}} \right)^{2}$ | (13) |
| --- | --- | --- |

Which rearranges to give the maximum likelihood estimator of the parameter $\sigma_{e}^{2}$:

|  | $\sigma_{e}^{2}=\frac{\sum_{k=1}^{K} \sum_{i=1}^{n_{k}} \left( \boldsymbol{Y}_{\boldsymbol{ki}}-\boldsymbol{\eta}_{\boldsymbol{ki}} \right)^{2}}{\sum_{k=1}^{K} n_{k}}$ | (14) |
| --- | --- | --- |

### Estimation of the covariance matrix for individual level random effects

The one-stage function available in *joineRmeta* requires the existence of at least one individual level random effect in the specified model. The composition of the Maximum Likelihood Estimate (MLE) for the variance of these random effects depends on the number of random effects included in the model.

If multiple individual level random effects are included in the model, they follow a multivariate zero mean normal distribution. Therefore the maximum likelihood estimate for the covariance matrix of this distribution, denoted $\hat{\boldsymbol{D}}$, is required. The methods to obtain this estimate rely on the methods presented in Wulfsohn and Tsiatis [78] to estimate parameters for the joint model, and Anderson and Olkin [218] who discuss methods to obtain maximum likelihood estimates for multivariate normal distributions.

In the complete data likelihood the only component to directly involve the covariance matrix $D$ for the individual specific random effects is $f\left( \boldsymbol{b}_{ki}^{(2)} | \boldsymbol{D} \right)$, (equation (15)).

|  | $L\left( \boldsymbol{\Omega} \right)\mathbb{=E}\left[ \prod_{k=1}^{K} \prod_{i=1}^{n_{k}} {\left( 2\pi\right)^{-q/2}\left\vert\boldsymbol{D} \right\vert}^{-1/2}\exp\left\{ -\frac{\left( \boldsymbol{b}_{ki}^{\left( 2 \right)} \right)^{T}\boldsymbol{D}^{-1}\left( \boldsymbol{b}_{ki}^{\left( 2 \right)} \right)}{2} \right\} \right]$ | (15) |
| --- | --- | --- |

The expectation (denoted by $\mathbb{E}$) is taken over the individual level random effects. The expected values of the required functions of the individual level random effects are calculated in the E-step of the EM algorithm. The log-likelihood is then:

|  | $\mathcal{l}\left( \boldsymbol{\Omega} \right)=-\sum_{k=1}^{K} \frac{qn_{k}}{2}\log\left( 2\pi\right)-\sum_{k=1}^{K} \frac{n_{k}}{2}\log\left\vert\boldsymbol{D} \right\vert-\frac{1}{2}\sum_{k=1}^{K} \sum_{i=1}^{n_{k}} \mathbb{E}\left[ \left( \boldsymbol{b}_{ki}^{\left( 2 \right)} \right)^{T}\boldsymbol{D}^{\boldsymbol{-1}}\left( \boldsymbol{b}_{ki}^{\left( 2 \right)} \right) \right]$ | (16) |
| --- | --- | --- |

In equation (16), consider $\mathbb{E}\left[ \left( \boldsymbol{b}_{ki}^{\left( 2 \right)} \right)^{T}\boldsymbol{D}^{-1}\left( \boldsymbol{b}_{ki}^{\left( 2 \right)} \right) \right]$ as the trace of a 1 by 1 matrix, leading to:

|  | $\approx-\sum_{k=1}^{K} \frac{n_{k}}{2}\log\left\vert\boldsymbol{D} \right\vert-\frac{1}{2}\sum_{k=1}^{K} \sum_{i=1}^{n_{k}} tr\left( \mathbb{E}\left[ \left( \boldsymbol{b}_{ki}^{\left( 2 \right)} \right)^{T}\boldsymbol{D}^{-1}\left( \boldsymbol{b}_{ki}^{\left( 2 \right)} \right) \right] \right)$ | (17) |
| --- | --- | --- |

Using that fact that $tr\left( \boldsymbol{GH} \right)=tr\left( \boldsymbol{HG} \right)$, rearranges to:

|  | $\approx-\sum_{k=1}^{K} \frac{n_{k}}{2}\log\left\vert\boldsymbol{D} \right\vert-\frac{1}{2}\sum_{k=1}^{K} \sum_{i=1}^{n_{k}} tr\left( \mathbb{E}\left[ \left( \boldsymbol{b}_{ki}^{\left( 2 \right)} \right)\left( \boldsymbol{b}_{ki}^{\left( 2 \right)} \right)^{T}\boldsymbol{D}^{-1} \right] \right)$ | (18) |
| --- | --- | --- |

Again, using $tr\left( \boldsymbol{GH} \right)=tr\left( \boldsymbol{HG} \right)$ gives:

|  | $\approx-\sum_{k=1}^{K} \frac{n_{k}}{2}\log\left\vert\boldsymbol{D} \right\vert-\frac{1}{2}tr\left( \boldsymbol{D}^{-1}\sum_{k=1}^{K} \sum_{i=1}^{n_{k}} \mathbb{E}\left[ \left( \boldsymbol{b}_{ki}^{\left( 2 \right)} \right)\left( \boldsymbol{b}_{ki}^{\left( 2 \right)} \right)^{T} \right] \right)$ | (19) |
| --- | --- | --- |

Defining $\boldsymbol{P}=\sum_{k=1}^{K} \sum_{i=1}^{n_{k}} \mathbb{E}\left[ \left( \boldsymbol{b}_{ki}^{\left( 2 \right)} \right)\left( \boldsymbol{b}_{ki}^{\left( 2 \right)} \right)^{T} \right]$, equation (44)is restated to:

|  | $\approx-\left( \sum_{k=1}^{K} \frac{n_{k}}{2} \right)\log\left\vert\boldsymbol{D} \right\vert-\frac{1}{2}tr\left( \boldsymbol{D}^{-1}\boldsymbol{P} \right)$ | (20) |
| --- | --- | --- |

From Anderson and Olkin [218], an expression $f\left( \boldsymbol{\Sigma},\boldsymbol{V} \right)=-\log\left| \boldsymbol{\Sigma} \right|-tr\left( \boldsymbol{\Sigma}^{-1}\boldsymbol{V} \right)$ has derivative with respect to elements of $\boldsymbol{\Sigma}$ (in matrix form) of $-\boldsymbol{\Sigma}^{-1}+\boldsymbol{\Sigma}^{-1}\boldsymbol{V}\boldsymbol{\Sigma}^{-1}$. Therefore, the first derivative of equation (45), set equal to zero, (taking into account that the values of $\sum_{k=1}^{K} \frac{n_{k}}{2}$ and $\frac{1}{2}$ remain constant when differentiating with respect to the elements of $\boldsymbol{D}$) is:

|  | $-\left( \sum_{k=1}^{K} \frac{n_{k}}{2} \right)\boldsymbol{D}^{-1}+\frac{1}{2}\boldsymbol{D}^{-1}\boldsymbol{P}\boldsymbol{D}^{-1}=0$ $\frac{1}{2}\boldsymbol{D}^{-1}\boldsymbol{P}\boldsymbol{D}^{-1}=\left( \sum_{k=1}^{K} \frac{n_{k}}{2} \right)\boldsymbol{D}^{-1}$ $\boldsymbol{D}^{-1}\boldsymbol{P}\boldsymbol{D}^{-1}=\left( \sum_{k=1}^{K} n_{k} \right)\boldsymbol{D}^{-1}$ $\boldsymbol{D}^{-1}\boldsymbol{P}=\left( \sum_{k=1}^{K} n_{k} \right)$ $\boldsymbol{P}=\boldsymbol{D}\left( \sum_{k=1}^{K} n_{k} \right)$ $\hat{\boldsymbol{D}}=\frac{\sum_{k=1}^{K} \sum_{i=1}^{n_{k}} \mathbb{E}\left[ \left( \boldsymbol{b}_{ki}^{\left( 2 \right)} \right)\left( \boldsymbol{b}_{ki}^{\left( 2 \right)} \right)^{T} \right]}{\sum_{k=1}^{K} n_{k}}$ | (21) |
| --- | --- | --- |

Equation (21) states the maximum likelihood estimate for the covariance matrix $\boldsymbol{D}$, for the case where two or more individual level random effects have been included in the model to be fitted by the one-stage function. If the model contained only one individual level random effect, then $\boldsymbol{D}$ would be a single value, termed $\sigma_{D}^{2}$). In this case, the relevant portion of the likelihood has the form:

|  | $L\left( \boldsymbol{\Omega} \right)\mathbb{=E}\left[ \prod_{k=1}^{K} \prod_{i=1}^{n_{k}} \left( 2\pi\sigma_{D}^{2} \right)^{-1/2}\exp\left( {{-\left( \boldsymbol{b}_{kI}^{(2)} \right)}^{2}}/{2\sigma_{D}^{2}} \right) \right]$ | (22) |
| --- | --- | --- |

Then the log-likelihood:

|  | $\mathcal{l}\left( \boldsymbol{\Omega} \right)=-\sum_{k=1}^{K} \frac{n_{k}}{2}\log\left( 2\pi\right)-\sum_{k=1}^{K} \frac{n_{k}}{2}\log\left( \sigma_{D}^{2} \right)-\frac{1}{2\sigma_{D}^{2}}\sum_{k=1}^{K} \sum_{i=1}^{n_{k}} \mathbb{E}\left[ \left( \boldsymbol{b}_{ki}^{\left( 2 \right)} \right)^{2} \right]$ | (23) |
| --- | --- | --- |

Giving a first derivative with respect to $\sigma_{D}^{2}$ of:

|  | $\mathcal{l}^{'\left( \boldsymbol{\Omega} \right)}=-\sum_{k=1}^{K} \frac{n_{k}}{2\sigma_{D}^{2}}+\frac{1}{2\sigma_{D}^{4}}\sum_{k=1}^{K} \sum_{i=1}^{n_{k}} \mathbb{E}\left[ \left( \boldsymbol{b}_{ki}^{\left( 2 \right)} \right)^{2} \right]$ | (24) |
| --- | --- | --- |

Which, when set to zero and rearranged gives an MLE for $\sigma_{D}^{2}$ of:

|  | $\sigma_{D}^{2}=\frac{\sum_{k=1}^{K} \sum_{i=1}^{n_{k}} \mathbb{E}\left[ \left( \boldsymbol{b}_{ki}^{\left( 2 \right)} \right)^{2} \right]}{\sum_{k=1}^{K} n_{k}}$ | (25) |
| --- | --- | --- |

### Estimation of the covariance matrix for study level random effects

It has already been noted that when no study specific (level 3) random effects are included in the model, the $f\left( b_{k}^{(3)} | \boldsymbol{A} \right)$ term is not included in the complete data likelihood. If only one study level random effect is included in the model, then MLE of the variance of the study level random effect, denoted $\sigma_{A}^{2}$, can be determined as follows. The likelihood takes the form:

|  | $L\left( \boldsymbol{\Omega} \right)\mathbb{=E}\left[ \prod_{k=1}^{K} \left( 2\pi\sigma_{A}^{2} \right)^{-1/2}\exp\left( {{-\left( \boldsymbol{b}_{k}^{(3)} \right)}^{2}}/{2\sigma_{A}^{2}} \right) \right]$ | (26) |
| --- | --- | --- |

Giving log-likelihood:

|  | $\mathcal{l}\left( \boldsymbol{\Omega} \right)=-\frac{K}{2}\log\left( 2\pi\right)-\frac{K}{2}\log\left( \sigma_{A}^{2} \right)-\frac{1}{2\sigma_{A}^{2}}\sum_{k=1}^{K} \mathbb{E}\left[ \left( \boldsymbol{b}_{k}^{(3)} \right)^{2} \right]$ | (27) |
| --- | --- | --- |

Taking the first derivative of equation (27), setting equal to zero and rearranging, gives the MLE for $\sigma_{A}^{2}$:

|  | $\sigma_{A}^{2}=\frac{\sum_{k=1}^{K} \mathbb{E}\left[ \left( \boldsymbol{b}_{k}^{(3)} \right)^{2} \right]}{K}$ | (28) |
| --- | --- | --- |

If more than one study level random effect is included in the model, the relevant portion of the likelihood takes the form:

|  | $L\left( \boldsymbol{\Omega} \right)\mathbb{=E}\left[ \prod_{k=1}^{K} {{(2\pi)}^{-r/2}\left\vert\boldsymbol{A} \right\vert}^{-1/2}exp\left\{ -\frac{\left( \boldsymbol{b}_{k}^{(3)} \right)^{T}\boldsymbol{A}^{-1}\left( \boldsymbol{b}_{k}^{(3)} \right)}{2} \right\} \right]$ | (29) |
| --- | --- | --- |

The MLE for the covariance matrix $\boldsymbol{A}$ is derived in a similar way to that for individual level random effects matrix $\boldsymbol{D}$. Rearranging to the log-likelihood gives:

|  | $\mathcal{l}\left( \boldsymbol{\Omega} \right)=-\frac{K}{2}\log\left( 2\pi\right)-\frac{K}{2}\log\left\vert\boldsymbol{A} \right\vert-\frac{1}{2}\sum_{k=1}^{K} \mathbb{E}\left[ \left( \boldsymbol{b}_{k}^{\left( 3 \right)} \right)^{T}\boldsymbol{A}^{-1}\left( \boldsymbol{b}_{k}^{\left( 3 \right)} \right) \right]$ $\approx-\frac{K}{2}\log\left\vert\boldsymbol{A} \right\vert-\frac{1}{2}\sum_{k=1}^{K} \mathbb{E}\left[ \left( \boldsymbol{b}_{k}^{\left( 3 \right)} \right)^{T}\boldsymbol{A}^{-1}\left( \boldsymbol{b}_{k}^{\left( 3 \right)} \right) \right]$ | (30) |
| --- | --- | --- |

As before, consider $\mathbb{E}\left[ \left( \boldsymbol{b}_{k}^{\left( 3 \right)} \right)^{'}\boldsymbol{A}^{-1}\left( \boldsymbol{b}_{k}^{\left( 3 \right)} \right) \right]$ to be the trace of a 1 by 1 matrix. Using this, and the fact that $tr\left( \boldsymbol{GH} \right)=tr\left( \boldsymbol{GH} \right)$, allows:

|  | $=-\frac{K}{2}\log\left\vert\boldsymbol{A} \right\vert-\frac{1}{2}\sum_{k=1}^{K} tr\left( \mathbb{E}\left[ \left( \boldsymbol{b}_{k}^{\left( 3 \right)} \right)^{T}\boldsymbol{A}^{-1}\left( \boldsymbol{b}_{k}^{\left( 3 \right)} \right) \right] \right)$ $=-\frac{K}{2}\log\left\vert\boldsymbol{A} \right\vert-\frac{1}{2}\sum_{k=1}^{K} tr\left( \mathbb{E}\left[ \left( \boldsymbol{b}_{k}^{\left( 3 \right)} \right)\left( \boldsymbol{b}_{k}^{\left( 3 \right)} \right)^{T}\boldsymbol{A}^{-1} \right] \right)$ $=-\frac{K}{2}\log\left\vert\boldsymbol{A} \right\vert-\frac{1}{2}tr\left( \boldsymbol{A}^{-1}\sum_{k=1}^{K} \mathbb{E}\left[ \left( \boldsymbol{b}_{k}^{\left( 3 \right)} \right)\left( \boldsymbol{b}_{k}^{\left( 3 \right)} \right)^{T} \right] \right)$ | (31) |
| --- | --- | --- |

Then, setting $\boldsymbol{R}= \sum_{k=1}^{K} \mathbb{E}\left[ \left( \boldsymbol{b}_{k}^{\left( 3 \right)} \right)\left( \boldsymbol{b}_{k}^{\left( 3 \right)} \right)^{T} \right]$ simplifies to:

|  | $=-\frac{K}{2}\log\left\vert\boldsymbol{A} \right\vert-\frac{1}{2}tr\left( \boldsymbol{A}^{-1}\boldsymbol{R} \right)$ | (32) |
| --- | --- | --- |

Again, using Anderson and Olkin [218], the first derivative of this expression with respect to the elements of $A$ can be found. By setting equal to zero and rearranging the MLE for the covariance matrix of the study level random effects $\boldsymbol{A}$ can be expressed.

|  | $\mathcal{l}^{'}\left( \boldsymbol{\Omega} \right)=-\frac{K}{2}\boldsymbol{A}^{-1}-\frac{1}{2}\boldsymbol{A}^{-1}\boldsymbol{R}\boldsymbol{A}^{-1}$ $0=-\frac{K}{2}\boldsymbol{A}^{-1}-\frac{1}{2}\boldsymbol{A}^{-1}\boldsymbol{R}\boldsymbol{A}^{-1}$ $\hat{\boldsymbol{A}}=\frac{\boldsymbol{R}}{K}=\frac{\sum_{k=1}^{K} \mathbb{E}\left[ \left( \boldsymbol{b}_{k}^{\left( 3 \right)} \right)\left( \boldsymbol{b}_{k}^{\left( 3 \right)} \right)^{T} \right]}{K}$ | (33) |
| --- | --- | --- |
